# Rationalization and Design of the Complementarity Determining Region Sequences in an Antibody-Antigen Recognition Interface

**DOI:** 10.1371/journal.pone.0033340

**Published:** 2012-03-22

**Authors:** Chung-Ming Yu, Hung-Pin Peng, Ing-Chien Chen, Yu-Ching Lee, Jun-Bo Chen, Keng-Chang Tsai, Ching-Tai Chen, Jeng-Yih Chang, Ei-Wen Yang, Po-Chiang Hsu, Jhih-Wei Jian, Hung-Ju Hsu, Hung-Ju Chang, Wen-Lian Hsu, Kai-Fa Huang, Alex Che Ma, An-Suei Yang

**Affiliations:** 1 Genomics Research Center, Academia Sinica, Taipei, Taiwan; 2 Institute of Biomedical Informatics, National Yang-Ming University, Taipei, Taiwan; 3 Department of Computer Science, National Tsing-Hua University, Hsinchu, Taiwan; 4 Institute of Bioinformatics and Systems Biology, National Chiao-Tung University, Hsinchu, Taiwan; 5 Institute of Information Sciences, Academia Sinica, Taipei, Taiwan; 6 Graduate Institute of Life Sciences, National Defense University, Taipei, Taiwan; 7 Institute of Biochemical Science, National Taiwan University, Taipei, Taiwan; 8 Institute of Biological Chemistry, Academia Sinica, Taipei, Taiwan; 9 Bioinformatics Program, Taiwan International Graduate Program, Institute of Information Science, Academia Sinica, Taipei, Taiwan; 10 Chemical Biology and Molecular Biophysics Program, Taiwan International Graduate Program, Institute of Biological Chemistry, Academia Sinica, Taipei, Taiwan; Monash University, Australia

## Abstract

Protein-protein interactions are critical determinants in biological systems. Engineered proteins binding to specific areas on protein surfaces could lead to therapeutics or diagnostics for treating diseases in humans. But designing epitope-specific protein-protein interactions with computational atomistic interaction free energy remains a difficult challenge. Here we show that, with the antibody-VEGF (vascular endothelial growth factor) interaction as a model system, the experimentally observed amino acid preferences in the antibody-antigen interface can be rationalized with 3-dimensional distributions of interacting atoms derived from the database of protein structures. Machine learning models established on the rationalization can be generalized to design amino acid preferences in antibody-antigen interfaces, for which the experimental validations are tractable with current high throughput synthetic antibody display technologies. Leave-one-out cross validation on the benchmark system yielded the accuracy, precision, recall (sensitivity) and specificity of the overall binary predictions to be 0.69, 0.45, 0.63, and 0.71 respectively, and the overall Matthews correlation coefficient of the 20 amino acid types in the 24 interface CDR positions was 0.312. The structure-based computational antibody design methodology was further tested with other antibodies binding to VEGF. The results indicate that the methodology could provide alternatives to the current antibody technologies based on animal immune systems in engineering therapeutic and diagnostic antibodies against predetermined antigen epitopes.

## Introduction

Antibody has become the most prominent class of protein therapeutics and diagnostics [1], [2]. However, the underlying protein recognition principles have yet to be understood to the level, whereby an antibody-antigen recognition interface can be designed *de novo*. Although powerful high throughput recombinant protein library techniques capable of exploring more than one billion sequence variants in a single experiment have been providing platforms for protein-protein interaction engineering [3], [4], [5], the experimental capabilities are nevertheless infinitesimal in comparison with the vast combinatorial sequence space in a typical protein-protein interaction interface. Hence, current antibody discoveries are largely limited by the uncontrollable animal immune systems [6].

Computational capabilities on antibody design have been demonstrated to explore sequence space than is possible experimentally, but the focus has been largely limited on affinity maturation of existing antibody-antigen interactions. It has been shown that, with iterative computational design procedure focusing on single mutations, affinity of two antibodies has been improved by one to two orders of magnitude [7]. Computational antibody-antigen complex models have also been used in combination with phage display mutagenesis on a few selected CDR residues to improve antibody binding affinity by two orders of magnitude [8]. High-affinity antibodies can also be further improved by one order of magnitude with structure-based computational design [9]. De novo paratope design on antibodies against any targeted epitope of an antigen has been developed with computational modeling of CDR structures against the selected epitope [10], but the experimental verification of the computational capability has yet to be demonstrated.

Successful de novo computational designs on protein-protein interactions has been established, indicating that the current computational methodologies on protein structural energetics are able to identify feasible designs among vast possibilities [11] (see also reviews [12], [13] and references therein). Nevertheless, the accuracy of current energetic functions [7], [12], [13], [14], [15], [16], [17] has been a formidable barrier [18]. In particular, calculating interaction energetics involving water molecules in protein complex formation has been difficult [12], [16], [17], [18], [19], [20], [21], [22], [23], [24], [25]. As a consequence, the capability of ranking a series of tentative sequences near the optimal designs for protein-protein interface remains a difficult challenge [11].

Experimental platforms based on phage display of synthetic antibody libraries provide rich information on antibody-antigen interactions [5]. In this work, the aim is to use the data generated from phage-displayed antibody libraries to develop and calibrate computational tools for rational design of antibodies. To this end, we first exhaustively identified the interface CDR (complementarity determining region) sequence preferences in an antibody-VEGF (vascular endothelial growth factor) interaction system with experiments based on phage-displayed recombinant antibody libraries, and then used a structural informatics-based system to rationalize the CDR sequence preferences at atomic resolution with computational molecular modeling. The rationalization led to insights for a machine learning methodology, aiming at designing interface CDR sequences against designated epitopes on antigens of known structure. The results suggest that computational antibody design could effectively empower the high throughput recombinant protein library-based technologies.

## Results and Discussion

### Experimental antibody-VEGF interface sequences

**Figure 1:**
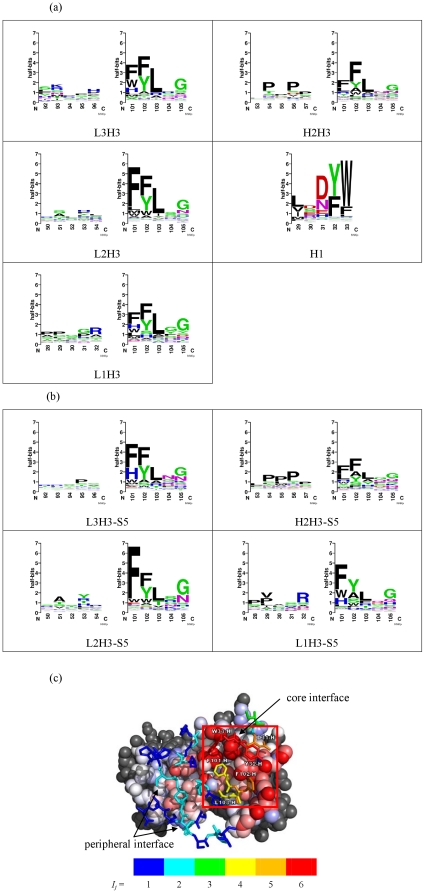
CDR sequence profiles and distribution of the amino acid preference stringency in the scFv-VEGF binding. (a) The data were derived from the screening of the five scFv libraries (L3H3, H2H3, L2H3, L1H3, and H1, see also Table S1). The computation of the LOGO plots is based on the formulation by Gorodkin et al [53] with modifications for amino acid background probabilities in phage display libraries and for pseudo counts as shown in Equation (2) of Text S1. (b) The data were derived from the screening of the four sc-dsFv libraries (L3H3-S5, H2H3-S5, L2H3-S5 and L1H3-S5, Table S2). (c) The interface structure of the antibody-VEGF is depicted based on the G6-Fab-VEGF complex structure (2FJG), where the 30 CDR interface residues are shown in stick model and the VEGF homodimer structure (V and W chains) shown in sphere model. The distribution of the color-coded CDR residues shows the position-dependence of the amino acid preferences towards VEGF-binding. The core interface region (boxed in red square) contains residues with high stringency in amino acid type requirement comparing with the residues in the peripheral interface region. The CDR residues are color-coded based on the information content (*I_j_*, as shown in the y-axis of the panel (a), is defined in Equation (2) of the Text S1). The color code for the background VEGF molecule is described in Figure 3(a).

The experimental amino acid preferences of antibody CDRs binding to VEGF were elucidated with VEGF-binding scFv/sc-dsFv variants derived from the G6 Fab-VEGF complex [26] as a model system. Nine synthetic scFv (single chain variable fragment [4]) or sc-dsFv (single chain disulfide stabilized variable fragment [27]) libraries were constructed with a recombinant phage display system to systematically randomize 5 consecutive residues on each of the 6 CDRs on the variable domains [27]; more than 500 variants for which the scFv/sc-dsFv expressed on bacterial phage surfaces are able to bind to VEGF with high affinity were systematically discovered with high throughput phage display selection and screening [27], [28]. The amino acid preferences of the 30 CDR interface residues in the scFv variants binding to VEGF are shown in Figure 1(a); the VEGF-binding data and the sequence details of the selected variants are shown in Table S1. Figure 1(b) and Table S2 show the VEGF-binding sequence patterns in sc-dsFv variants obtained with the corresponding sc-dsFv libraries. As shown in Figure 1(c), the amino acid preferences are position-dependent; a cluster of interface positions forms the core interface region, where amino acid type conservation for antigen binding is much more stringent than the peripheral interface.

**Figure 2:**
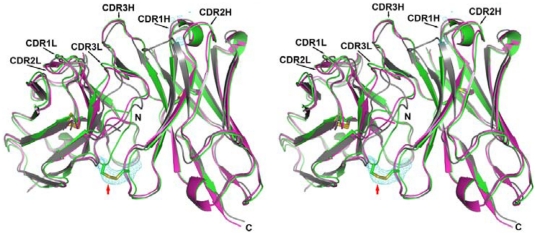
The crystallographic structure of the sc-dsFv derived from G6-Fab. The sc-dsFv structure (colored in green, PDB code 3AUV) is superimposed with the variable domains of VEGF-complexed G6-Fab (2FJG in PDB code, colored in grey) and unbound G6-Fab (2FGF in PDB code, colored in magenta). The interface disulfide bond in the sc-dsFv is marked with the arrow. The RMSDs between the sc-dsFv and the variable domains derived from 2FJF and 2FJG are 0.629 Å and 0.942 Å, respectively. The model of the interface disulfide bond in the sc-dsFv is shown with the superimposition of the *Fo-Fc* simulated annealing omit density map (colored in cyan) at the 5.0σ level. The omit density map was calculated without the residues of the interface cysteins. The refinement data for the sc-dsFv structure determination are shown in Table S3.

Since the phage display systems in Figure 1 are based on the scFv and the sc-dsFv scaffolds, it is important to verify that both the scFv and the sc-dsFv structures are similar to the variable domain structure in G6-Fab even in the absence of the two Fab constant domains in the scFv or the sc-dsFv structures. High-resolution sc-dsFv structure with the sequence identical to the parent G6-Fab variable domains (except for the two interface cysteines in the sc-dsFv shown in Figure 2) has been elucidated with x-ray crystallography as shown in Figure 2 (PDB code 3AUV); the refinement data are shown in Table S3. The corresponding scFv structure has not been attainable experimentally due to high aggregation tendency of the scFv in crystallization conditions. The comparison of the antibody variable domains in the uncomplexed and the VEGF-complexed G6-Fab structure (obtained from 2FJF and 2FJG, respectively, in PDB) with the sc-dsFv structure shown in Figure 2 suggests that the variable domain structures in the G6-Fab and in the sc-dsFv are largely identical to the atomic details. The structural differences of the engineered interface disulfide bond unique to the sc-dsFv structure are also highlighted in Figure 2. Although the G6-derived scFv structure has not been determined with experimental method, the consistency of the sc-dsFv structure with the G6-Fab structure as shown in Figure 2 and the consistency of the sequence patterns for the scFv and sc-dsFv variants binding to VEGF (comparison of Figures 1(a) and 1(b)) indicate that the scFv-VEGF interactions can be modeled based on the G6 Fab-VEGF complex structure as well.

The scFv/sc-dsFv libraries were designed with an internal control in each of the libraries to ensure that the amino acid preferences derived from the VEGF-binding variants are relevant to the complex structure, even when some of the CDR residues in the antibody fragment variants are different from the template G6-Fab sequence. As shown in Figure 1, each of the scFv/sc-dsFv libraries (except for the H1 library) was constructed with two separate random sequence regions simultaneously: one of the randomized regions contains 5 consecutive degenerate codons (NNK) in one of the four CDRs – CDR1L, CDR2L, CDR3L, and CDR2H; the other randomized region always contains 5 consecutive variable positions (also diversified with the NNK degenerate codon) in CDR3H. This design is based on the prior knowledge that the binding of the G6-derived scFv/sc-dsFv with VEGF is primarily anchored with the residues in CDR1H and CDR3H [27], [28]. With the residues in CDR1H remain constant as in G6-Fab in all the variants of the libraries (except for H1 library where the CDR3H residues remain constant as in G6-Fab), VEGF-binding sequence patterns emerged for the CDR3H variable region served as an indication to verify if the antibody-VEGF complex structure remains relevant for the selected variants in binding to the VEGF. As shown in Figures 1(a) and 1(b), the sequence patterns of the CDR3H region for the variants binding to VEGF are all in good agreement in the conservation of the anchoring residues in CDR3H (F101, F102, and L103), suggesting that the sequence variations in the CDRs for the scFv/sc-dsFv variants binding to VEGF did not variegate substantially the binding mode of the antibody variable domains to VEGF, mostly due to the anchoring of the scFv/sc-dsFv variants onto the VEGF binding site with the conserved anchoring residues in the CDR3H and CDR1H. Moreover, competition test of the phage-displayed scFv binding to VEGF with soluble non-fusion G6-derived scFv indicated that 34 out of the 37 selected phage displayed scFvs from the L2H3 library (Table S1) showed clear competition by the soluble scFv (up to 4 µM) on VEGF-binding, assuring that the scFv variants shown in Figure 1 bind to VEGF at mostly the same binding site as in the G6-Fab-VEGF complex. Taken together, the amino acid sequence preferences (LOGOs in Figure 1) for all the CDR positions in consideration are consistently relevant to the model antibody-antigen complex structure.

### Rationalization of the interface CDR sequence preferences

The amino acid preferences of the scFv interface CDRs binding to VEGF, as shown in Figure 1(a), are quantitatively represented by *W_ji_* (shown in Table 1), which is the log-odd-ratio of the probability of amino acid type *i* at CDR position *j* over the background probability of the amino acid type in the phage display system (Equation (5) in Methods). The working hypothesis is that *W_ji_* is linearly correlated with one or a combination of the following three statistically derived log-odd-ratio terms: *X_ji_*, *Y_ji_*, and *Z_ji_* (Equations (1)∼(4) in Methods), where *X_ji_* is the upper bound of the atomistic contact term for amino acid *i* at position *j*; *Y_ji_* reflects the maximal desolvation energy penalty due to the amino acid *i* at position *j* in forming the protein-protein complex; *Z_ji_* is the structural propensity for amino acid *i* in position *j* of the antibody CDR. One first-order approximation embedded in the working hypothesis is that the amino acid preferences (*W_ji_*) of position *j* is intrinsically dependent on the local antibody-antigen structural environment around the position *j*; higher order cooperative interactions due to the neighboring CDR residues are intractable in this approach. Following this approximation, the *X_ji_* and *Y_ji_* terms were calculated with the antibody structural models where the interface CDR position *j* was enumerated with all rotamers of amino acid type *i* while all other positions were reduced to alanine, as described in Equations (1)∼(4) in Methods, so as to mimic realistic antibody design situations where CDR sequences are not known. The numerical results of *W_ji_*, *X_ji_*, *Y_ji_*, and *Z_ji_* are shown in Table S4.

**Table 1:** Comparison of the predicted and the experimental amino acid preferences at each of the CDR interface positions.

28-L		29-L		30-L		31-L		32-L	
W_ji_	pW_ji_-t_i_	W_ji_	pW_ji_-t_i_	W_ji_	pW_ji_-t_i_	W_ji_	pW_ji_-t_i_	W_ji_	pW_ji_-t_i_
**P3.9**	**S .61**	**P4.2**	D .42	**G3.4**	**S .65**	**G4.3**	R .85	**R4.2**	F .43
**A2.8**	N .56	G2.4	**A .28**	F2.1	**A .57**	P3.7	S .68	**A2.4**	**Y .42**
**S1.4**	**P .47**	M2.1	N .18	**S1.7**	N .36	**N1.0**	A .57	**Y1.1**	H .37
**E1.0**	F .40	**H1.0**	**T .13**	**A1.5**	H .31	T0.2	H .49	W1.0	N .36
**M1.0**	**A .19**	**I1.0**	R .10	**P1.5**	**P .23**		**G .30**	**G0.9**	**G .25**
T0.9	H .17	**T0.9**	**L .08**		L .18		**N .20**	**T0.2**	**T .14**
V0.2	**E .07**	**L0.5**	**H .07**		Y .07		Y .07		**R .06**
	**M .00**	**A0.2**	**I .05**		**G .02**				**A .00**
			**P .04**		R .01				

50-L		51-L		52-L		53-L		54-L	
W_ji_	pW_ji_-t_i_	W_ji_	pW_ji_-t_i_	W_ji_	pW_ji_-t_i_	W_ji_	pW_ji_-t_i_	W_ji_	pW_ji_-t_i_
E2.5	Y .43	A3.6	N/A	N2.5	N/A	**H4.4**	R .59	H3.3	N/A
**G2.0**	**G .37**	S2.8		W2.5		**Y4.0**	N .56	N2.5	
**R1.8**	**S .37**	K1.4		L1.8		**F3.9**	G .38	L1.8	
**S0.9**	**R .33**	M1.4		S1.8		M2.5	S .34	I1.4	
Q0.6	H .30	G1.4		H1.4		I1.4	A .33	S1.4	
T0.6	N .26	T1.4		K1.4		W1.4	**H .21**	P1.4	
**L0.4**	A .21			R0.4			D .17	R0.9	
	**L .01**						P .08	A0.6	
							**F .06**E .04**Y .03**		

92-L		93-L		94-L		95-L		96-L	
W_ji_	pW_ji_-t_i_	W_ji_	pW_ji_-t_i_	W_ji_	pW_ji_-t_i_	W_ji_	pW_ji_-t_i_	W_ji_	pW_ji_-t_i_
**F4.3**	A .93	K4.9	**S .65**	P2.9	**R .68**	P3.2	N/A	H5.7	N/A
**Y3.6**	**S .60**	**N3.9**	H .58	**R1.6**	L .66	K1.6		Y2.4	
**H3.0**	P .38	M3.5	G .45	**A1.5**	Y .36	N1.6		F2.4	
**N3.0**	**N .35**	I3.0	A .29	**G1.1**	N .30	S1.6		P2.0	
I2.4	**H .25**	**R1.6**	**N .27**	**H0.5**	**H .29**	T1.5		M1.6	
**S2.1**	**F .19**	T1.1	**R .24**	I0.5	**G .25**	A1.1		N0.5	
L1.2	**Y .13**	**S0.9**	P .05	T0.5	**A .18**	F0.5		E0.5	
				**S0.01**	**S .17**			A0.5	

29-H		30-H		31-H		32-H		33-H	
W_ji_	pW_ji_-t_i_	W_ji_	pW_ji_-t_i_	W_ji_	pW_ji_-t_i_	W_ji_	pW_ji_-t_i_	W_ji_	pW_ji_-t_i_
I4.9	N/A	**D5.2**	**S .56**	D8.0	S .41	**Y8.3**	**Y .52**	W9.1	Y .45
L4.2		**E4.9**	P .24	**N5.5**	P .13	**F6.9**	**F .44**	F4.1	G .44
M3.0		**N3.0**	**D .22**	E3.0	**N .01**		H .41		H .32
V2.9		**S1.8**	A .19	H2.2			P .28		N .26
F2.2		Y1.2	H .18				N .27		A .19
		**R0.03**	**N .14**				A .23		R .19
			**R .02**						S .17
			**E .01**						

53-H		54-H		55-H		56-H		57-H	
W_ji_	pW_ji_-t_i_	W_ji_	pW_ji_-t_i_	W_ji_	pW_ji_-t_i_	W_ji_	pW_ji_-t_i_	W_ji_	pW_ji_-t_i_
K2.2	D .51	**P5.5**	**P .60**	**P3.1**	**P .42**	**P5.5**	G .59	**P4.3**	Y .63
L2.0	Y .38	**N2.2**	E .51	**D2.2**	E .36	D2.7	**P .14**	**A1.9**	**F .60**
**N1.6**	**P .28**	T1.9	A .26	**N2.2**	A .28	I1.6	H .14	**S1.1**	M .47
G1.6	H .27		**N .17**	**G2.2**	**D .20**	V0.3	N .13	**F0.9**	N .40
**P1.2**	E .16		H .11	H1.6	**G .08**	S0.2		**D0.9**	R .28
T0.3	**N .04**		D .06	I1.6	**N .01**			**H0.9**	**H .23**
	S .02			F0.9				E0.9	Q .18
				M0.9					**P .11**
									**A .11**L .04
									**S .01**
									**D .00**

101-H		102-H		103-H		104-H		105-H	
W_ji_	pW_ji_-t_i_	W_ji_	pW_ji_-t_i_	W_ji_	pW_ji_-t_i_	W_ji_	pW_ji_-t_i_	W_ji_	pW_ji_-t_i_
**F7.3**	P .59	**F7.5**	L .50	**L5.8**	R .47	**N4.3**	A .42	**G5.8**	**G .42**
W5.4	**F .48**	Y6.8	G .43	**A0.5**	G .34	**G2.0**	**H .33**	**N1.6**	A .38
**H3.9**	Y .35	M0.5	D .32		Y .31	Q2.0	P .30	**S0.9**	R .36
**M1.6**	N .32		P .29		N .29	**F1.6**	**G .29**	**Y0.6**	**Y .24**
	E .31		N .22		P .26	**H1.6**	**Y .27**	**H0.5**	**N .20**
	D .30		H .20		H .25	**R1.6**	**N .19**		**H .16**
	L .20		**F .17**		**A .16**	**S0.9**	**F .19**		L .10
	**H .12**		E .03		**L .04**	**Y0.6**	**R .16**		**S .05**
	A .06**M .02**					D0.5	**S .10**L .05		

At each position, the left-hand column shows the amino acid type and the corresponding *W_ji_*. All the predicted positive amino acid types (δ*pW_ji_* = 1) and the activation values for sequence preference (*pW_ji_*) are shown in the right-hand column. The amino acid types shown in bold are the common amino acid types (true positives) shown in both the left-hand and the right-hand columns.

**Figure 3:**
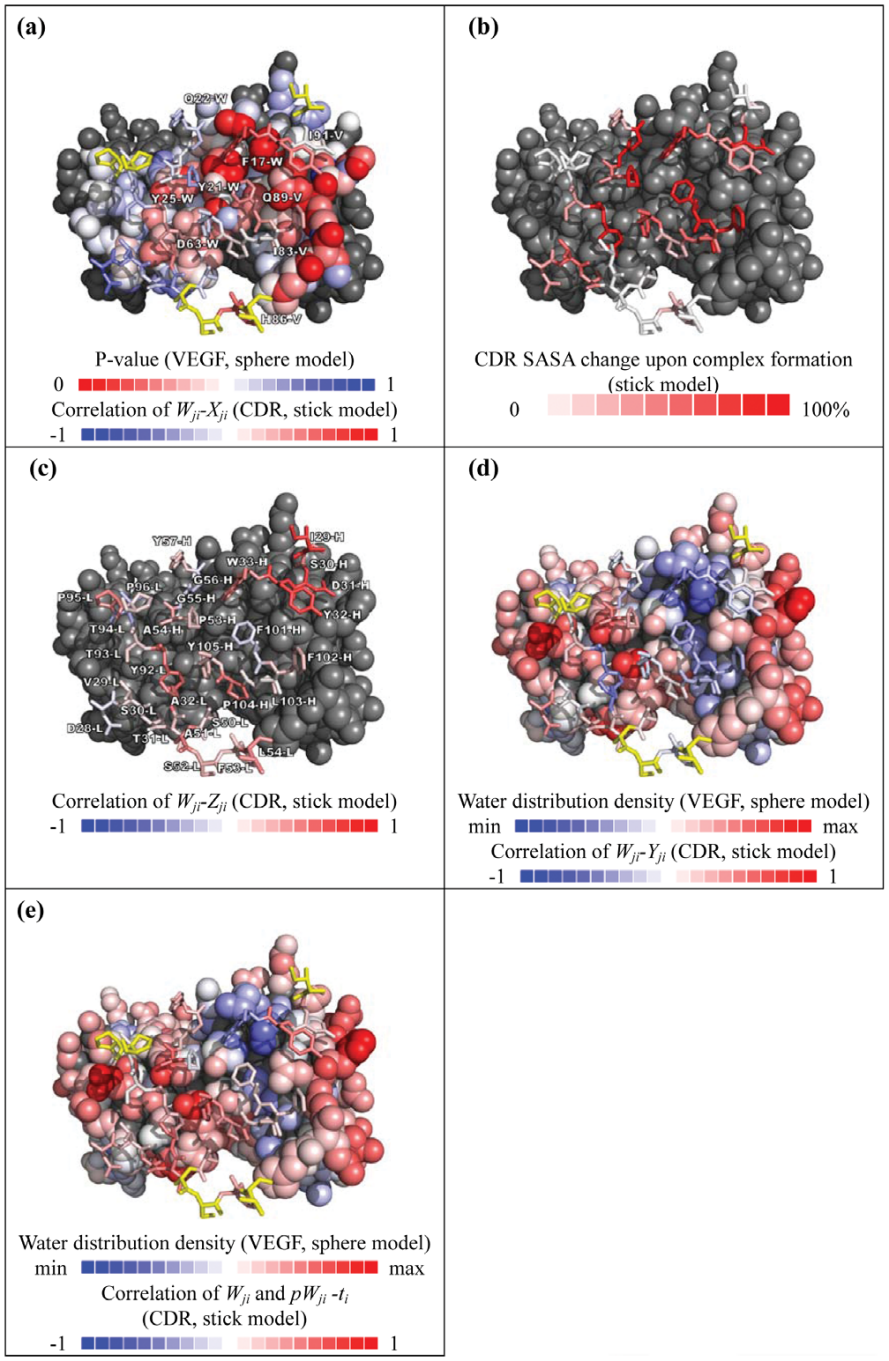
Structure-dependent determinants for the amino acid preferences *W_ji_*. (a) The interface structure of the antibody-VEGF depicted in this panel is attained from the G6-Fab-VEGF complex structure (2FJG). The 30 CDR interface residues are shown in stick model, and the VEGF homodimer structure (V and W chains) is shown in sphere model, where some of the residues are labeled according to the numbering in 2FJG. The CDR interface residues are color-coded based on the *W_ji_-X_ji_* correlation coefficients, for which data for the correlation computation are listed in Table S4. As shown by the bar for color-codes at the bottom of the panel, the residue positions with positive correlation are shown in red and negative correlation are shown in blue. CDR positions that are too distant to make any contact with VEGF are colored in yellow. The VEGF atoms in the interface are colored with increasing redness to highlight the atoms interacting with the antibody variants (method described in Text S1) through increasingly stronger interactions embedded in the *X_ji_* terms, which are derived from the atomistic contact statistics in protein interiors. (b) The 30 CDR interface residues are color-coded in terms of the ratio of the solvent accessible surface area (SASA) change upon the antibody-VEGF complex formation over the solvent assessable surface area in the absence of the VEGF. (c) The CDR interface residues are color-coded according the *W_ji_-Z_ji_* correlation coefficients. (d) The CDR interface residues are color-coded according the *W_ji_-Y_ji_* correlation coefficients. CDR positions that are too distant to render the *Y_ji_* term becoming independent of the amino acid type *i* are colored in yellow. The VEGF atoms are color-coded according to the hydration pattern as shown in Figure 4(a). (e) The CDR interface residues are color-coded according the correlation coefficient of *W_ji_* versus (*pW_ji_- t_i_*) (confidence level of predicted amino acid preference for amino acid *i* at position *j*). The background VEGF atoms are color-coded as in Figure 4(a).

The working hypothesis was tested by calculating the Pearson correlation coefficients (cc) between *W_ji_* and *X_ji_, Y_ji_, Z_ji_* respectively for all amino acid type *i* in each of the position *j*. The results are shown in Table S4 and are summarized in Figure 3. In Figure 3(a), the 30 interface CDR amino acids are shown color-coded based on the *W_ji_-X_ji_* correlation coefficient. The background VEGF interface in Figure 3(a) is also color-coded according to the statistic strength reflecting the average atomistic contact terms calculated for each of the VEGF interface atoms with model scFv structures constructed based on the antigen-binding CDR sequences listed in Table S1 (detailed method described in Text S1). The experimental amino acid preferences *W_ji_* are significantly and positively correlated with the atomistic contact term *X_ji_* in the core interface positions: Y32-H in CDR1H (cc = 0.51), W33-H in CDR1H (cc = 0.67), F101-H in CDR3H (cc = 0.40), F102-H in CDR3H (cc = 0.30), A32-L in CDR1L (cc = 0.42), F53-L in CDR2L (cc = 0.54), Y92-L in CDR3L (cc = 0.34). These positions are consistent with the positions in the core interface region as shown in Figure 1(c) and are located at or near the VEGF interface sub-area colored in red (Figures 1(c) and 3(a)); the color code indicates that this VEGF area is consistently used to make energetically favorable contacts to the binding scFv variants. The energetics governing interactions in this core interface region is closely related to the energetics governing the stability of the interior of protein structures, for which the statistics are used for *X_ji_* calculations. The ranking capabilities of *X_ji_* on the amino acid-protein contact energetics for residues in the core interface are largely comparable to the consensus of 24 publicly available scoring functions devised for computational drug design (Tables S5(a) and S5(b)). This comparison also highlights the diverse ranking results among these 24 scoring functions.

The sub-area color-coded red on VEGF interface in Figure 3(a) is in good agreement with the ‘hot spots’ on VEGF (F17-W, M18-W, Y21-W, Y25-W, Q89-V [26]). Hot spots [29], [30], [31] are frequently buried in the interior of the interface. As shown in Figure 3(b), 5 (Y32-H, W33-H, F101-H, F102-H, and Y92-L) core interface CDR positions are buried in the interface with more than 39% SASA change. In addition, 4 (Y32-H, W33-H, F101-H, and F102-H) out of these 5 buried interface positions are also highly conserved in the sequence patterns (Figure 1). Theses conserved interface residues resemble key residues involving in core structures of tightly packed protein interiors.

Amino acid preferences in the peripheral interface positions are less pronounced but are not indifferent (Figure 1 and Table 1); the sequence preferences of these positions cannot be explained with the structural propensities measured by *Z_ji_* or by the atomistic contact term *X_ji_*. Figure 3(c) shows the interface residue structures color-coded based on the *W_ji_-Z_ji_* correlation coefficients. The amino acid preferences in all the 30 interface positions are correlated with the local structural propensities to various extents, but the correlations do not distinguish the core interface region from the peripheral region in the CDR interface.

Amino acid preferences in the peripheral regions are mostly governed by hydration-mediated interactions, as measured by the *Y_ji_* term. The residues with the most prominent *W_ji_-Y_ji_* correlation are D28-L in CDR1L (cc = 0.40), S30-L in CDR1L (cc = 0.27), S30-H in CDR1H (cc = 0.42), P53-H in CDR2H (cc = 0.31), A54-H in CDR2H (cc = 0.47). All these residues are in the peripheral interface regions. As shown in Figure 3(d), the interface CDR residue structures color-coded based on the *W_ji_-Y_ji_* correlation coefficients are essentially a mirror image to the color-codes shown in Figure 3(a) for the *W_ji_-X_ji_* correlation. This indicates that minimizing the desolvation penalties, in contrast to optimizing the contact energy as in the core interface region, is the major determinant for the amino acid preferences in the peripheral interface CDR positions.

**Figure 4:**
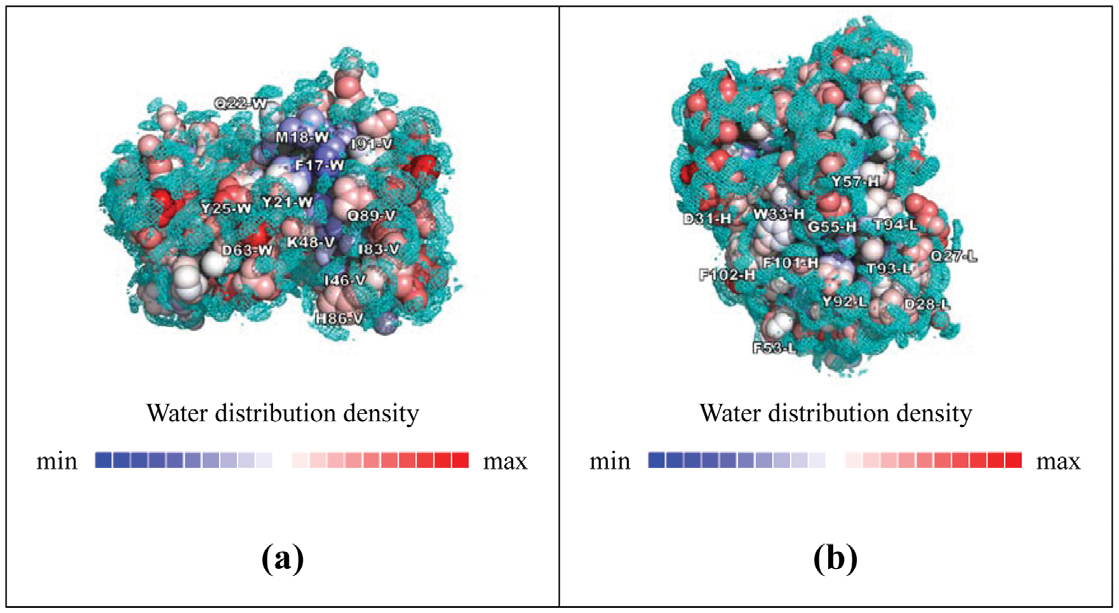
Hydration patterns on the surface of VEGF and the antibody. (a) Hydration patterns of VEGF are shown as the water oxygen atom PDM contours in cyan on the protein surface and as the color-coded atom surfaces. The water PDMs are shown as 0.0019 contours in this panel. The atom surfaces are color-coded according to the hydration pattern near the atom (Equation (3) in Text S1). (b) The hydration pattern for the G6-Fab (2FJG) is shown.

Figure 4 shows the hydration patterns on VEGF and the antibody CDRs. The core interface regions on the VEGF and the corresponding part of the antibody CDRs are much less hydrated than the other surface regions, suggesting that the core interface region in an epitope-paratope pair can be identified with the hydration pattern predictions as shown in Figure 4. Together, the results shown in Figures 3∼4 suggest that the core interface region, composed of only a few less solvated hot-spot residues on the antigen surface, is recognized by a few contact-driven residues on the CDRs of the antibody, forming the core of the antibody-antigen recognition interface without substantial energetic penalty due to the dehydration of the core interface. The energetics for this core interface assembly resembles that governing the stability of the interior of protein structures. The CDR positions surrounding the core interface prefer small hydrophilic sidechains over the possibility of forming specific inter-protein van der Waals interactions and hydrophobic contacts. Hydration-mediated interactions indirectly linking the hydrophilic groups in both sides of the protein interface surrounding the core interface region provide non-specific but weak adhesive driving force for the interface, explaining the non-specific preferences for small and hydrophilic amino acid sidechains in these positions. Bulky hydrophobic sidechains that abolish this weak interaction and introduce larger dehydration penalties are less preferable in the peripheral interface region.

Although protein-protein core interfaces anchored by hot spots [29], [30], [31] and the hydration-mediated interactions in the peripheral protein-protein interface regions [18], [21], [22], [23], [24] have been well-established qualitatively, the results shown in Figure 1 nevertheless provide rich information on the amino acid preferences for the positions in each of the interface regions. More importantly, the new information led to insights at atomic resolution for quantitative evaluations of the amino acid preferences in the interface positions (Figure 3). Computational algorithms based on these quantitative insights should be useful in designing antibody CDR sequences targeting at a designated epitope of known structure (see below).

### Machine learning models for computational antibody desig

The results above suggest that the *X_ji_, Y_ji_, Z_ji_* terms should be useful in predicting the amino acid preferences in the CDR interface. We chose the minimal logistic regression model with weighted sum of the *X_ji_ Y_ji_* and *Z_ji_* terms as input (Equation (4) in Methods) to predict the amino acid preferences at each of the interface CDR positions contacting the antigen (24 positions, not including the 6 non-contact positions as colored in yellow in Figure 3(a) and (d)). The rationale is that the *X_ji_, Y_ji_, Z_ji_* terms should carry enough information to evaluate as to what extent an amino acid type *i* is suitable for an interface environment surrounding the position *j*. The machine learning was carried out by training one logistic regression model for each of the 20 amino acid types at each of the 24 CDR positions; the amino acid preferences at each of the interface positions were predicted with the 20 logistic regression models trained with information from all other 23 positions and the binary predictions (δ*pW_ji_* in Equation (4)) were assessed with the positives and negatives determined experimentally for the position (leave-one-out cross-validation so that the training set does not include the test case). The weights of the trained models and the prediction results are shown in Table S4.

The logistic regression model was chosen because of the simplicity of the weighted linear combination of the *X_ji_ Y_ji_* and *Z_ji_* terms as input. The linear combination requires only small number of parameters (each model requires only 5 variable parameters; see Equation (4) in Methods). In contrast, in more sophisticate machine learning models, such as artificial neural network or support vector machines, each model would frequently require tens to hundreds of weight parameters. These machine learning models are not suitable for the application in this work because the number of available data points is relatively too small for the machine learning models. The regression algorithm using 23 data points to optimize 5 variable parameters is chosen so as to avoid over-fitting of the machine learning models.

The effectiveness of the amino acid preference prediction by the logistic regression models is summarized in Table 1. In practice, positive *W_ji_* (observed count for amino acid *i* at position *j* is greater than the anticipated frequency of amino acid *i* encoded in the degenerate codon NNK) for amino acid type *i* is considered as positive (δ*W_ji_* = 1) in position *j*, and the predicted positive amino acid types (δ*pW_ji_* = 1) have the output activation value (*pW_ji_*) from the logistic regression models greater than the threshold *t_i_* (Equation (4)). The overall Matthews correlation coefficient (MCC, Equation (10)) for the amino acid preference binary predictions (leave-one-out cross validation) of the 20 amino acid types in the 24 interface CDR positions is 0.312, calculated with the experimental and prediction data shown in Table 1. The MCC for random predictions would be zero and perfect predictions would yield MCC of one. On average, each interface position has 5.7±2.0 positive amino acid types; 7.7±2.0 amino acid types are predicted positive on average at each position and 3.5±1.9 amino acid types are true positive on average at each position – the accuracy, precision, recall (sensitivity) and specificity (Equations (6)∼(9) in Methods) of the overall binary predictions are 0.69, 0.45, 0.63, and 0.71 respectively. In comparison, a random prediction of 5.7 residues for each of the 24 positions would yield 0.59, 0.29, 0.29, and 0.72 for the same set of prediction effectiveness measurements.

The prediction accuracies (*W_ji_*−(*pW_ji_−t_i_*) correlation coefficients) shown in Figure 3(e) are inversely correlated (cc = −0.40) with the SASA change shown in Figure 3(b) and are positively correlated (cc = 0.25) with *W_ji_-Y_ji_* correlation coefficient (Figure 3(d)). This suggests that, in general, the *y_ji_Y_ji_* term in Equation (4) gives more weight in determining *pW_ji_* and thus the sequence preference predictions are more accurate for the peripheral interface positions.

The machine learning models above enabled a formal statistical extrapolation with accuracy to an extent, providing suggestions on optimal CDR sequences capable of binding to a designated epitope based on rules learned from a limited set of data. The phage display data have provided invaluable but nevertheless incomplete picture on the possible combinations of CDR residues in recognizing the epitope on the antigen. Even the limited sequence space of the CDR variants selected for binding to the same epitope on VEGF could not be completely explored with only several hundreds of selected and confirmed binders as shown in Figure 1; many of the amino acid combinations that have not been observed from the limited sampling remain uncertain. The logistic regression models above have enabled a better use of the limited dataset in predicting optimal CDR sequences unseen previously in an antibody-antigen interface.

### Further tests of the prediction of optimal CDR residues in other antibody-antigen interfaces

**Figure 5:**
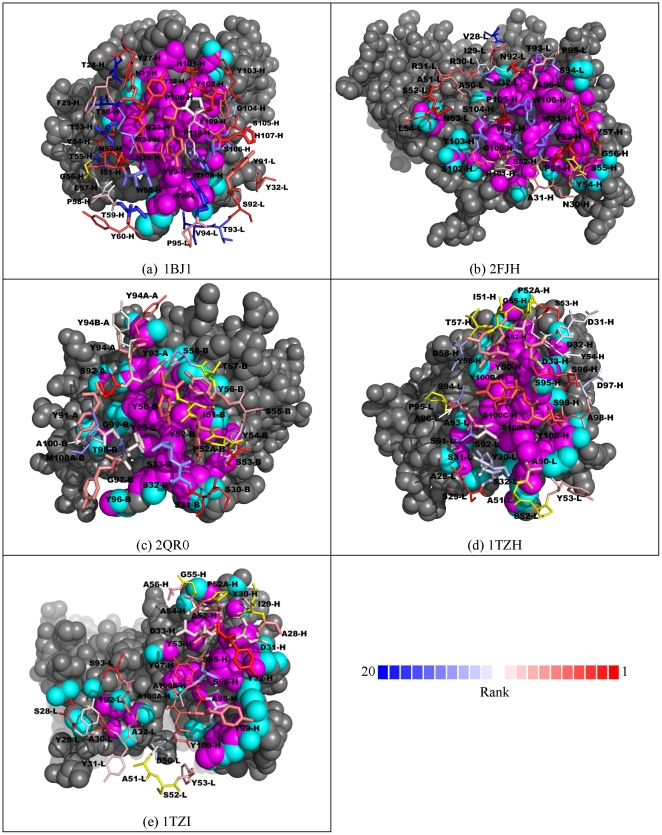
Predicted ranking of the CDR amino acids in the antibody-VEGF complex interfaces. Panel (a) to (e) shows the complex structure for 1BJ1, 2FJH, 2QR0, 1TZH, and 1TZI respectively. In each of the panels, the atoms of the antigen VEGF are shown in spheres; the VEGF atoms colored in magenta are core interface atoms and the VEGF atoms colored in cyan are rim interface atoms. The core-rim assignment follows the definition previously published [54]. The CDR atoms in the core interface are shown in thick stick model and the CDR atoms in the rim interface are shown in thin stick model. The ranking of the CDR amino acids in the structures are color-coded according to the color bar shown at the bottom of the figure: Better ranking is shown with increasing depth in red; worse ranking is shown with increasing depth in blue. The CDR residues colored in yellow are not in contact with the antigen.

The capabilities of the machine learning models were further tested with other antibody-VEGF complex interfaces, where the anti-VEGF antibody sequences are different with different corresponding epitope on the antigen. Two of these complex interfaces (1BJ1 [32] and 2FJH [26]) have been optimized for high affinity, while the other three anti-VEGF antibodies (2QR0 [33], 1TZH and 1TZI [34]) were selected from phage display libraries with only limited amino acid variations (Y, S, D, A) in selected CDR residues. Since the corresponding phage display variant profiles as shown in Figure 1 are not available from the associated studies, similar analyses shown in Figure 3 could not be carried out. Alternatively, we predicted the ranking of the 20 natural amino acid types in each of the CDR positions defined in the respective antibody-antigen complex structures and highlight the rank of the CDR amino acids in the crystal structures based on the ranking predicted with the trained logistic regression models (Figure 5). It is not known from experimental data as to if there exist other more optimal amino acid types in comparison with the corresponding amino acid type in the structure, but nevertheless, we assume that amino acids in the structures with better ranks are predicted more accurately. Figure 5 depicts the summary of the predictions, and the details of the prediction results are shown in Table S9.

As shown in Figures 5(a) and 5(b) for complex 1BJ1 and 2FJH respectively, the Tyr residues in the core interface were predicted with high accuracy, while the core Trp residues were predicted incorrectly. It is of interest to compare the results with those of the three antibody-antigen interfaces where the antibodies are members of minimalist antibody libraries with limited amino acid variations (Y, S, D, A) in the CDR residues (Figures 5(c), 5(d), 5(e) for complex 2QR0, 1TZH, 1TZI respectively). Among the residues in these core interfaces, only one Tyr residue was poorly predicted in the complex 1TZH. It is evident that the minimalist antibody library designs have not only substantially reduced the complexity space of the CDR regions, the computational antibody design herein can be better applied to a system with less complex combinatorial selections.

**Figure 6:**
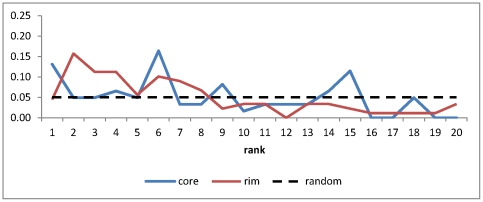
Distributions of the predicted ranking of the CDR amino acids in the antibody-VEGF complex interfaces. The x-axis shows the predicted ranking of the CDR amino acids in the five antibody-VEGF complex interfaces as shown in Figure 5. Detailed prediction results are shown in Table S9. The y-axis shows the percentage of the CDR amino acids predicted with the ranking shown in x-axis. The red line shows the distribution for the amino acids in the core interface, while the blue line shows the distribution for the amino acids in the rim interface. These two distributions are compared with random distribution shown as the flat dash line in the figure.

Overall, the sequence preferences in the peripheral regions of the antibody-antigen interfaces were better predicted than the core regions. Figure 6 summarizes the distribution of the predicted ranking of the amino acids in the complex structures. The amino acids in the peripheral interface regions are generally predicted with better ranking (distribution in red in Figure 6) in comparison with the predictions for the amino acids in the core interface regions (distribution in blue in Figure 6). Both distributions of predicted ranking are better than random distribution shown as the flat dashed line in the figure. These are in agreement with the benchmark results shown above in Figure 3.

### Computational recombinant antibody library design

The prediction algorithm can be used to design antibodies against epitopes of known structure. The peripheral interface sequence preferences can be predicted to an extent with the trained machine learning models above. The sequence preference predictions for these regions are more accurate and these positions are more tolerant in amino acid preference prediction errors (Figure 1 and 3). The core interface CDR sequences can be better predicted with the *X_ji_* term alone (Figure 3(a)). However, these CDR core interface designs need to be validated with focused experiments because of the high stringency of sequence preferences in this area. To this end, the core interface CDR residues would be encoded with degenerate codons biased towards aromatic and hydrophobic amino acid types so as to form protein interior-like interactions with the hot-spot residues on the antigen. As such, the designed synthetic DNA libraries are confined to the experimental limit of about one billion variants for phage display because the number of the core interface residues encoded by degenerate codons would be likely less than 10.

The results above suggest a methodology for computational design of synthetic antibody libraries for high-throughput antibody discovery platforms. First, the antibody binding site (epitope) on the antigen containing hot-spot residues with sparse hydration patterns (methodology as shown in Figure 4 and Text S1) is defined. Models of the antibody-antigen complex structures with only mainchain structure for the CDR interface residues targeting the selected epitopes are constructed with computational molecular modeling and docking of the antibody and antigen structures. The CDR interface residues on the antibody near the antigen hot-spot residues are the core interface residues; the rest of the CDR residues are peripheral interface residues. The amino acid types and rotameric conformations are enumerated at each of the CDR positions to predict the amino acid preferences for the peripheral and the core CDR positions with the machine learning models. Antibody libraries designed based on the model complex structures, the peripheral sequence preferences, and the core interface residue libraries can then be selected and screened with the standard high throughput phage display platform, which has been used in attaining results shown in Figure 1. The antibody library design methodology will further mature with extended experimental verification, for which the experimental platforms have been well-established [5]. The structure-based *in silico* design of recombinant antibody libraries will provide alternatives to the current animal-based antibody technologies to facilitate the discoveries of antibody therapeutics and diagnostics and to enrich the basic understanding of protein-protein interactions in general.

## Materials and Methods

### Phage display of VEGF-binding scFv and sc-dsFv

The methods for preparing anti-VEGF template scFv, scFv phagemid, sc-dsFv phagemids, anti-VEGF scFv/sc-dsFv phages and for scFv/sc-dsFv panning, single colony and ELISA analysis, and interdomain disulfide bond formation analysis have been described previously [27], [28].

### Expression and purification of sc-dsFv

The expression and purification of the sc-dsFv followed the method described previously with minor modifications [35]. In brief, the sc-dsFv coding region was subcloned into pET-32 expression vector encoding thioredoxin as a fusion protein N-terminal to the sc-dsFv. The fusion protein contains a hexa-His tag followed by a TEV protease cutting site between the thioredoxin and the sc-dsFv, which is followed by an Avitag oligopeptide (GLNDIFEAQKIEWHE, Avidity Inc., USA) appending to the C-terminus of the sc-dsFv for *in vivo* biotynylation. The sc-dsFv gene derived from phage panning was subcloned into the expression vector via the *Sfi*I and *Not*I cutting sites encompassing the sc-dsFv coding region. *E. coli* Rosetta-gami B (DE3) strain culture transformed with scFv/sc-dsFv expression vector was grown in 2× YT medium (Tryptone 16 g/L, Yeast extract 10 g/L, NaCl 5 g/L) with ampicillin (200 µg/L), tetracycline (12.5 µg/L) and chloramphenicol (37.5 µg/L) at 30°C until OD_600_ reached 1.0, and was then incubated at 20°C for another 2 hr before adding 0.2 mM IPTG. After overnight protein expression and centrifugation, the cell pellets were resuspended in lysis buffer (Tris-HCl, 50 mM, pH 8.0, 150 mM NaCl, 30 mM imidazole) and the suspended cells were then broken by Microfluidizer (Microfluidics, MA). The recombinant thioredoxin-sc-dsFv fusion protein was purified by nickel chelation chromatography with IMAC prepacked column (GE Healthcare Life Sciences) charged by 0.1 M NiSO_4_ solution. The fractions containing the fusion protein were collected and dialyzed by Tris-HCl, 50 mM, pH 7.5 (the theoretical pI of sc-dsFv was 5.82) overnight at 4°C or desalted by HiPrep 26/10 desalting column (GE Healthcare Life Sciences) with the same buffer. The protein solution was then introduced to ion-exchanged chromatography (prepacked Q column, GE Life Healthcare Sciences). The fractions containing the thioredoxin-sc-dsFv fusion protein were collected and treated with His_6_-tagged TEV protease (A_280_ ratio 50∶1) at 30°C for at least 5 hr but not longer than 8 hr. The TEV-cleaved fragment containing His_6_-tagged thioredoxin and the His_6_-tagged TEV protease were removed by nickel chelation chromatography. The fractions containing sc-dsFv were further purified with a Superdex75 size-exclusion column (GE Healthcare Life Sciences) in SEC buffer (Tris-HCl, 50 mM, pH 7.5, 400 mM NaCl, 10% glycerol). The soluble sc-dsFv protein was prepared with 95% purity. The purified sc-dsFv was stored at 4°C for a least one week without affinity loss.

### Crystallization of sc-dsFv

The sc-dsFv solution was concentrated to 10 mg/ml without precipitation. Crystallization screening after PCT test (Hampton Research) for the sc-dsFv was carried out with the protein in 6 mg/ml concentration. The crystallization screening of sc-dsFv was carried out in Mosquito® (TTP LabTech Ltd., United Kingdom) with screening kits from Hampton Research (Laguna Niguel, CA) and Molecular Dimension (Apopka, FL). The purified sc-dsFv in SEC buffer was mixed with an equal volume of the reservoir solution (100 mM Tris–HCl, 100 mM MgCl_2_ and 20% PEG4000, pH 8.0) and then crystallized at 20°C by the hanging-drop vapor-diffusion method. The crystals appeared after one day incubation at 20°C, and reached 0.2 mm×0.1 mm×0.1 mm in dimension in one week. A 2.4-Å X-ray diffraction data set was collected at the beamline 13C1 of the National Synchrotron Radiation Research Center (Hsinchu, Taiwan). Before the crystal was mounted on the X-ray machine, the crystal was soaked briefly in reservoir solutions containing 20% (v/v) glycerol as a cryoprotectant. All diffraction data were indexed, integrated, and scaled with HKL2000 package [36].

### Structure determination and refinement

The crystal structure of sc-dsFv was solved by the molecular replacement method The refinement procedure used one pair of the variable fragment structure derived from the published Fab structure (2FJF in PDB code [26]) as the search model and using the software molrep [37] in CCP4 package [38]. Only one clear solution was found and the R-work and R-free values of the initial 30 rounds of Refmac5 refinement [39] were 0.2581 and 0.3003, respectively. The resolved structures contained six sc-dsFv molecules in one asymmetric unit. The residues that were different between sc-dsFv and variable fragments derived from 2FJF were replaced automatically by MrBUMP module (Automated Model generation and Molecular Replacement) in the CCP4 package. The manual structural adjustment and well-ordered water molecule placement were carried out with Coot software [40]. Iterative cycles of computational refinements were performed by phenix-refine [41] with TLS options turned on. The TLS groups were determined by the TLSMD server [42]. The progress of the refinements was monitored by both of the R-work and R-free values. The stereochemical quality of the refined structures was checked by PROCHECK [43] within Structural Analysis and Verification Server (NIH MBI Laboratory for Structural Genomics and Proteomics, UCLA). The RMSD between each sc-dsFv molecule in asymmetry unit, among the whole molecules or CDRs between sc-dsFv and variable fragments derived from 2FJF or 2FJG [26] were calculated by PyMOL (The PyMOL Molecular Graphics System, DeLano Scientific, San Carlos, CA, USA. http://www.pymol.org). The sc-dsFv structure coordinates and refinement data have been submitted to PDB under the code 3AUV.

### Computation of W_ji_, X_ji_, Y_ji_, Z_ji_ and pW_ji_

The model antibody structures were identical to the template structure (derived from 2FJG in PDB) except that the 30 interface CDR residues were all replaced with alanine to mimic realistic situations where CDR sequences were not known. To build the sidechain of the residue at position *j*, the amino acid type *i* adopting a rotameric structure *k* from the penultimate rotamer library [44] was locally optimized with the “Clear Geometry” function in Discovery Studio (version 2.5, Accelrys) while the rest of the antibody-antigen complex remained fixed. The sidechain conformations clashing with the rest of the protein complex structure were removed from further consideration.

For each of the model structures, the atomistic contact component *X_ji_* of the scoring system was calculated with the probability density maps (PDMs) describing the distribution of amino acid atoms on the surface of the antigen based on atomistic contact statistics observed in protein interiors. The PDMs were constructed following the basic idea first developed by Laskowski et al [45] with substantial modifications to largely eliminate the distortion of the predicted PDMs due to distributions of the amino acid sidechain and mainchain dihedral angles (examples of atomistic density distributions can be viewed via internet – http://ismblab.genomics.sinica.edu.tw/> Introduction). Briefly, the amino acid conformations in proteins were classified according the conformation clusters (Table S6); the PDMs for the protein atom types (Table S7) were constructed in an amino acid conformation-dependent manner, while non-interacting atom pairs were eliminated from the PDMs based on a statistic pairwise atomistic interaction preference filter (Table S8). The detailed method for calculating the PDMs is described in full in Text S1. Figure S1 depicts the flow chart of the computational procedure for PDMs. The computational tools are available from the ISMBLab (In Silico Molecular Biology Laboratory) web server: http://ismblab.genomics.sinica.edu.tw/.

The atomistic contact term *X_ji_(rotamer_k_)* for amino acid *i* adopting rotameric conformation *k* at position *j* in the CDR of the antibody was calculated based on a model antibody-VEGF complex structure described above. Equation (1) shows the calculation of *X_ji_(rotamer_k_)*:
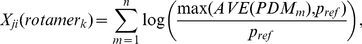
(1)where amino acid *i* has *n* atoms. *AVE(PDM_m_)* is the averaged PDM value corresponding to atom *m* on the surface of the antigen. This value was calculated by summing the PDM values corresponding to the atom *m* at the grids enclosed in the van der Waals volume of the atom *m* and the sum was then divided by the number of grid points enclosed in the atom to yield *AVE(PDM_m_)*. *p_ref_* in Equation (1) is the reference probability for an atom at a reference point far apart from the antigen surface. The reference probability must be smaller than the minimal PDM value (∼10^−10^ in this work) but cannot equal to zero, so as to avoid singularity in calculating *X_ji_(rotamer_k_)* when *AVE(PDM_m_)* equals to zero. No experimental data can be used to derive the reference probability, but we found that when *p_ref_* is smaller than 10^−10^, the relative ranking of the *X_ji_(rotamer_k_)* term is independent to the selection of the reference probability. Even when the value of the *p_ref_* is set between 10^−8^ and 10^−10^, the correlation of the *X_ji_* term with the experimental amino acid preference *W_ji_* does not change. Thus *p_ref_* = 10^−10^ has been empirically determined in this work; the correlating the *X_ji_* term with the experimental amino acid preference *W_ji_*, as shown in Table S4, is insensitive to the selection of the *p_ref_* value.

The hydration-mediated term *Y_ji_(rotamer_k_)* for amino acid *i* adopting rotamer conformation *k* at position *j* in the antibody is calculated based on the model antibody-antigen complex structure with the following equation:
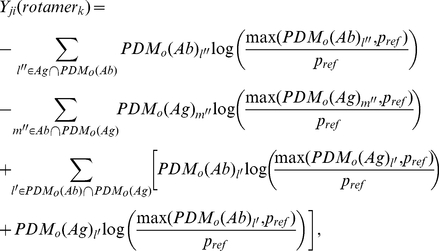
(2)


where *l″* is the grid index for the grid points inside the antigen molecular volume and *PDM_o_(Ab)_l″_* is the water oxygen PDM value at grid point *l″*; *m″* is the grid index for the grid points inside the antibody molecular volume and *PDM_o_(Ag)_m″_* is the water oxygen PDM value at grid point *m″*; *l′* is the grid index for the grid points within the overlapping volume between *PDM_o_(Ab)* and *PDM_o_(Ag)*. The *PDM_o_(Ab)* is the water oxygen PDM on the antibody surface in the absence of the antigen. The *PDM_o_(Ab)* was calculated for the amino acid *i* only. The *PDM_o_(Ag)* is the water oxygen PDM on the antigen surface in the absence of the binding antibody. Following the same rationale as in Equation (1), the reference probability *p_ref_* is set at 10^−10^. Again, the value of *p_ref_* does not affect the ranking order of *Y_ji_* calculated for each amino acid type and rotamer conformation. The first two negative terms (the terms involving *l″* and *m″*) in the right-hand-side of the equation is the desolvation terms for removing water from the binding site of the antibody and the antigen respectively; the positive terms (the terms involving *l′*) correspond to the water-mediated interactions – the first positive term accounts for the interactions between the waters on the surface of the antibody and the atoms on the surface of the antigen, and the second positive terms accounts for the interactions between the waters on the surface of the antigen and the atoms on the surface of the antibody. The first two negative desolvation terms are against binding of the antibody and antigen, while the two positive water-mediated interactions terms are favorable for the antibody-antigen interactions (hydration pattern prediction request to http://ismblab.genomics.sinica.edu.tw/> predict > protein hydration pattern).

The calculation of the structural propensity *Z_ji_* for amino acid *i* in position *j* has been described in details in a previous paper [46]. Briefly, local structures in PDB similar to the CDR structures flanking with two stem β-structures in the unbound antibody structure (2FJF) were collected with PrISM using the threshold of PSD<0.1[47]; these local structures were multiple-aligned based on structural similarity with PrISM [46], [47], [48], [49], [50]. *Z_ji_* was calculated with the structure-based multiple sequence alignments as the following [46]:
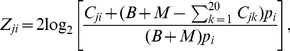
(3)where *C_ji_* is the number of the amino acid *i* that appears in the position *j* of the multiple sequence alignment; *p_i_* is the background probability for amino acid *i* in proteins; *M* is the number of rows in the sequence profile; the term *(B+M-Σ_k = 1,20_C_jk_)* is the Bayesian prediction pseudo-count, where *B = M^0.5^* is adequate in the calculation.

Predicted amino acid preference in binary form δ*pW_ji_* for amino acid type *i* at position *j* was determined by the logistic regression model:
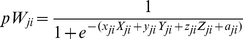
(4)δ*pW_ji_* = 1 when *pW_ji_≥t_i_*; otherwise δ*pW_ji_* = 0, where
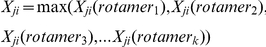


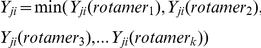
The *X_ji_* in Equation (4) is the upper bound of the atomistic contact term for amino acid *i* at position *j*; *X_ji_* for each of the rotamer model structures *1∼k (here, k is the total number of rotamers for amino acid type i)* for amino acid *i* at position *j* was calculated (Equation (1)) and the largest *X_ji_* in this set of *X_ji_(rotamer_1∼k_)* was used in Equation (4). This term corresponds to the most favourable contribution from amino acid *i* at position *j* to the protein-protein complex formation. *Y_ji_(rotamer_1∼k_)* for each of the rotamer model structures *1∼k* of amino acid *i* at position *j* was calculated (Equation (2)) and the smallest *Y_ji_* in this set of *Y_ji_(rotamer_1∼k_)* was used in Equation (4). This *Y_ji_* reflects the maximal desolvation energy penalty due to the amino acid *i* at position *j* in forming the protein-protein complex; the rotamer conformation with the maximal desolvation energy penalty is the most favourable conformation in the hydration environment before forming the antibody-antigen complex.

At each position *j*, one logistic regression model was trained for each of the 20 amino acid types. The weights (*x_ji_,y_ji_,z_ji_,a_ji_*) in Equation (4) for predicting the preference of amino acid type *i* at position *j* were optimized with a logistic regression algorithm in MATLAB to minimize the difference between *pW_ki_* in Equation (4) with *W_ki_* from Equation (5) (see below), where *k* represents all the positions except for the position *j.* The optimized weights (*x_ji_,y_ji_,z_ji_,a_ji_*) were then applied to Equation (4) to predict *pW_ji_* for amino acid type *i* at position *j* (i.e., leave-one-out cross validation approach to mimic the prediction situation). The leave-one-out training process was carried out through all the 24 positions in this work. The thresholds *t_i_* for the 20 amino acid types were optimized in the training process to maximize the Matthews correlation coefficient (MCC, Equation (10)) for the leave-one-out cross validation predictions.

The experimental amino acid preferences *W_ji_*, as shown in Table 1 and in Table S4 are expressed in half-bit units calculated with the Bayesian prediction pseudo-count method [46], [51]:
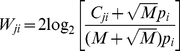
(5)
*δW_ji_* = 1 when *W_ji_*≥0; otherwise *δW_ji_* = 0, where *W_ji_* is the preference for amino acid *i* at position *j* in the CDR of the antibody; *C_ji_* is the count for amino acid *i* at position *j*; *M* is the count of VEGF-binding CDR sequences containing position *j*; *p_i_* is the background probability for amino acid *i* encoded in the NNK degenerate codon [51]; the square root of M in the equation is the pseudo count to prevent singularity when *C_ij_* equals to zero. In practice, positive *W_ji_* (observed count for amino acid *i* at position *j* is greater than the anticipated frequency of amino acid *i* encoded in the degenerate codon NNK) for amino acid type *i* is considered as positive in position *j* (*δW_ji_* = 1 when *W_ji_*≥0).

The predictions were assessed by comparing the positives (*δW_ji_* = 1, see Equation(5)) and negatives (*δW_ji_* = 0, see Equation(5)) with the predicted positives (*δpW_ji_* = 1, see Equation (4) and the predicted negatives (*δpW_ji_* = 0, see Equation(4)). The predicted positives are composed of true positives (TP) and false positives (FP), while the predicted negatives are composed of true negatives (TN) and false negatives (FN). The accuracy, specificity, recall (sensitivity), precision and Matthews correlation coefficient (MCC) [52] of the binary predicted results are expressed as below:
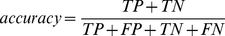
(6)

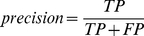
(7)

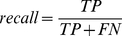
(8)

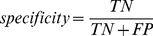
(9)

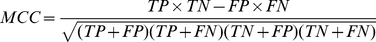
(10)


## Supporting Information

Figure S1 **Flow chart depicting the procedure in constructing probability density maps (PDMs) for non-covalent atomistic interactions on protein surfaces.**
(DOC)

Table S1 **Sequence information and VEGF-binding and expression data for the scFv variants shown in** **Figure 1** **.**
(DOC)

Table S2 **Sequence information and VEGF-binding and expression data for the sc-dsFv variants shown in** **Figure 1** **.**
(DOC)

Table S3 **Data collection and refinement statistics for the sc-dsFv x-ray crystallography.**
(DOC)

Table S4 **Leave-one-out cross validation predictions for the amino acid preferences at each of the 30 CDR interface residues.**
(DOC)

Table S5 **Ranking antibody CDR single site amino acid binding to VEGF with public domain scoring functions. Table S5b, the top-ranked amino acid types and rotamers with various scoring systems.**
(DOC)

Table S6 **Amino acid conformation classifications.**
(DOC)

Table S7 **Atom types in protein structures.**
(DOC)

Table S8 **Statistic pairwise atomistic interaction preferences.**
(DOC)

Table S9 **The predicted ranking of the 20 natural amino acid types at each of the CDR amino acid positions in the 5 antibody-VEGF complex structures.**
(DOC)

Text S1 **Supplemental Methods.**
(DOC)
