# Supplementary material for: Rationalization and Design of the Complementarity Determining Region Sequences in an Antibody-Antigen Recognition Interface
Source: PLoS One. 2012 Mar 22;7(3):e33340. doi: 10.1371/journal.pone.0033340 (PMC3310866; doi:10.1371/journal.pone.0033340)
Supplement: Figure S1 — Flow chart depicting the procedure in constructing probability density maps (PDMs) for non-covalent atomistic interactions on protein surfaces. (DOC) [file pone.0033340.s001.doc]

**Figure S1**


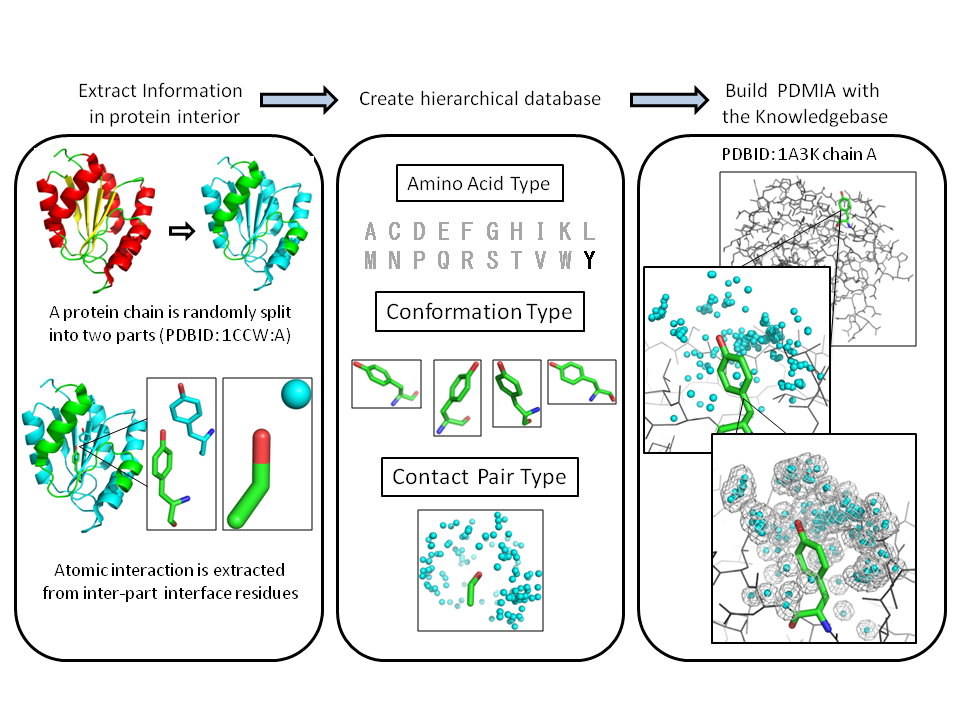


**Figure S1**. Flow chart depicting the procedure in constructing probability density maps (PDMs) for non-covalent atomistic interactions on protein surfaces. The details of the computational methodology are described in the Supplementary Methods section.
