# Supplementary material for: Rationalization and Design of the Complementarity Determining Region Sequences in an Antibody-Antigen Recognition Interface
Source: PLoS One. 2012 Mar 22;7(3):e33340. doi: 10.1371/journal.pone.0033340 (PMC3310866; doi:10.1371/journal.pone.0033340)
Supplement: Table S1 — Sequence information and VEGF-binding and expression data for the scFv variants shown in Figure 1 . (DOC) [file pone.0033340.s002.doc]

**Table S1**. Sequence information and VEGF-binding and expression data for the scFv variants shown in Figure 1. All the measurements shown in this Table were carried out in three repeats. The experimental details can be found in previous publications .

Table S1 (A): data derived from L3H3 scFv phage display library.

| L3H3 | |  |  | VEGF | | Anti-E | | VEGF/anti-E ratio to AV1 (-TAA) | fXa/-fXa (-TAA)(%) |
| --- | --- | --- | --- | --- | --- | --- | --- | --- | --- |
|  | Sequencing ID | L3 | H3 | average | stdev | average | stdev |  |  |
|  | AV1 | YTTPP | FFLPY | 1.000 | 0.026 | 1.000 | 0.054 | 1.000 | 17.18 |
| 1 | RV25D | FKGAF | FFRGN | 9.667 | 0.141 | 1.725 | 0.163 | 5.603 | 61.97 |
| 2 | 1-0919-1C-2 | FKGAP | FFLPY | 16.400 | 0.163 | 1.069 | 0.010 | 15.346 | 62.72 |
| 3 | 1-0910-0925-1G | FKPAH | LFLRA | 3.767 | 0.038 | 0.893 | 0.081 | 4.219 | 36.68 |
| 4 | 1-0910-4H | FKRAH | HFLPG | 7.267 | 0.101 | 1.318 | 0.020 | 5.515 | 28.77 |
| 5 | 1-0910-9F | FKSGH | FHLQN | 2.883 | 0.031 | 0.944 | 0.077 | 3.054 | 6.29 |
| 6 | 1-0910-0925-9F | FRVPM | FYLQG | 16.767 | 0.305 | 0.708 | 0.058 | 23.677 | 76.26 |
| 7 | 1-0910-9E | FSRGA | WYRYG | 1.183 | 0.019 | 1.219 | 0.051 | 0.971 | 8.76 |
| 8 | 1-0910-10C | HKPAH | HYLSS | 9.483 | 0.097 | 2.030 | 0.069 | 4.671 | 44.61 |
| 9 | RV21H | HMRSH | MFARG | 2.600 | 0.035 | 1.996 | 0.189 | 1.303 | 8.61 |
| 10 | 1-0910-0925-11E | HNRTG | MFLSG | 0.367 | 0.004 | 0.730 | 0.047 | 0.503 | 7.99 |
| 11 | 1-0910-0925-9G | HPTFF | GPWFP | 0.150 | 0.003 | 1.073 | 0.039 | 0.140 | 3.85 |
| 12 | 1-0910-0925-8E | HSARE | FFLDR | 4.167 | 0.032 | 1.502 | 0.156 | 2.774 | 18.40 |
| 13 | 1-0910-0925-12F | IKAPL | FMRHG | 2.983 | 0.013 | 0.854 | 0.056 | 3.493 | 16.55 |
| 14 | 1-0910-10D-4 | IKQLA | WYRGG | 6.833 | 0.061 | 1.438 | 0.050 | 4.753 | 45.56 |
| 15 | 1-0910-3B | IMKFE | FFLGA | 0.100 | 0.003 | 0.888 | 0.033 | 0.113 | 15.83 |
| 16 | RV21A | ITDPL | FYLQG | 15.667 | 0.096 | 1.910 | 0.024 | 8.203 | 54.89 |
| 17 | 1-0910-0925-10E | LIRKS | WFLGM | 0.350 | 0.007 | 0.944 | 0.034 | 0.371 | 16.84 |
| 18 | 1-0910-0925-10C | LIRTP | WYLHG | 11.067 | 0.116 | 1.416 | 0.111 | 7.814 | 51.17 |
| 19 | 1-0910-0925-12C | LMPPH | LYTQG | 0.267 | 0.005 | 0.858 | 0.043 | 0.311 | 11.01 |
| 20 | 1-0910-3F | LNPGY | FFLFN | 2.283 | 0.039 | 0.601 | 0.034 | 3.800 | 37.27 |
| 21 | 1-0910-0925-11A | LNPLN | FFLRA | 2.250 | 0.029 | 0.850 | 0.025 | 2.648 | 32.30 |
| 22 | 1-0910-2E | LSGSH | FSLLG | 16.967 | 0.264 | 1.803 | 0.022 | 9.412 | 34.27 |
| 23 | 1-0910-0925-9E | LSTVH | WTLSG | 1.067 | 0.013 | 1.837 | 0.042 | 0.581 | 4.70 |
| 24 | 1-0910-0925-2A | LTSLS | WYLRS | 1.283 | 0.022 | 1.288 | 0.063 | 0.997 | 34.31 |
| 25 | 1-0910-0925-11D | NKNLF | HYLHG | 1.867 | 0.028 | 1.300 | 0.120 | 1.435 | 15.46 |
| 26 | 1-0910-5G | NMPSN | WFLRQ | 2.800 | 0.048 | 0.957 | 0.095 | 2.926 | 41.21 |
| 27 | 1-0910-9G | NNSPH | HYLSG | 16.733 | 0.121 | 1.983 | 0.212 | 8.439 | 58.13 |
| 28 | 1-0910-0925-12D | NRSKY | FYLNQ | 2.033 | 0.016 | 1.013 | 0.071 | 2.007 | 13.79 |
| 29 | 1-0913-2G | NRTRM | FYLNY | 0.567 | 0.008 | 0.524 | 0.022 | 1.082 | 4.42 |
| 30 | 1-0910-0925-8B | RNPND | HFLFS | 0.850 | 0.015 | 1.592 | 0.046 | 0.534 | 16.20 |
| 31 | 1-0910-6F | RSISR | FYLSS | 0.300 | 0.003 | 1.528 | 0.081 | 0.196 | 14.08 |
| 32 | 1-0910-0925-12E | RTVSR | WFLNG | 9.117 | 0.081 | 2.798 | 0.110 | 3.258 | 39.01 |
| 33 | 1-0910-0925-1F | SIATH | MFLYG | 10.600 | 0.193 | 1.880 | 0.174 | 5.639 | 63.62 |
| 34 | 1-0910-0925-10G | SIHPF | SYLNG | 4.650 | 0.037 | 1.485 | 0.082 | 3.131 | 36.49 |
| 35 | 1-0910-4E | SIRPV | WFLDG | 0.800 | 0.004 | 0.888 | 0.030 | 0.900 | 7.94 |
| 36 | 1-0913-2B | SKHSP | WYLGK | 0.783 | 0.006 | 1.549 | 0.015 | 0.506 | 12.80 |
| 37 | 1-0910-1E | SMAPY | HFLQH | 2.300 | 0.030 | 1.369 | 0.019 | 1.680 | 22.44 |
| 38 | 1-0910-0925-10B | SMFTP | FFLRG | 14.233 | 0.170 | 1.052 | 0.039 | 13.536 | 42.30 |
| 39 | RV23H | SNAPQ | WYLSG | 10.750 | 0.093 | 1.562 | 0.065 | 6.881 | 13.46 |
| 40 | 1-0910-0925-11H | SRGNG | FFLRG | 5.783 | 0.034 | 0.893 | 0.072 | 6.478 | 44.21 |
| 41 | 1-0913-12F | SSPSH | AFLGH | 1.583 | 0.019 | 2.189 | 0.241 | 0.723 | 8.43 |
| 42 | 1-0910-0925-7F | SSRRP | FFANG | 5.283 | 0.034 | 1.532 | 0.097 | 3.448 | 43.89 |
| 43 | 1-0910-0925-8D | STIPS | FALRG | 6.867 | 0.044 | 0.914 | 0.040 | 7.511 | 38.80 |
| 44 | 1-0910-2F | TNQSA | FYLSS | 0.200 | 0.002 | 1.764 | 0.056 | 0.113 | 4.68 |
| 45 | RV24F | VPGIP | FALRG | 0.233 | 0.003 | 0.571 | 0.036 | 0.409 | *7.06* |
| 46 | 1-0910-5F | VTSTT | FFLNG | 3.850 | 0.027 | 1.236 | 0.066 | 3.115 | 20.76 |
| 47 | 1-0910-4F | YAPPH | WAIGQ | 1.964 | 0.116 | 1.648 | 0.214 | 1.192 | 5.34 |
| 48 | 1-0910-6C | YGRNM | FYAQG | 0.143 | 0.010 | 0.893 | 0.064 | 0.160 | 27.47 |
| 49 | 1-0910-3G | YRAKY | SFLNG | 3.864 | 0.135 | 1.890 | 0.095 | 2.045 | 47.90 |
| 50 | 1-0910-10E | YRMTP | FFLKS | 1.571 | 0.074 | 1.399 | 0.167 | 1.124 | 11.02 |
| 51 | RV25B | YRPSH | LFACC | 1.571 | 0.108 | 0.587 | 0.072 | 2.676 | 20.79 |
| 52 | 1-0910-9B | YRTVA | HYLQG | 5.071 | 0.331 | 0.897 | 0.121 | 5.655 | 34.77 |
|  | TAA |  |  | 0.000 | 0.005 | 0.000 | 0.001 |  |  |

Table S1 (B): data derived from H2H3 scFv phage display library.

| H2H3 | |  |  | VEGF  (ELISA ratio to AV1) | | Anti-E  (ELISA ratio to AV1) | | VEGF/anti-E ratio to AV1 (-TAA) | fXa/-fXa (-TAA)(%) |
| --- | --- | --- | --- | --- | --- | --- | --- | --- | --- |
|  | sequencing ID | H2 | H3 | average | stdev | average | stdev |  |  |
|  | AV1 | PAGGY | FFLPY | 1.000 | 0.172 | 1.000 | 0.067 | 1.000 | 15.86 |
| 1 | 13-1 | LTVPR | LFMFG | 2.367 | 0.157 | 8.533 | 0.435 | 0.277 | 15.98 |
| 2 | 16-1 | LSVPP | IWAGS | 1.896 | 0.286 | 9.589 | 0.252 | 0.198 | 22.56 |
| 3 | 17-1 | DRPSV | SPFYH | 0.830 | 0.122 | 7.289 | 0.108 | 0.114 | 18.10 |
| 4 | 20-1 | DMNFH | HFLFG | 2.893 | 0.184 | 5.506 | 0.072 | 0.525 | 27.46 |
| 5 | 2-1 | NPVPH | YYLAN | 0.581 | 0.027 | 1.961 | 0.089 | 0.297 | 16.91 |
| 6 | 24-1 | PPRLH | LWAYN | 4.019 | 0.169 | 5.372 | 0.296 | 0.748 | 32.56 |
| 7 | 26-1 | LTNPP | GAFAW | 1.193 | 0.099 | 8.783 | 0.366 | 0.136 | 3.18 |
| 8 | 30-1 | NPGCE | NSLWS | 1.119 | 0.055 | 7.350 | 0.366 | 0.152 | 9.69 |
| 9 | 40-1 | EPKIA | NFLQG | 0.252 | 0.017 | 7.894 | 0.303 | 0.032 | 6.76 |
| 10 | 4-1 | APPNL | MFLHN | 1.607 | 0.162 | 9.244 | 0.148 | 0.174 | 12.58 |
| 11 | 8-1 | VPFPQ | FFLMG | 0.100 | 0.006 | 3.583 | 0.122 | 0.028 | 8.34 |
| 12 | 01-1 | KPLPT | LFLNN | 1.585 | 0.113 | 3.922 | 0.102 | 0.404 | 17.05 |
| 13 | 03-1 | LTSPP | LWAQT | 4.544 | 0.214 | 6.022 | 0.271 | 0.755 | 34.65 |
| 14 | 04-1 | SNGIP | RSPSL | 1.393 | 0.008 | 6.517 | 0.186 | 0.214 | 11.22 |
| 15 | 05-1 | IPYPP | FFLNN | 2.078 | 0.128 | 1.889 | 0.054 | 1.100 | 20.99 |
| 16 | 07-1 | SGGIP | MPRNL | 3.859 | 0.047 | 6.083 | 0.423 | 0.634 | 9.51 |
| 17 | 10-1 | LPDPS | FWAFG | 3.093 | 0.425 | 5.622 | 0.202 | 0.550 | 21.04 |
| 18 | 12-1 | GPRLF | YFLPG | 1.289 | 0.113 | 3.094 | 0.067 | 0.417 | 10.10 |
| 19 | 15-1 | KPDPL | FFLGN | 3.630 | 0.130 | 5.511 | 0.153 | 0.659 | 29.97 |
| 20 | 16-1 | GPFPS | YFLNK | 5.574 | 0.101 | 9.289 | 0.091 | 0.600 | 48.05 |
| 21 | 18-1 | TPMPA | WAFGS | 5.522 | 0.192 | 8.283 | 0.016 | 0.667 | 48.50 |
| 22 | 19-1 | FTHPP | FFLQD | 0.541 | 0.027 | 0.967 | 0.020 | 0.559 | 9.91 |
| 23 | 21-1 | MHHPF | FFLDK | 0.448 | 0.045 | 1.650 | 0.017 | 0.272 | 10.96 |
| 24 | 53-1 | VPDPD | IYRQG | 2.607 | 0.076 | 3.889 | 0.132 | 0.670 | 32.66 |
| 25 | 54-1 | KPIPA | EFLQG | 2.181 | 0.200 | 5.328 | 0.172 | 0.409 | 25.01 |
| 26 | 56-1 | STGIP | RVPAS | 1.807 | 0.224 | 5.606 | 0.192 | 0.322 | 4.34 |
| 27 | 59-1 | NPNDS | YFLGG | 0.507 | 0.026 | 3.894 | 0.035 | 0.130 | 6.73 |
| 28 | 64-1 | PPPDL | FFLNL | 1.633 | 0.127 | 2.978 | 0.054 | 0.549 | 13.26 |
| 29 | 4-0910-10B | PEAPA | FFLMG | 2.637 | 0.133 | 4.606 | 0.143 | 0.573 | 23.82 |
| 30 | 4-0910-10B-4 | LTYPE | FAFGH | 1.541 | 0.127 | 4.411 | 0.158 | 0.349 | 7.93 |
| 31 | 4-0910-11C | ATGSP | RAKKF | 0.504 | 0.048 | 7.778 | 0.062 | 0.065 | 10.47 |
| 32 | 4-0910-12F | PAPDY | FFLGS | 3.093 | 0.113 | 4.683 | 0.064 | 0.660 | 38.53 |
| 33 | 4-0910-7A | ERLPF | FAFGH | 0.481 | 0.070 | 9.322 | 0.268 | 0.052 | 1.74 |
| 34 | 4-0910-8F | SAGVP | RLRWA | 1.807 | 0.173 | 7.933 | 0.455 | 0.228 | 12.33 |
| 35 | 4-0913-6D | TIFPP | YFLNH | 1.137 | 0.037 | 6.039 | 0.118 | 0.188 | 11.34 |
| 36 | 4-0913-6F | LTAPP | LFSSG | 1.311 | 0.077 | 3.978 | 0.158 | 0.330 | 14.69 |
| 37 | 4-0913-6F-3 | GPNVN | AFLQS | 0.444 | 0.040 | 6.578 | 0.164 | 0.068 | 3.57 |
| 38 | 4-0913-7A-2 | GRLPA | FYLTG | 0.300 | 0.023 | 5.806 | 0.291 | 0.052 | 0.61 |
| 39 | 4-0913-7A-4 | GLPPP | FYYQN | 0.956 | 0.078 | 4.667 | 0.326 | 0.205 | 15.93 |
| 40 | 4-0913-7B | LNAGA | WYAGP | 1.641 | 0.073 | 7.556 | 0.154 | 0.217 | 16.85 |
| 41 | 4-0913-7C | HPPHS | FFLRN | 3.615 | 0.259 | 6.950 | 0.339 | 0.520 | 34.91 |
| 42 | 4-0913-7E-4 | WYGVP | RLSTF | 1.281 | 0.088 | 8.767 | 0.121 | 0.146 | 25.70 |
| 43 | 4-0913-7F | QPPSM | FYLSG | 2.530 | 0.291 | 7.150 | 0.268 | 0.354 | 31.31 |
| 44 | 4-0913-7G | RPHPS | HWLSD | 0.341 | 0.020 | 8.472 | 0.318 | 0.040 | 3.31 |
| 45 | 4-0913-7G-2 | QPPST | FFLWA | 0.541 | 0.045 | 5.094 | 0.043 | 0.106 | 10.00 |
| 46 | 4-0913-8A | KPPVP | FFLDG | 2.396 | 0.044 | 6.300 | 0.231 | 0.380 | 30.33 |
| 47 | 4-0913-8B | PNSGL | FFLAG | 1.856 | 0.190 | 5.983 | 0.327 | 0.310 | 17.62 |
| 48 | 4-0913-8E | QPIPS | FFLRG | 5.152 | 0.087 | 6.161 | 0.108 | 0.836 | 65.20 |
| 49 | 4-0913-8G-2 | GPLPS | VFLFQ | 0.415 | 0.066 | 5.972 | 0.307 | 0.069 | 10.94 |
| 50 | 4-0913-9C | LSRDQ | LFRDG | 1.911 | 0.042 | 7.072 | 0.099 | 0.270 | 9.35 |
| 51 | 4-0919-11E | PNPRA | FFLFN | 4.926 | 0.219 | 5.283 | 0.016 | 0.932 | 54.45 |
| 52 | 4-0919-12E | LDKDP | FFGFG | 0.185 | 0.016 | 5.033 | 0.092 | 0.037 | 6.83 |
| 53 | 4-0919-12G | NPMSK | FFLSG | 2.122 | 0.121 | 5.900 | 0.153 | 0.360 | 16.16 |
| 54 | 4-0919-6E | GGDRA | LFLYG | 4.563 | 0.422 | 6.511 | 0.336 | 0.701 | 48.55 |
| 55 | 4-0919-6G | QMPDL | LFANG | 2.226 | 0.185 | 5.294 | 0.230 | 0.420 | 19.90 |
| 56 | 4-0919-6H | VPIHK | IFLFG | 0.467 | 0.026 | 2.894 | 0.153 | 0.161 | 23.15 |
| 57 | 4-0919-7E | SSGRP | RASEV | 1.833 | 0.279 | 8.517 | 0.198 | 0.215 | 50.39 |
| 58 | 4-0919-7H | PPPSP | QYLDS | 1.281 | 0.041 | 6.744 | 0.131 | 0.190 | 3.74 |
| 59 | 4-0919-8C | LSMPS | LYLNS | 1.430 | 0.117 | 4.511 | 0.094 | 0.317 | 23.36 |
| 60 | L4RV2C6G | LSPLP | FAYGT | 1.919 | 0.054 | 7.172 | 0.377 | 0.267 | 10.26 |
| 61 | L4RV2C6H | GYGQD | FFLND | 1.393 | 0.131 | 6.367 | 0.081 | 0.219 | 6.34 |
| 62 | L4RV2C7A | TPWPD | AFLSG | 3.674 | 0.353 | 6.089 | 0.102 | 0.603 | 52.11 |
| 63 | L4RV2C7D | SSGVP | RLRNC | 4.122 | 0.405 | 5.511 | 0.292 | 0.748 | 23.61 |
| 64 | L4RV2C7E | TPAPN | FFLNG | 3.056 | 0.096 | 3.194 | 0.036 | 0.957 | 39.65 |
| 65 | L4RV2C7E2 | TWNPS | LFLFG | 4.341 | 0.323 | 7.367 | 0.408 | 0.589 | 57.14 |
| 66 | L4RV2C9A | VPIPE | LYLRG | 4.678 | 0.331 | 4.189 | 0.088 | 1.117 | 44.20 |
| 67 | L4RV2C9C | KPDPI | FALRN | 0.322 | 0.022 | 5.389 | 0.012 | 0.060 | 8.57 |
| 68 | L4RV2C9E | LPQPS | LYLNS | 2.948 | 0.299 | 5.050 | 0.129 | 0.584 | 28.73 |
| 69 | L4RV2C9H | HTPMP | MFCIT | 0.174 | 0.018 | 2.822 | 0.183 | 0.062 | 8.53 |
|  | TAA |  |  | 0.000 | 0.001 | 0.000 | 0.003 |  |  |

Table S1(C): data derived from L2H3 scFv phage display library.

| L2H3 | |  |  | VEGF  (ELISA ratio to AV1) | | Anti-E  (ELISA ratio to AV1) | | VEGF/anti-E ratio to AV1 (-TAA) | fXa/-fXa (-TAA)(%) |
| --- | --- | --- | --- | --- | --- | --- | --- | --- | --- |
|  | Sequencing ID | L2 | H3 | average | stdev | average | stdev |  |  |
|  | AV1 | SASFL | FFLPY | 1.000 | 0.198 | 1.000 | 0.076 | 1.000 | 9.34 |
| 1 | L2H3-1 | SMRRH | FYLNG | 1.824 | 0.003 | 3.107 | 0.055 | 0.587 | 99.4 |
| 2 | L2H3-2 | PKNGR | MFLMG | 1.784 | 0.020 | 3.324 | 0.128 | 0.537 | 90.0 |
| 3 | L2H3-3 | LSIHR | FYISG | 1.807 | 0.017 | 3.560 | 0.039 | 0.508 | 99.3 |
| 4 | L2H3-4 | MASEA | FFLAG | 1.817 | 0.001 | 3.488 | 0.073 | 0.521 | 100.0 |
| 5 | L2H3-5 | GGSNI | FFLES | 1.805 | 0.014 | 3.592 | 0.006 | 0.503 | 97.2 |
| 6 | L2H3-6 | GAYYP | FFIGG | 1.814 | 0.008 | 3.673 | 0.005 | 0.494 | 99.9 |
| 7 | L2H3-7 | RTAIS | FFLSN | 1.808 | 0.003 | 3.660 | 0.007 | 0.494 | 98.8 |
| 8 | L2H3-8 | RTNAL | FWLRG | 1.802 | 0.002 | 3.634 | 0.040 | 0.496 | 95.2 |
| 9 | L2H3-9 | TAKHI | FFLSY | 2.335 | 0.053 | 3.236 | 0.196 | 0.721 | 75.6 |
| 10 | L2H3-10 | LRSWS | FYLGG | 2.363 | 0.017 | 2.985 | 0.235 | 0.792 | 96.9 |
| 11 | L2H3-11 | RRSFS | FYLYN | 2.364 | 0.021 | 3.553 | 0.063 | 0.665 | 95.5 |
| 12 | L2H3-12 | RSRFH | FYLDH | 2.242 | 0.119 | 3.091 | 0.435 | 0.725 | 89.3 |
| 13 | L2H3-13 | QLHYR | FFLQG | 2.363 | 0.042 | 3.556 | 0.163 | 0.665 | 94.1 |
| 14 | L2H3-14 | STHFP | FFLGA | 2.265 | 0.165 | 3.641 | 0.019 | 0.622 | 95.6 |
| 15 | L2H3-15 | QTLHL | FYLGH | 2.278 | 0.050 | 3.659 | 0.006 | 0.622 | 97.4 |
| 16 | L2H3-16 | KSGFP | FWLNN | 2.361 | 0.010 | 3.606 | 0.051 | 0.655 | 90.4 |
| 17 | L2H3-17 | LAKFS | FYLGK | 3.472 | 0.226 | 3.367 | 0.404 | 1.031 | 97.0 |
| 18 | L2H3-18 | TGATP | FWTFG | 2.637 | 0.358 | 3.632 | 0.020 | 0.726 | 40.4 |
| 19 | L2H3-19 | SAWRV | FFAGG | 3.499 | 0.218 | 3.692 | 0.054 | 0.948 | 98.5 |
| 20 | L2H3-20 | TSSSA | FFLSG | 3.508 | 0.166 | 3.687 | 0.010 | 0.952 | 83.0 |
| 21 | L2H3-21 | HSLML | FYLMG | 3.450 | 0.017 | 3.623 | 0.018 | 0.952 | 91.6 |
| 22 | L2H3-22 | RGSIS | FFLSG | 3.488 | 0.254 | 3.665 | 0.007 | 0.952 | 100.0 |
| 23 | L2H3-23 | GSLWL | FYLRS | 3.508 | 0.118 | 3.665 | 0.032 | 0.957 | 85.4 |
| 24 | L2H3-24 | GFQLL | FFLGG | 3.376 | 0.248 | 3.620 | 0.007 | 0.933 | 98.4 |
| 25 | L2H3-25 | QMRMR | FFLKN | 1.455 | 0.001 | 2.124 | 0.006 | 0.685 | 99.9 |
| 26 | L2H3-26 | RGVHL | FFLSS | 1.328 | 0.001 | 2.046 | 0.161 | 0.649 | 99.8 |
| 27 | L2H3-27 | SSRRN | FYLSR | 1.183 | 0.004 | 2.164 | 0.021 | 0.547 | 26.9 |
| 28 | L2H3-28 | SANMN | WFLSQ | 2.181 | 0.004 | 2.185 | 0.018 | 0.998 | 95.1 |
| 29 | L2H3-29 | GDWYN | FFLNY | 1.247 | 0.019 | 1.993 | 0.267 | 0.626 | 94.4 |
| 30 | L2H3-30 | FSLYS | FYLCG | 1.149 | 0.049 | 1.913 | 0.012 | 0.600 | 78.3 |
| 31 | L2H3-31 | ESLYA | FWLqG | 1.157 | 0.001 | 2.161 | 0.020 | 0.535 | 97.9 |
| 32 | L2H3-32 | LSTLQ | FYINN | 1.618 | 0.027 | 2.174 | 0.012 | 0.744 | 58.0 |
| 33 | L2H3-33 | qKELR | MFLYG | 2.316 | 0.028 | 1.993 | 0.141 | 1.162 | 88.8 |
| 34 | L2H3-34 | EHLSL | LAFSL | 3.344 | 0.094 | 2.031 | 0.087 | 1.647 | 82.5 |
| 35 | L2H3-35 | EAWHH | AAVVT | 1.583 | 0.079 | 2.106 | 0.041 | 0.751 | 76.9 |
| 36 | L2H3-36 | RASST | WFLSN | 1.525 | 0.096 | 2.165 | 0.003 | 0.704 | 19.1 |
| 37 | L2H3-37 | AALHH | LYLRG | 1.570 | 0.019 | 2.143 | 0.015 | 0.732 | 22.8 |
|  | TAA |  |  | 0.000 | 0.003 | 0.000 | 0.009 |  |  |

Table S1(D): data derived from L1H3 scFv phage display library.

| L1H3 | |  |  | VEGF  (ELISA ratio to AV1) | | Anti-E  (ELISA ratio to AV1) | | VEGF/anti-E ratio to AV1 (-TAA) | fXa/-fXa (-TAA)(%) |
| --- | --- | --- | --- | --- | --- | --- | --- | --- | --- |
|  | Sequencing ID | L1 | H3 | average | stdev | average | stdev |  |  |
|  | AV1 | DVSTA | FFLPY | 1.000 | 0.063 | 1.000 | 0.080 | 1.000 | 11.34 |
| 1 | L1H3-1 | ASGGG | MSFGN | 1.969 | 0.024 | 2.369 | 0.031 | 0.831 | 96.4 |
| 2 | L1H3-2 | SPETR | FFLNT | 1.950 | 0.019 | 2.351 | 0.007 | 0.830 | 87.6 |
| 3 | L1H3-3 | APGNR | LFLEG | 1.994 | 0.003 | 2.409 | 0.026 | 0.828 | 94.2 |
| 4 | L1H3-4 | TASSR | LFLSG | 1.981 | 0.002 | 2.275 | 0.021 | 0.871 | 96.1 |
| 5 | L1H3-5 | HLPGH | LYLYG | 1.990 | 0.003 | 2.344 | 0.079 | 0.849 | 99.5 |
| 6 | L1H3-6 | APSRR | LYLDG | 1.986 | 0.006 | 2.330 | 0.035 | 0.852 | 99.0 |
| 7 | L1H3-7 | VHFPY | FYLGG | 1.990 | 0.006 | 2.288 | 0.006 | 0.870 | 94.3 |
| 8 | L1H3-8 | MPSTA | HFLSG | 1.980 | 0.014 | 2.405 | 0.031 | 0.823 | 99.9 |
| 9 | L1H3-9 | MAGRR | WYLKG | 1.992 | 0.003 | 1.935 | 0.156 | 1.030 | 93.0 |
| 10 | L1H3-10 | SSAPA | AFLHY | 0.970 | 0.130 | 2.151 | 0.219 | 0.451 | 29.6 |
| 11 | L1H3-11 | PAGPA | FYLGS | 1.992 | 0.005 | 2.325 | 0.058 | 0.857 | 98.9 |
| 12 | L1H3-12 | PPARR | HYLSG | 1.993 | 0.004 | 2.408 | 0.061 | 0.827 | 99.1 |
| 13 | L1H3-13 | PIQGR | FALYD | 1.978 | 0.008 | 2.394 | 0.025 | 0.826 | 86.5 |
| 14 | L1H3-14 | SPLGR | WSTSG | 1.886 | 0.100 | 2.436 | 0.024 | 0.774 | 41.0 |
| 15 | L1H3-15 | PGPPR | HFRFN | 1.991 | 0.002 | 2.346 | 0.013 | 0.849 | 93.9 |
| 16 | L1H3-16 | EPAAR | WHLFG | 1.992 | 0.000 | 2.385 | 0.011 | 0.835 | 99.8 |
| 17 | L1H3-17 | STYGG | FWAGY | 1.843 | 0.001 | 2.184 | 0.104 | 0.844 | 68.8 |
| 18 | L1H3-18 | PLPPW | FSFGH | 1.879 | 0.001 | 2.204 | 0.010 | 0.853 | 99.8 |
| 19 | L1H3-19 | AGSPR | HFLLG | 0.723 | 0.188 | 0.898 | 0.004 | 0.805 | 32.5 |
| 20 | L1H3-20 | ELAGF | FFADR | 0.445 | 0.057 | 1.040 | 0.045 | 0.428 | 20.5 |
| 21 | L1H3-21 | SIRQR | WYVDG | 1.053 | 0.219 | 0.861 | 0.165 | 1.223 | 42.6 |
| 22 | L1H3-22 | SYGRN | EFLRG | 1.243 | 0.158 | 1.830 | 0.159 | 0.679 | 31.6 |
| 23 | L1H3-23 | PPFSA | FFLNY | 1.865 | 0.004 | 2.389 | 0.016 | 0.781 | 91.9 |
| 24 | L1H3-24 | RPHGR | HFLFY | 0.891 | 0.119 | 1.465 | 0.020 | 0.608 | 33.2 |
| 25 | L1H3-25 | IQQGA | AFLYG | 1.264 | 0.031 | 1.954 | 0.745 | 0.647 | 32.7 |
| 26 | L1H3-26 | TGGTG | FYLFG | 1.727 | 0.042 | 1.273 | 0.206 | 1.356 | 56.1 |
| 27 | L1H3-27 | GGRGG | FHLHG | 0.646 | 0.133 | 1.956 | 0.009 | 0.330 | 19.8 |
| 28 | L1H3-28 | PPGGP | FSHGL | 0.822 | 0.012 | 1.428 | 0.461 | 0.576 | 17.0 |
| 29 | L1H3-29 | AMSSR | HYLYD | 1.986 | 0.000 | 2.582 | 0.057 | 0.769 | 78.7 |
| 30 | L1H3-30 | TMLPR | FHLGD | 1.476 | 0.081 | 2.251 | 0.262 | 0.656 | 33.4 |
| 31 | L1H3-31 | GHVEQ | FFLRG | 1.869 | 0.047 | 1.844 | 0.202 | 1.013 | 66.7 |
| 32 | L1H3-32 | LTSAT | WYLGG | 1.833 | 0.116 | 2.192 | 0.058 | 0.836 | 75.2 |
| 33 | L1H3-33 | PGVPA | HFLYS | 1.708 | 0.087 | 1.257 | 0.337 | 1.359 | 38.4 |
| 34 | L1H3-34 | AGFGR | MFLYN | 2.346 | 0.086 | 2.468 | 0.360 | 0.951 | 72.8 |
| 35 | L1H3-35 | PTRGT | FAFGK | 2.087 | 0.113 | 2.423 | 0.345 | 0.861 | 29.3 |
| 36 | L1H3-36 | AGPNA | FYLLG | 2.276 | 0.022 | 1.204 | 0.029 | 1.891 | 62.2 |
| 37 | L1H3-37 | TPLGY | QYLFG | 2.350 | 0.072 | 2.406 | 0.161 | 0.977 | 86.8 |
| 38 | L1H3-38 | VPSGR | QFLYS | 0.340 | 0.183 | 1.884 | 0.280 | 0.180 | 35.6 |
| 39 | L1H3-39 | PTPPW | FFLGR | 1.657 | 0.671 | 2.085 | 0.165 | 0.795 | 93.2 |
| 40 | L1H3-40 | PLGPT | WFLGT | 2.233 | 0.021 | 2.692 | 0.153 | 0.829 | 71.5 |
| 41 | L1H3-41 | SVGLM | FHLFG | 0.869 | 0.068 | 1.557 | 0.023 | 0.558 | 50.0 |
| 42 | L1H3-42 | PPSQR | FFSHG | 1.912 | 0.020 | 2.728 | 0.020 | 0.701 | 67.7 |
| 43 | L1H3-43 | AMAHR | LYLQG | 1.841 | 0.004 | 2.577 | 0.159 | 0.714 | 92.4 |
| 44 | L1H3-44 | VLGPR | MFAGG | 1.993 | 0.003 | 2.777 | 0.035 | 0.718 | 99.4 |
|  | TAA |  |  | 0.000 | 0.004 | 0.000 | 0.008 |  |  |

Table S1(E): data derived from H1 scFv phage display library.

| H1, 5X | |  | VEGF  (ELISA ratio to AV1) | | Anti-E  (ELISA ratio to AV1) | | VEGF/anti-E ratio to AV1 (-TAA) |
| --- | --- | --- | --- | --- | --- | --- | --- |
|  | Sequencing ID | H1 | average | stdev | average | stdev |  |
|  | AV1 | ISDYW | 1.000 | 0.016 | 1.000 | 0.082 | 1.000 |
| 1 | 5x-1 | ISDYF | 0.965 | 0.091 | 1.030 | 0.061 | 0.938 |
| 2 | 5x-2 | LEDYW | 1.284 | 0.029 | 1.116 | 0.032 | 1.150 |
| 3 | 5x-3 | LENFW | 1.159 | 0.265 | 1.041 | 0.047 | 1.113 |
| 4 | 5x-4 | VRDYW | 1.237 | 0.008 | 1.037 | 0.087 | 1.193 |
| 5 | 5x-5 | IGNYW | 1.255 | 0.019 | 1.110 | 0.024 | 1.131 |
| 6 | 5x-6 | FDDFW | 1.246 | 0.017 | 1.237 | 0.026 | 1.007 |
| 7 | 5x-7 | LEDFW | 1.268 | 0.041 | 1.321 | 0.058 | 0.960 |
| 8 | 5x-8 | FKDFW | 1.185 | 0.036 | 1.216 | 0.038 | 0.975 |
| 9 | 5x-9 | VNEYW | 1.390 | 0.049 | 1.117 | 0.094 | 1.244 |
| 10 | 5x-10 | MSNYW | 1.351 | 0.028 | 1.044 | 0.112 | 1.294 |
| 11 | 5x-11 | IDDFW | 1.223 | 0.045 | 1.138 | 0.050 | 1.075 |
| 12 | 5x-12 | LSDYL | 0.967 | 0.040 | 1.472 | 0.120 | 0.657 |
| 13 | 5x-13 | LNNYW | 1.414 | 0.039 | 1.264 | 0.109 | 1.119 |
| 14 | 5x-14 | LGDFW | 1.252 | 0.029 | 1.218 | 0.075 | 1.029 |
| 15 | 5x-15 | FSHFW | 1.293 | 0.029 | 1.346 | 0.084 | 0.961 |
| 16 | 5x-16 | LDDYW | 1.452 | 0.071 | 1.258 | 0.083 | 1.155 |
| 17 | 5x-17 | LDEYW | 1.581 | 0.055 | 0.993 | 0.046 | 1.593 |
| 18 | 5x-18 | LEHYW | 1.572 | 0.172 | 1.028 | 0.021 | 1.529 |
| 19 | 5x-19 | LNSYW | 0.992 | 0.090 | 1.019 | 0.098 | 0.974 |
| 20 | 5x-20 | VDDYW | 1.647 | 0.089 | 0.947 | 0.044 | 1.738 |
| 21 | 5x-21 | VDNFW | 1.422 | 0.164 | 0.890 | 0.094 | 1.598 |
| 22 | 5x-22 | LNDYW | 1.352 | 0.035 | 1.152 | 0.058 | 1.174 |
| 23 | 5x-23 | VEDYF | 1.733 | 0.017 | 1.643 | 0.017 | 1.055 |
| 24 | 5x-24 | IREYW | 0.873 | 0.074 | 1.282 | 0.026 | 0.681 |
| 25 | 5x-25 | LSDYW | 1.217 | 0.025 | 0.650 | 0.152 | 1.871 |
| 26 | 5x-26 | LEDFF | 0.974 | 0.174 | 1.005 | 0.058 | 0.969 |
| 27 | 5x-27 | MEDFW | 1.542 | 0.059 | 0.873 | 0.041 | 1.765 |
| 28 | 5x-28 | MSDYF | 1.285 | 0.125 | 1.046 | 0.017 | 1.228 |
| 29 | 5x-29 | LSNYW | 1.357 | 0.152 | 0.799 | 0.155 | 1.698 |
| 30 | 5x-30 | LYEYW | 1.402 | 0.133 | 1.099 | 0.076 | 1.276 |
| 31 | 5x-31 | IRDYW | 1.326 | 0.144 | 0.992 | 0.206 | 1.338 |
| 32 | 5x-32 | VDDFW | 1.631 | 0.077 | 0.975 | 0.041 | 1.672 |
| 33 | 5x-33 | IDDYW | 1.066 | 0.141 | 0.972 | 0.030 | 1.097 |
| 34 | 5x-34 | VADFW | 0.857 | 0.073 | 0.967 | 0.179 | 0.887 |
| 35 | 5x-35 | MEDYW | 1.688 | 0.186 | 1.053 | 0.240 | 1.603 |
| 36 | 5x-36 | LDHFF | 0.823 | 0.088 | 1.217 | 0.161 | 0.676 |
| 37 | 5x-37 | LLDFW | 0.729 | 0.106 | 1.353 | 0.133 | 0.539 |
| 38 | 5x-38 | IRNYW | 0.905 | 0.033 | 1.496 | 0.229 | 0.605 |
| 39 | 5x-39 | IQDFF | 0.552 | 0.093 | 1.471 | 0.111 | 0.375 |
| 40 | 5x-40 | VTNYW | 1.195 | 0.078 | 1.397 | 0.151 | 0.855 |
| 41 | 5x-41 | LSNFW | 0.881 | 0.031 | 1.033 | 0.013 | 0.853 |
| 42 | 5x-42 | LYNYW | 0.958 | 0.074 | 1.150 | 0.063 | 0.834 |
|  | TAA |  | 0.000 | 0.032 | 0.000 | 0.032 |  |

1. Huang YJ, Chen IC, Yu CM, Lee YC, Hsu HJ, et al. (2010) Engineering anti-vascular endothelial growth factor single chain disulfide-stabilized antibody variable fragments (sc-dsFv) with phage-displayed sc-dsFv libraries. J Biol Chem 285: 7880-7891.

2. Chen IC, Yu CM, Lee YC, Huang YJ, Hsu HJ, et al. (2010) Signal sequence as a determinant in expressing disulfide-stabilized single chain antibody variable fragments (sc-dsFv) against human VEGF. Mol Biosyst 6: 1307-1315.
