# Supplementary material for: Rationalization and Design of the Complementarity Determining Region Sequences in an Antibody-Antigen Recognition Interface
Source: PLoS One. 2012 Mar 22;7(3):e33340. doi: 10.1371/journal.pone.0033340 (PMC3310866; doi:10.1371/journal.pone.0033340)
Supplement: Table S2 — Sequence information and VEGF-binding and expression data for the sc-dsFv variants shown in Figure 1 . (DOC) [file pone.0033340.s003.doc]

**Table S2**. Sequence information and VEGF-binding and expression data for the sc-dsFv variants shown in Figure 1. All the measurements shown in this Table were carried out in three repeats. The experimental details can be found in previous publications . These sc-dsFv phage display libraries contained signal sequence as MKKLLFAIPLVVPFYSqSMQPSSSLHHHGHGGSG, in which q as TAG amber codon could be translated as Q in *E. coli* ER2738 strain.

Table S2(A): data derived from L3H3-S5 sc-dsFv phage display library.

| L3H3-S5 | |  |  | VEGF  (ELISA ratio to AV1) | | Anti-E  (ELISA ratio to AV1) | | VEGF/anti-E ratio to AV1 (-TAA) | fXa/-fXa (-TAA)(%) |
| --- | --- | --- | --- | --- | --- | --- | --- | --- | --- |
|  | Sequencing ID | L3 | H3 | average | stdev | average | stdev |  |  |
|  | AV1 | YTTPP | FFLPY | 1.000 | 0.060 | 1.000 | 0.163 | 1.000 | 21.45 |
| 1 | 1 | TYMHR | FFANG | 1.412 | 0.143 | 0.188 | 0.033 | 7.529 | 85.39 |
| 2 | 2 | RHATK | EYLNH | 2.028 | 0.193 | 1.600 | 0.259 | 1.268 | 229.31 |
| 3 | 3 | RGPGA | FSHGQ | 2.359 | 0.268 | 1.481 | 0.240 | 1.592 | 99.41 |
| 4 | 4 | IMDPH | FYLRQ | 2.541 | 0.221 | 1.331 | 0.216 | 1.908 | 81.56 |
| 5 | 5 | MRGTY | FYLDN | 1.770 | 0.197 | 1.081 | 0.176 | 1.637 | 135.42 |
| 6 | 6 | TSKSS | FFAGM | 1.431 | 0.127 | 1.056 | 0.172 | 1.355 | 97.42 |
| 7 | 7 | SRSIY | MFAQG | 0.356 | 0.054 | 0.875 | 0.143 | 0.407 | 75.10 |
| 8 | 8 | LPSHP | WFANR | 1.031 | 0.037 | 2.125 | 0.343 | 0.485 | 57.07 |
| 9 | 9 | ELVPS | FWLQY | 2.162 | 0.141 | 0.500 | 0.083 | 4.325 | 63.14 |
| 10 | 10 | RRSSG | FFLHN | 1.070 | 0.094 | 0.413 | 0.069 | 2.594 | 108.87 |
| 11 | 11 | LRTFQ | FFLNG | 1.171 | 0.173 | 0.388 | 0.065 | 3.022 | 87.33 |
| 12 | 12 | LYNPQ | WFLRM | 1.294 | 0.099 | 0.944 | 0.154 | 1.371 | 110.85 |
| 13 | 13 | LFTMG | FSHRH | 1.084 | 0.079 | 1.388 | 0.225 | 0.781 | 103.77 |
| 14 | 14 | RGHSR | HYLFT | 0.179 | 0.012 | 0.913 | 0.149 | 0.196 | 45.95 |
| 15 | 15 | LVTPS | FFLYH | 0.501 | 0.055 | 0.606 | 0.100 | 0.827 | 72.07 |
| 16 | 16 | RRWPA | FAVGN | 0.361 | 0.053 | 0.956 | 0.156 | 0.378 | 115.13 |
| 17 | 6-0913-2B | NRSLY | FYAQK | 0.160 | 0.017 | 1.025 | 0.167 | 0.156 | 68.63 |
| 18 | 6-0913-2G | TGTKR | FWTQG | 0.639 | 0.063 | 0.600 | 0.099 | 1.064 | 70.14 |
| 19 | 6-0913-3C | RFDPA | HYLGS | 1.303 | 0.211 | 0.663 | 0.109 | 1.966 | 65.65 |
| 20 | 6-0913-5D | RLGNS | HYLMG | 0.776 | 0.084 | 1.044 | 0.170 | 0.743 | 48.32 |
| 21 | 6-0919-0925-11C | RLVPP | FFLRS | 1.401 | 0.129 | 0.188 | 0.033 | 7.470 | 66.92 |
| 22 | 6-0919-0925-2F | FVYPS | FSRKN | 0.106 | 0.013 | 0.838 | 0.137 | 0.127 | 53.15 |
| 23 | 6-0919-1A | RASGG | HYLFG | 1.417 | 0.197 | 0.856 | 0.140 | 1.655 | 77.15 |
| 24 | 6-0924-10A | YTGKY | FFISG | 0.126 | 0.017 | 0.381 | 0.064 | 0.331 | 65.03 |
| 25 | 6-0924-11B | VGAVA | HYLFG | 1.185 | 0.141 | 1.400 | 0.227 | 0.846 | 87.12 |
| 26 | 6-0924-11C | THSIN | FFRNG | 0.263 | 0.042 | 0.394 | 0.066 | 0.669 | 43.82 |
| 27 | 6-0924-11H | GAAPP | FFLNG | 1.521 | 0.265 | 0.325 | 0.055 | 4.680 | 78.11 |
| 28 | 6-0924-12D | SHSSK | AFLNN | 2.625 | 0.239 | 0.506 | 0.084 | 5.184 | 109.06 |
| 29 | 6-0924-1B | TYTRD | FFLNY | 0.216 | 0.021 | 0.225 | 0.039 | 0.959 | 111.01 |
| 30 | 6-0924-1C | WIDTY | HFAQG | 1.311 | 0.090 | 1.375 | 0.223 | 0.953 | 59.71 |
| 31 | 6-0924-2A | HNVPS | FALNK | 2.090 | 0.239 | 1.906 | 0.308 | 1.096 | 62.97 |
| 32 | 6-0924-2B | GHRTA | HHRAG | 0.431 | 0.057 | 2.138 | 0.345 | 0.202 | 63.08 |
| 33 | 6-0924-3B | VRSPG | HYLNN | 3.672 | 0.309 | 2.138 | 0.345 | 1.718 | 106.67 |
| 34 | 6-0924-3E | YSRVH | FFTPG | 0.218 | 0.028 | 0.813 | 0.133 | 0.269 | 72.71 |
| 35 | 6-0924-3F | TTVSP | FFLNN | 0.549 | 0.038 | 0.650 | 0.107 | 0.845 | 85.66 |
| 36 | 6-0924-4C | INGTF | WFTKG | 0.056 | 0.003 | 0.706 | 0.116 | 0.079 | 43.10 |
| 37 | 6-0924-4E | QLAPN | FYLAS | 0.300 | 0.038 | 0.444 | 0.074 | 0.675 | 92.71 |
| 38 | 6-0924-4H | RYTPS | WALDQ | 1.510 | 0.131 | 1.131 | 0.184 | 1.335 | 76.06 |
| 39 | 6-0924-5A | EHVPA | MFLDK | 0.655 | 0.067 | 1.250 | 0.203 | 0.524 | 61.88 |
| 40 | 6-0924-5C | IRARN | HYLFG | 0.224 | 0.026 | 1.500 | 0.243 | 0.149 | 53.62 |
| 41 | 6-0924-5D | NRDIT | FFLNK | 1.891 | 0.062 | 0.631 | 0.104 | 2.995 | 85.55 |
| 42 | 6-0924-5F | FLGLH | HFLHG | 2.347 | 0.237 | 0.600 | 0.099 | 3.912 | 78.01 |
| 43 | 6-0924-5G | IIGAH | HFRGG | 3.190 | 0.228 | 0.806 | 0.132 | 3.957 | 75.98 |
| 44 | 6-0924-6B | SPNYT | FFLER | 0.538 | 0.036 | 0.269 | 0.046 | 2.001 | 53.45 |
| 45 | 6-0924-6C | GTVPV | HYLQG | 2.050 | 0.267 | 0.481 | 0.080 | 4.261 | 72.33 |
| 46 | 6-0924-6E | LVHPT | FYTGG | 2.157 | 0.284 | 0.638 | 0.105 | 3.383 | 81.88 |
| 47 | 6-0924-6F | GLKKF | FYINN | 0.280 | 0.017 | 0.369 | 0.062 | 0.760 | 121.75 |
| 48 | 6-0924-8A | MPPTT | FFLGN | 0.692 | 0.073 | 0.469 | 0.078 | 1.476 | 86.09 |
| 49 | 6-0924-8E | NRPRY | LYADN | 0.244 | 0.018 | 0.769 | 0.126 | 0.317 | 80.06 |
| 50 | 6-0924-8F | WLIPA | HYLNN | 4.106 | 0.229 | 1.181 | 0.192 | 3.476 | 73.06 |
| 51 | 6-0924-9G | THHSQ | HYLSG | 3.882 | 0.158 | 1.156 | 0.188 | 3.358 | 60.23 |
| 52 | 6-0924-9H | RSVPS | FARRG | 2.297 | 0.329 | 1.038 | 0.169 | 2.214 | 33.91 |
|  | TAA |  |  | 0.000 | 0.001 | 0.000 | 0.003 |  |  |

Table S2(B): data derived from H2H3-S5 sc-dsFv phage display library.

| H2H3-S5 | |  |  | VEGF  (ELISA ratio to AV1) | | Anti-E  (ELISA ratio to AV1) | | VEGF/anti-E ratio to AV1 (-TAA) | fXa/-fXa (-TAA)(%) |
| --- | --- | --- | --- | --- | --- | --- | --- | --- | --- |
|  | sequencing ID | H2 | H3 | average | stdev | average | stdev |  |  |
|  | AV1 | PAGGY | FFLPY | 1.000 | 0.022 | 1.000 | 0.134 | 1.000 | 15.49 |
| 1 | I-Ren-2-1 | NFADY | NPYYQ | 6.888 | 0.171 | 4.550 | 0.424 | 1.514 | 93.28 |
| 2 | I-Ren-41-1 | IPPPN | LWARG | 1.766 | 0.062 | 1.801 | 0.124 | 0.981 | 93.26 |
| 3 | I-Ren-75-1 | LKQPP | LAYAG | 3.089 | 0.110 | 2.694 | 0.195 | 1.146 | 91.89 |
| 4 | 8-0810-1010-5G | GPWET | LFAKG | 4.117 | 0.075 | 3.010 | 0.241 | 1.368 | 71.94 |
| 5 | 8-0810-1010-6C | LTMPR | YAYDK | 2.645 | 0.136 | 2.625 | 0.108 | 1.007 | 80.86 |
| 6 | 8-0810-1013-1A | LHAPF | FYLQK | 1.785 | 0.044 | 1.667 | 0.106 | 1.071 | 103.78 |
| 7 | 8-0813-1010-12G | ALGGG | FFVMG | 1.056 | 0.014 | 0.852 | 0.009 | 1.239 | 113.02 |
| 8 | 8-0813-1010-1B | GPLPS | HFLSS | 1.304 | 0.045 | 2.931 | 0.178 | 0.445 | 67.54 |
| 9 | 8-0813-1010-2D | LSSPP | FFAQQ | 1.182 | 0.031 | 1.646 | 0.103 | 0.718 | 91.52 |
| 10 | 8-0813-1010-3C | HPIPS | FFLYG | 1.706 | 0.045 | 0.911 | 0.076 | 1.873 | 97.38 |
| 11 | 8-0813-1010-3F | LSVPP | LHSGS | 2.000 | 0.071 | 2.351 | 0.096 | 0.851 | 87.86 |
| 12 | 8-0813-1010-4F | DPPTD | FFLQQ | 0.631 | 0.033 | 3.234 | 0.131 | 0.195 | 60.46 |
| 13 | 8-0813-1010-6C | RDPNA | FFLQD | 0.364 | 0.015 | 3.271 | 0.151 | 0.111 | 260.92 |
| 14 | 8-0813-1010-7G | PLPRS | FYLAQ | 1.944 | 0.046 | 1.887 | 0.132 | 1.030 | 86.63 |
| 15 | 8-0813-1010-8B | SPWEW | LAYQT | 1.000 | 0.033 | 2.079 | 0.116 | 0.481 | 86.19 |
| 16 | 8-0813-1010-8G | GPHPS | FAYGQ | 1.266 | 0.038 | 2.337 | 0.200 | 0.542 | 102.92 |
| 17 | 8-0813-1010-9D | LGPRP | YSHGS | 2.014 | 0.069 | 3.430 | 0.275 | 0.587 | 67.96 |
| 18 | 8-0813-1010-9E | PQPPF | WHRGG | 2.738 | 0.084 | 3.375 | 0.073 | 0.811 | 82.81 |
| 19 | 8-0813-1010-9G | HYLRG | DPPSS | 4.907 | 0.246 | 3.983 | 0.257 | 1.232 | 74.37 |
| 20 | 8-0819-1010-12E | LNPTP | YAFNR | 4.528 | 0.122 | 4.172 | 0.224 | 1.085 | 98.33 |
| 21 | 8-0819-1010-6C | PMPNF | FFISG | 1.070 | 0.045 | 1.127 | 0.053 | 0.949 | 84.58 |
| 22 | 8-0820-1010-1A | NPLPT | SFLYG | 1.430 | 0.057 | 2.859 | 0.275 | 0.500 | 81.43 |
| 23 | 8-0820-1010-2A | GPIMD | LFLAS | 0.346 | 0.007 | 2.017 | 0.042 | 0.171 | 85.06 |
| 24 | 8-0820-1010-3C | VPDPL | FAYGN | 2.313 | 0.104 | 1.691 | 0.097 | 1.368 | 144.08 |
| 25 | 8-0820-1010-3F | NPMPN | LFLGR | 0.771 | 0.015 | 1.691 | 0.170 | 0.456 | 74.57 |
| 26 | 8-0820-1010-3H | FTMPP | VVSHQ | 1.598 | 0.076 | 2.656 | 0.255 | 0.602 | 52.36 |
| 27 | 8-0820-1010-4D | PMPSP | HFLSH | 1.407 | 0.036 | 2.832 | 0.069 | 0.497 | 84.05 |
| 28 | 8-0820-1010-4E | TGPVN | FAFGK | 1.107 | 0.057 | 3.134 | 0.306 | 0.353 | 104.68 |
| 29 | 8-0820-1010-4G | HPYPS | WAYFG | 0.336 | 0.020 | 2.024 | 0.136 | 0.166 | 98.77 |
| 30 | 8-0820-1010-6F | RNPHS | FYLFD | 0.874 | 0.040 | 1.124 | 0.024 | 0.778 | 107.76 |
| 31 | 8-0824-1010-3G | PMPIL | HFLAG | 2.636 | 0.058 | 3.134 | 0.251 | 0.841 | 91.38 |
| 32 | 8-0824-1010-7B | LPPVP | LFASQ | 1.238 | 0.034 | 1.979 | 0.183 | 0.626 | 53.24 |
| 33 | 8-0913-7H | PAPLL | FYAGS | 0.187 | 0.009 | 1.993 | 0.233 | 0.094 | 48.16 |
| 34 | 8-0913-8E | PLPYP | LFLQN | 1.706 | 0.084 | 2.302 | 0.344 | 0.741 | 72.88 |
| 35 | 8-0913-9D | LTIPY | SPYFQ | 2.771 | 0.097 | 2.780 | 0.124 | 0.997 | 75.86 |
| 36 | 8-0913-9H | PPPKH | LFLNG | 0.589 | 0.017 | 2.457 | 0.202 | 0.240 | 93.03 |
| 37 | 8-0919-0925-2F | LMDPL | FFLFG | 0.383 | 0.023 | 1.055 | 0.113 | 0.363 | 100.45 |
| 38 | 8-0919-0925-8B | PNPYA | FFTQN | 1.491 | 0.080 | 2.646 | 0.261 | 0.563 | 91.82 |
| 39 | 8-0919-6C | TPDPS | FSHFG | 3.864 | 0.126 | 3.643 | 0.080 | 1.061 | 93.88 |
| 40 | 8-0920-6F | QPMPA | HFLFG | 2.551 | 0.052 | 2.481 | 0.231 | 1.028 | 83.62 |
| 41 | 8-0920-6H | KPLPH | FFLKN | 1.738 | 0.114 | 1.608 | 0.221 | 1.081 | 82.48 |
| 42 | 8-0920-7B | LGVPP | LFRFG | 1.294 | 0.036 | 0.845 | 0.094 | 1.531 | 102.19 |
| 43 | 8-0920-7E | TPLPP | MSHGN | 2.234 | 0.015 | 3.368 | 0.197 | 0.663 | 98.15 |
| 44 | 8-0920-7F | PLPST | WSHFG | 2.883 | 0.057 | 4.100 | 0.214 | 0.703 | 75.19 |
| 45 | 8-0920-8C | PVPTP | FSHDH | 1.075 | 0.037 | 4.306 | 0.343 | 0.250 | 79.88 |
| 46 | 8-0920-8E | DSPAM | NPYYQ | 4.393 | 0.306 | 4.447 | 0.264 | 0.988 | 101.36 |
| 47 | 8-0924-10C | IPVPN | HYLQG | 3.995 | 0.152 | 3.601 | 0.320 | 1.109 | 99.29 |
| 48 | 8-0924-10G | IPLPS | SYLSG | 3.748 | 0.120 | 3.107 | 0.456 | 1.206 | 98.24 |
| 49 | 8-0924-1E | VNPND | WYLQR | 1.486 | 0.062 | 2.952 | 0.377 | 0.503 | 90.96 |
| 50 | 8-0924-3B | YPLDP | YFADK | 2.621 | 0.127 | 2.986 | 0.412 | 0.878 | 74.98 |
| 51 | 8-0924-4A | HPPSD | MYLSD | 1.841 | 0.003 | 1.887 | 0.317 | 0.976 | 87.39 |
| 52 | 8-0924-4E | LSMPP | LYMQD | 1.888 | 0.057 | 2.134 | 0.288 | 0.885 | 92.84 |
| 53 | 8-0924-6C | LYMPP | LSHNS | 3.481 | 0.075 | 2.729 | 0.366 | 1.276 | 97.76 |
| 54 | 8-0924-6D | EPVYS | FAFDK | 1.486 | 0.057 | 3.014 | 0.152 | 0.493 | 177.18 |
| 55 | 8-0924-7D | SPAPK | WSHGP | 0.084 | 0.003 | 2.976 | 0.341 | 0.028 | 102.62 |
| 56 | 8-0924-9H | LSPTP | FAFQT | 1.150 | 0.058 | 1.897 | 0.250 | 0.606 | 118.54 |
| 57 | L4RV2CS53A | LTLPP | AFLDN | 0.126 | 0.008 | 2.347 | 0.354 | 0.054 | 106.25 |
| 58 | L4RV2CS53C | SPRPE | FYLRK | 1.327 | 0.028 | 2.069 | 0.251 | 0.642 | 134.46 |
| 59 | L4RV2CS54A | SPFPE | FFARQ | 2.042 | 0.048 | 1.797 | 0.134 | 1.136 | 128.34 |
| 60 | L4RV2CS55H | LSRSS | MFAGG | 0.855 | 0.028 | 2.515 | 0.314 | 0.340 | 103.28 |
| 61 | L4RV2S5C5 | LHRPV | LFADQ | 1.037 | 0.007 | 1.918 | 0.199 | 0.541 | 130.97 |
|  | TAA |  |  | 0.000 | 0.001 | 0.000 | 0.002 |  |  |

Table S2(C): data derived from L2H3-S5 sc-dsFv phage display library.

| L2H3-S5 | |  |  | VEGF  (ELISA ratio to AV1) | | Anti-E  (ELISA ratio to AV1) | | VEGF/anti-E ratio to AV1 (-TAA) | fXa/-fXa (-TAA)(%) |
| --- | --- | --- | --- | --- | --- | --- | --- | --- | --- |
|  | Sequencing ID | L2 | H3 | average | stdev | average | stdev |  |  |
|  | AV1 | SASFL | FFLPY | 1.000 | 0.060 | 1.000 | 0.163 | 1.000 | 13.46 |
| 1 | 13-3-1117-3D | AATHS | FWLSN | 4.552 | 0.163 | 6.738 | 0.256 | 0.676 | 109.45 |
| 2 | 6G | DATHS | FWLSN | 1.644 | 0.197 | 2.019 | 0.094 | 0.814 | 98.22 |
| 3 | 13-1-1117-3C | DILYP | FFLNG | 2.683 | 0.305 | 1.475 | 0.078 | 1.819 | 98.62 |
| 4 | 3G | ESFYV | FYTKG | 0.779 | 0.102 | 1.988 | 0.107 | 0.392 | 69.90 |
| 5 | 3D | GANYR | FYLSF | 2.112 | 0.079 | 1.406 | 0.121 | 1.502 | 89.76 |
| 6 | 4A | GSTFQ | FAYKN | 0.922 | 0.092 | 2.319 | 0.088 | 0.397 | 70.75 |
| 7 | 3H | HAASA | FYLYT | 2.779 | 0.173 | 2.313 | 0.209 | 1.202 | 83.55 |
| 8 | 13m-2-1117-7C | HSSTT | FYLHN | 0.686 | 0.093 | 2.869 | 0.175 | 0.239 | 74.67 |
| 9 | 13-1108-11H | KRAYV | FYLNN | 1.902 | 0.136 | 8.600 | 0.477 | 0.221 | 86.02 |
| 10 | 13m-2-1117-9A | LASVP | FFTHN | 1.877 | 0.166 | 4.313 | 0.330 | 0.435 | 79.38 |
| 11 | 13-2-1117-7B | LSSIR | FFTKG | 0.106 | 0.013 | 5.425 | 0.494 | 0.020 | 48.39 |
| 12 | 6H | LTQRL | FWLSG | 0.345 | 0.047 | 2.294 | 0.237 | 0.150 | 103.34 |
| 13 | 7E | MANYV | EYLSH | 0.246 | 0.020 | 2.450 | 0.178 | 0.101 | 75.07 |
| 14 | 9F | MGSLV | FFLHH | 1.720 | 0.128 | 4.475 | 0.136 | 0.384 | 73.94 |
| 15 | 13-3-1117-7B | NHDTP | FFLNG | 0.910 | 0.090 | 2.156 | 0.146 | 0.422 | 84.43 |
| 16 | 7A | NQVFP | FAFSN | 1.196 | 0.137 | 1.581 | 0.096 | 0.756 | 115.28 |
| 17 | 5F | NVAYI | FFLFG | 1.703 | 0.152 | 7.113 | 0.384 | 0.239 | 110.81 |
| 18 | 13-2-1117-6B | QAHHL | FYLGN | 2.328 | 0.141 | 4.588 | 0.266 | 0.507 | 86.96 |
| 19 | 10D | QAHHS | FDLGN | 1.098 | 0.167 | 7.613 | 0.475 | 0.144 | 103.17 |
| 20 | 13-2-1117-7H | QKVYR | FSRGG | 0.328 | 0.055 | 6.775 | 0.618 | 0.048 | 59.98 |
| 21 | 1A | QTSTT | FFTRS | 2.193 | 0.160 | 4.363 | 0.253 | 0.503 | 75.49 |
| 22 | 7C | RAYRL | WYTSG | 1.098 | 0.106 | 1.444 | 0.049 | 0.761 | 88.48 |
| 23 | 4H | RISFR | FWAGS | 3.269 | 0.187 | 5.738 | 0.382 | 0.570 | 101.18 |
| 24 | 1C | RTGKE | FFLWG | 4.308 | 0.212 | 7.756 | 0.294 | 0.555 | 107.65 |
| 25 | 13-2-1117-8A | RTGTA | FFLWG | 2.784 | 0.099 | 5.038 | 0.308 | 0.553 | 73.38 |
| 26 | 1E | RTGTK | FFLRG | 3.182 | 0.042 | 3.844 | 0.248 | 0.828 | 90.57 |
| 27 | 8B | SAHLN | FYTRG | 1.689 | 0.210 | 6.419 | 0.348 | 0.263 | 85.24 |
| 28 | 5D | SALYR | FYVSG | 1.415 | 0.197 | 3.769 | 0.365 | 0.375 | 100.44 |
| 29 | 13-2-1117-9C | SLRNR | FFLRG | 1.510 | 0.075 | 5.263 | 0.348 | 0.287 | 74.74 |
| 30 | 10C | STRHL | WHLGK | 0.874 | 0.073 | 1.831 | 0.083 | 0.477 | 78.03 |
| 31 | 13-2-1117-11A | SVTRK | FFLNG | 3.129 | 0.122 | 3.050 | 0.164 | 1.026 | 78.29 |
| 32 | 1G | SVTRK | FFLKG | 1.345 | 0.140 | 6.238 | 0.483 | 0.216 | 94.58 |
| 33 | 7D | SVTRK | FFVNG | 3.090 | 0.180 | 7.963 | 0.253 | 0.388 | 99.44 |
| 34 | 11A | YAHLN | FYTRG | 1.266 | 0.099 | 6.744 | 0.389 | 0.188 | 82.91 |
| 35 | 8D | YALYR | FYVSG | 0.513 | 0.054 | 5.406 | 0.356 | 0.095 | 62.21 |
|  | TAA |  |  | 0.000 | 0.001 | 0.000 | 0.003 |  |  |

Table S2(D): data derived from L1H3-S5 sc-dsFv phage display library.

| L1H3-S5 | |  |  | VEGF  (ELISA ratio to AV1) | | Anti-E  (ELISA ratio to AV1) | | VEGF/anti-E ratio to AV1 (-TAA) | fXa/-fXa (-TAA)(%) |
| --- | --- | --- | --- | --- | --- | --- | --- | --- | --- |
|  | Sequencing ID | L1 | H3 | average | stdev | average | stdev |  |  |
|  | AV1 | DVSTA | FFLPY | 1.000 | 0.172 | 1.000 | 0.067 | 1.000 | 15.78 |
| 1 | 15-1-1105-1H | PPAGQ | WYLSG | 2.715 | 0.198 | 2.706 | 0.102 | 1.003 | 76.62 |
| 2 | 15-1-1105-2A | SPRTD | HWLSG | 0.822 | 0.109 | 2.411 | 0.062 | 0.341 | 90.39 |
| 3 | 15-1-1105-3C | PVGRS | FYLKQ | 2.548 | 0.100 | 3.228 | 0.102 | 0.789 | 73.48 |
| 4 | 15-1121-4C | PVPPR | FHLQH | 2.519 | 0.122 | 2.011 | 0.041 | 1.252 | 75.58 |
| 5 | 15-1123-1A | GVGAQ | FYADH | 1.474 | 0.133 | 2.483 | 0.102 | 0.594 | 83.21 |
| 6 | 15-1123-1C | AVNSR | SYLNG | 2.167 | 0.224 | 3.994 | 0.329 | 0.542 | 63.82 |
| 7 | 15-1123-1D | AVRGQ | FYLMN | 2.167 | 0.230 | 2.322 | 0.169 | 0.933 | 86.28 |
| 8 | 15-1123-1E | AVLRR | FYTQG | 1.252 | 0.091 | 1.017 | 0.018 | 1.231 | 92.17 |
| 9 | 15-1123-1G | QPYRS | FSHDG | 1.570 | 0.260 | 3.789 | 0.335 | 0.414 | 82.12 |
| 10 | 15-1123-1H | PAASR | WYLRQ | 1.011 | 0.131 | 1.961 | 0.208 | 0.516 | 110.73 |
| 11 | 15-1123-2A | PAMDL | WYTLG | 0.422 | 0.015 | 1.033 | 0.038 | 0.409 | 68.22 |
| 12 | 15-1123-5B | SLASS | FAFDR | 1.226 | 0.085 | 2.244 | 0.157 | 0.546 | 130.55 |
| 13 | 15-1123-5C | PIRHS | FAFGG | 3.007 | 0.282 | 1.672 | 0.094 | 1.798 | 90.08 |
| 14 | 15-1123-5D | AVPSR | WYTGG | 1.470 | 0.199 | 2.167 | 0.076 | 0.679 | 94.02 |
| 15 | 15-1123-6A | SPRRG | LYLAG | 0.389 | 0.040 | 1.150 | 0.100 | 0.338 | 104.43 |
| 16 | 15-1123-6C | LPLGR | HFIRG | 2.167 | 0.129 | 2.150 | 0.044 | 1.008 | 87.03 |
| 17 | 15-1123-6D | SVAGR | FAFQS | 1.170 | 0.118 | 1.561 | 0.128 | 0.750 | 87.20 |
| 18 | 15-1123-7G | PTYDR | FALNQ | 2.226 | 0.231 | 3.272 | 0.356 | 0.680 | 108.71 |
| 19 | 15-1123-7H | PPRGA | FYRGN | 1.541 | 0.107 | 2.578 | 0.133 | 0.598 | 85.33 |
| 20 | 15-1123-8B | RVLPR | HYLQQ | 0.448 | 0.030 | 2.528 | 0.101 | 0.177 | 105.27 |
| 21 | 15-2-1105-3E | PPSQR | HSHYG | 1.622 | 0.068 | 4.456 | 0.171 | 0.364 | 84.77 |
| 22 | 15-3-1105-12A | DVGQS | WFLQT | 2.944 | 0.324 | 3.494 | 0.206 | 0.843 | 87.66 |
| 23 | 15-3-1105-3D | GVSSR | WSRDQ | 1.167 | 0.022 | 2.794 | 0.247 | 0.417 | 79.27 |
| 24 | 15-3-1105-4A | DTQRR | LYREG | 0.578 | 0.030 | 2.006 | 0.135 | 0.288 | 80.00 |
| 25 | 15-3-1105-5D | RPLGR | FALQG | 7.916 | 0.215 | 3.828 | 0.035 | 2.068 | 96.53 |
| 26 | 15-3-1105-5H | PVGTR | FHLYH | 4.827 | 0.435 | 3.615 | 0.043 | 1.335 | 67.72 |
| 27 | 15-3-1105-8E | PPASA | FWTSG | 4.173 | 0.336 | 2.093 | 0.146 | 1.994 | 84.01 |
| 28 | 15-4-1105-11D | QTNQQ | HFLNG | 6.879 | 0.384 | 3.811 | 0.100 | 1.805 | 90.11 |
| 29 | 15-4-1105-3A | SPLAR | MYRMG | 1.150 | 0.078 | 1.474 | 0.026 | 0.780 | 57.67 |
| 30 | 15-4-1109-3H | TLTQD | FYLRG | 3.491 | 0.365 | 0.880 | 0.042 | 3.968 | 83.13 |
| 31 | 15B-1C | YVPNL | FSHGS | 2.411 | 0.330 | 2.093 | 0.114 | 1.152 | 76.22 |
| 32 | 15m-1-1105-10F | IVGNN | WSYGQ | 1.729 | 0.183 | 2.326 | 0.237 | 0.743 | 77.57 |
| 33 | 15m-1-1105-6C | DMLSR | FWLDN | 1.505 | 0.170 | 1.612 | 0.152 | 0.934 | 155.87 |
| 34 | 15m-1121-10G | QAGVR | FWLLG | 0.907 | 0.099 | 1.014 | 0.035 | 0.894 | 85.62 |
| 35 | 15m-1121-12G | PLTAA | FYLSK | 1.565 | 0.171 | 1.158 | 0.025 | 1.352 | 92.78 |
| 36 | 15m-1121-5A | HLPPR | WHRGQ | 2.117 | 0.197 | 1.825 | 0.074 | 1.160 | 87.56 |
| 37 | 15m-1121-9G | PRLSQ | FAFNG | 2.640 | 0.270 | 1.186 | 0.059 | 2.227 | 71.63 |
| 38 | 15m-2-1105-8A | PISRR | FALSE | 0.701 | 0.043 | 1.515 | 0.074 | 0.463 | 149.35 |
| 39 | 15m-3-1105-11F | PPSQR | FSLQQ | 1.981 | 0.160 | 2.340 | 0.104 | 0.847 | 176.88 |
| 40 | 15m-3-1105-11G | PPLRM | HYTFG | 0.799 | 0.071 | 1.361 | 0.102 | 0.587 | 66.10 |
| 41 | 15m-3-1105-9B | GIGAR | FYAYA | 1.023 | 0.048 | 1.237 | 0.052 | 0.827 | 40.93 |
| 42 | 15m-4-1105-10A | GIKNE | FYLRS | 0.650 | 0.068 | 1.491 | 0.053 | 0.436 | 112.05 |
| 43 | 15m-4-1105-3G | FTGAS | FYLDS | 1.280 | 0.095 | 1.148 | 0.029 | 1.116 | 137.13 |
| 44 | 15m-4-1109-07B | VLAGA | HYLFG | 1.364 | 0.103 | 1.124 | 0.006 | 1.214 | 87.62 |
| 45 | 15m-4-1109-08A | WAANR | FAREH | 0.650 | 0.031 | 2.440 | 0.152 | 0.266 | 27.41 |
| 46 | 15m-4-1109-10E | ALPPR | FYSDH | 1.411 | 0.103 | 1.261 | 0.071 | 1.119 | 106.70 |
| 47 | 15mB-8F | SVSSR | WVTYG | 0.668 | 0.016 | 0.904 | 0.046 | 0.739 | 48.35 |
| 48 | 15mS-8H | ENTQA | FYLHG | 1.028 | 0.086 | 0.780 | 0.043 | 1.318 | 166.63 |
| 49 | 15S-12C | PVANR | HAFYN | 1.963 | 0.071 | 5.010 | 0.192 | 0.392 | 70.12 |
| 50 | Ala-F05 | PIPAR | FWLEL | 1.921 | 0.094 | 1.498 | 0.216 | 1.282 | 92.95 |
| 51 | Ala-F07 | PPASR | FAFNH | 2.925 | 0.280 | 1.660 | 0.054 | 1.762 | 108.44 |
| 52 | Ala-F15 | SPRAR | WAFqG | 2.103 | 0.194 | 1.405 | 0.380 | 1.496 | 74.52 |
| 53 | Ala-m-F22 | SPSGR | FSHYH | 1.000 | 0.037 | 2.113 | 0.107 | 0.473 | 31.01 |
|  | TAA |  |  | 0.000 | 0.001 | 0.000 | 0.003 |  |  |

1. Huang YJ, Chen IC, Yu CM, Lee YC, Hsu HJ, et al. (2010) Engineering anti-vascular endothelial growth factor single chain disulfide-stabilized antibody variable fragments (sc-dsFv) with phage-displayed sc-dsFv libraries. J Biol Chem 285: 7880-7891.

2. Chen IC, Yu CM, Lee YC, Huang YJ, Hsu HJ, et al. (2010) Signal sequence as a determinant in expressing disulfide-stabilized single chain antibody variable fragments (sc-dsFv) against human VEGF. Mol Biosyst 6: 1307-1315.
