# Supplementary material for: Rationalization and Design of the Complementarity Determining Region Sequences in an Antibody-Antigen Recognition Interface
Source: PLoS One. 2012 Mar 22;7(3):e33340. doi: 10.1371/journal.pone.0033340 (PMC3310866; doi:10.1371/journal.pone.0033340)
Supplement: Table S3 — Data collection and refinement statistics for the sc-dsFv x-ray crystallography. (DOC) [file pone.0033340.s004.doc]

**Table S3.** Data collection and refinement statistics for the sc-dsFv x-ray crystallography. Values in parentheses in data collection correspond to the highest-resolution shell.

| ***Data collection*** | |
| --- | --- |
| Unit cell parameters (Å) | *a* = *b* =136.063, *c* =169.004 |
| Space group | *P*3(1)21 |
| Resolution range (Å) | 30.0 - 2.4 (2.49 - 2.40) |
| No. of molecules in an ASU | 6 |
| Total no. of reflections | 574,450 (50,805) |
| Unique reflections | 70,426 (6,598) |
| Redundancy | 8.2 (7.7) |
| Completeness (%) | 99.1 (93.9) |
| Average *I*/σ(*I*) | 13.8 (2.45) |
| *R*_merge_ (%) | 12.2 (45.1) |
| ***Refinement*** | |
| No. of reflections [> 0 σ(*F*)], working/test | 63877/3233 |
| *R*_working_ /*R*_free_ (5.1% data) | 0.2158/0.2514 |
| r.m.s.d. bond distance (Å)/bond angle (°) | 0.020/1.792 |
| Average *B* value (Å^2^)/no. of atoms | |
| All nonhydrogen atoms | 31.50/10863 |
| Protein | 31.29/10322 |
| Water | 35.60/541 |
| Ramachandran plot (excluding prolines and glycines) | |
| Residues in most favored regions | 997 (87.8%) |
| Residues in additional allowed regions | 117 (10.3%) |
| Residues in generously allowed regions | 15 (1.3%) |
| Residues in disallowed regions | 6 (0.5%) |
