# Supplementary material for: Rationalization and Design of the Complementarity Determining Region Sequences in an Antibody-Antigen Recognition Interface
Source: PLoS One. 2012 Mar 22;7(3):e33340. doi: 10.1371/journal.pone.0033340 (PMC3310866; doi:10.1371/journal.pone.0033340)
Supplement: Table S4 — Leave-one-out cross validation predictions for the amino acid preferences at each of the 30 CDR interface residues. (DOC) [file pone.0033340.s005.doc]

**Table S4.** Leave-one-out cross validation predictions for the amino acid preferences at each of the 30 CDR interface residues. Experimental amino acid preferences *W_ji_* and δ*W_ji_* are compared with the predicted amino acid preferences *pW_ji_* and δ*pW_ji_*. The *X_ji_*, *Y_ji_*, and *Z_ji_* as shown in Equation (4) are described in Equations (1)~(3). The corresponding optimized parameters (*x_ji_*, *y_ji_*, *z_ji_*, *a_ji_*) and threshold *t_i_* for each of the amino acid type *i* at each position *j* of the 30 CDR interface positions are also shown. The Pearson correlation coefficients shown at the bottom of each Table were calculated for the *W_ji_-X_ji_*, *W_ji_-Y_ji_*, *W_ji_-Z_ji_* , and *W_ji_-pW_ji_* correlation, respectively.

| Position | 28CDR1L | | | | | | | | | | | |
| --- | --- | --- | --- | --- | --- | --- | --- | --- | --- | --- | --- | --- |
| Amino Acid | W_ji_ | δW_ji_ | X_ji_ | Y_ji_ | Z_ji_ | x | y | z | a | t | pW_ji_-t | δpW_ji_ |
| P | 3.92 | 1 | 40.72 | 2.16 | -2.40 | 0.06 | 0.15 | 0.15 | -1.48 | 0.25 | 0.47 | 1 |
| A | 2.80 | 1 | 27.09 | 2.08 | -1.57 | -0.04 | 0.27 | -0.43 | -1.22 | 0.06 | 0.19 | 1 |
| S | 1.36 | 1 | 34.25 | 1.50 | 1.79 | -0.08 | 0.18 | 3.08 | -0.59 | 0.32 | 0.61 | 1 |
| E | 0.98 | 1 | 47.35 | 0.39 | -0.99 | 0.05 | 0.04 | -0.49 | -3.69 | 0.23 | 0.07 | 1 |
| M | 0.98 | 1 | 49.40 | 0.24 | -2.40 | 0.07 | 0.09 | 0.49 | -2.21 | 0.50 | 0.00 | 1 |
| T | 0.94 | 1 | 34.71 | -0.94 | -1.40 | -0.03 | -0.04 | -1.08 | -0.18 | 0.77 | -0.22 | 0 |
| V | 0.20 | 1 | 34.85 | 1.59 | -2.40 | 0.04 | -0.18 | 0.16 | -4.73 | 0.70 | -0.69 | 0 |
| H | -0.76 | 0 | 64.26 | -0.99 | -2.40 | 0.00 | 0.04 | 0.22 | 0.25 | 0.18 | 0.17 | 1 |
| I | -0.76 | 0 | 42.18 | 1.60 | -2.40 | -0.01 | -0.02 | 0.65 | 0.17 | 0.60 | -0.46 | 0 |
| G | -0.80 | 0 | 20.07 | 1.67 | -0.77 | -0.10 | -0.30 | 0.33 | 0.68 | 0.27 | -0.15 | 0 |
| L | -3.10 | 0 | 42.21 | 1.38 | -2.40 | 0.00 | -0.04 | 0.78 | -0.45 | 0.21 | -0.14 | 0 |
| R | -3.10 | 0 | 71.14 | -0.42 | -0.77 | -0.08 | 0.39 | 0.53 | 3.90 | 0.10 | -0.01 | 0 |
| C | -5.86 | 0 | 34.45 | 0.58 | -2.40 | 0.00 | 0.00 | 0.00 | -102.6 | 0.50 | -0.50 | 0 |
| D | -5.86 | 0 | 40.83 | 0.26 | 1.63 | 0.05 | -0.03 | -0.80 | -2.64 | 0.25 | -0.12 | 0 |
| F | -5.86 | 0 | 73.20 | 1.56 | -2.40 | 0.14 | 0.34 | 1.32 | -5.53 | 0.47 | 0.40 | 1 |
| K | -5.86 | 0 | 48.74 | 0.51 | -2.40 | 0.06 | -0.05 | -1.17 | -6.39 | 0.70 | -0.35 | 0 |
| N | -5.86 | 0 | 48.65 | -0.01 | 1.29 | 0.02 | 0.11 | 0.49 | -0.77 | 0.15 | 0.56 | 1 |
| Q | -5.86 | 0 | 56.20 | 0.44 | -1.12 | -0.07 | 0.51 | 2.79 | 2.34 | 0.70 | -0.69 | 0 |
| W | -5.86 | 0 | 97.36 | -3.13 | -2.40 | 0.03 | 0.13 | 0.33 | -3.13 | 0.70 | -0.44 | 0 |
| Y | -5.86 | 0 | 80.48 | -0.07 | -2.40 | 0.02 | 0.05 | 0.77 | -2.24 | 0.19 | -0.10 | 0 |
| Pearson correlation coefficient to W_ji_ | | | -0.52 | 0.40 | -0.06 |  | | | | | 0.31 |  |

| Position | 29CDR1L | | | | | | | | | | | |
| --- | --- | --- | --- | --- | --- | --- | --- | --- | --- | --- | --- | --- |
| Amino Acid | W_ji_ | δW_ji_ | X_ji_ | Y_ji_ | Z_ji_ | x | y | z | a | t | pW_ji_-t | δpW_ji_ |
| P | 4.15 | 1 | 16.79 | 0.75 | -2.40 | 0.07 | 0.18 | 0.22 | -1.73 | 0.25 | 0.04 | 1 |
| G | 2.44 | 1 | 11.21 | 0.74 | -2.40 | -0.13 | -0.35 | 0.86 | 0.80 | 0.27 | -0.22 | 0 |
| M | 2.06 | 1 | 35.17 | -0.47 | -2.40 | 0.09 | 0.12 | 0.61 | -2.75 | 0.50 | -0.26 | 0 |
| H | 0.98 | 1 | 20.77 | 0.09 | -2.40 | 0.00 | 0.01 | 0.45 | 0.05 | 0.18 | 0.07 | 1 |
| I | 0.98 | 1 | 16.99 | 0.40 | 2.21 | -0.01 | -0.03 | 0.44 | -0.12 | 0.60 | 0.05 | 1 |
| T | 0.94 | 1 | 11.03 | 0.46 | -2.40 | -0.03 | -0.02 | -1.13 | -0.27 | 0.77 | 0.13 | 1 |
| L | 0.48 | 1 | 15.54 | 0.43 | 0.12 | 0.00 | -0.06 | 0.61 | -0.97 | 0.21 | 0.08 | 1 |
| A | 0.20 | 1 | 10.81 | 0.92 | -2.40 | -0.02 | 0.36 | -0.18 | -1.23 | 0.06 | 0.28 | 1 |
| Y | -0.68 | 0 | 17.79 | 0.74 | -2.40 | 0.02 | 0.05 | 0.78 | -2.15 | 0.19 | -0.16 | 0 |
| S | -1.72 | 0 | 14.63 | 0.58 | -2.40 | -0.08 | 0.20 | 3.22 | -0.64 | 0.32 | -0.32 | 0 |
| Q | -2.34 | 0 | 16.24 | 0.42 | -2.40 | -0.08 | 0.50 | 2.80 | 2.47 | 0.70 | -0.69 | 0 |
| V | -2.34 | 0 | 10.69 | 0.45 | 1.50 | 0.08 | 0.03 | -0.90 | -6.44 | 0.70 | -0.70 | 0 |
| C | -5.86 | 0 | 14.63 | 0.27 | -2.40 | 0.00 | 0.00 | 0.00 | -102.6 | 0.50 | -0.50 | 0 |
| D | -5.86 | 0 | 10.61 | 0.60 | -2.40 | 0.05 | -0.06 | -1.16 | -2.52 | 0.25 | 0.42 | 1 |
| E | -5.86 | 0 | 14.64 | 0.44 | -2.40 | 0.07 | 0.10 | -0.84 | -4.29 | 0.23 | -0.01 | 0 |
| F | -5.86 | 0 | 38.51 | 0.55 | -1.14 | 0.09 | 0.19 | 1.24 | -3.46 | 0.47 | -0.26 | 0 |
| K | -5.86 | 0 | 43.29 | 0.23 | -2.40 | 0.05 | -0.07 | -1.09 | -5.82 | 0.70 | -0.46 | 0 |
| N | -5.86 | 0 | 14.62 | 0.69 | -2.40 | 0.00 | 0.07 | 0.20 | -0.35 | 0.15 | 0.18 | 1 |
| R | -5.86 | 0 | 63.01 | 0.45 | -1.29 | -0.08 | 0.42 | 0.43 | 3.73 | 0.10 | 0.10 | 1 |
| W | -5.86 | 0 | 24.52 | 0.36 | -2.40 | 0.03 | 0.11 | 0.36 | -2.68 | 0.70 | -0.65 | 0 |
| Pearson correlation coefficient to W_ji_ | | | -0.34 | -0.02 | 0.12 |  | | | | | 0.19 |  |

| Position | 30CDR1L | | | | | | | | | | | |
| --- | --- | --- | --- | --- | --- | --- | --- | --- | --- | --- | --- | --- |
| Amino Acid | W_ji_ | δW_ji_ | X_ji_ | Y_ji_ | Z_ji_ | x | y | z | a | t | pW_ji_-t | δpW_ji_ |
| G | 3.41 | 1 | 4.96 | 2.25 | 0.53 | -0.11 | -0.44 | 0.18 | 0.53 | 0.27 | 0.02 | 1 |
| F | 2.06 | 1 | 56.19 | -1.46 | -2.40 | 0.12 | 0.22 | 1.78 | -4.78 | 0.47 | -0.41 | 0 |
| S | 1.71 | 1 | 18.18 | 4.77 | 1.42 | -0.07 | 0.19 | 3.15 | -0.63 | 0.32 | 0.65 | 1 |
| A | 1.53 | 1 | 11.13 | 4.33 | -2.40 | -0.02 | 0.26 | -0.35 | -1.27 | 0.06 | 0.57 | 1 |
| P | 1.53 | 1 | 25.11 | 2.13 | -2.40 | 0.06 | 0.14 | 0.18 | -1.54 | 0.25 | 0.23 | 1 |
| Y | -0.68 | 0 | 63.18 | -7.65 | 0.08 | 0.02 | 0.03 | 0.77 | -2.11 | 0.19 | 0.07 | 1 |
| E | -0.76 | 0 | 30.80 | 1.21 | -0.99 | 0.07 | 0.11 | -0.63 | -4.02 | 0.23 | 0.00 | 0 |
| H | -0.76 | 0 | 49.11 | -4.43 | -0.09 | -0.01 | 0.00 | 0.34 | 0.64 | 0.18 | 0.31 | 1 |
| L | -0.79 | 0 | 32.99 | -0.61 | -0.20 | 0.00 | -0.02 | 0.95 | -0.40 | 0.21 | 0.18 | 1 |
| R | -0.79 | 0 | 57.39 | -4.68 | 0.33 | -0.09 | 0.33 | 0.60 | 4.16 | 0.10 | 0.01 | 1 |
| Q | -0.80 | 0 | 40.17 | -1.01 | -1.12 | -0.07 | 0.49 | 2.77 | 2.39 | 0.70 | -0.68 | 0 |
| V | -0.80 | 0 | 26.03 | 0.43 | 0.00 | 0.08 | 0.04 | -0.86 | -6.31 | 0.70 | -0.68 | 0 |
| C | -5.86 | 0 | 18.34 | 1.68 | -2.40 | 0.00 | 0.00 | 0.00 | -102.6 | 0.50 | -0.50 | 0 |
| D | -5.86 | 0 | 18.52 | 2.94 | 0.20 | 0.05 | -0.04 | -0.83 | -2.60 | 0.25 | -0.13 | 0 |
| I | -5.86 | 0 | 33.26 | 0.09 | -1.36 | -0.01 | -0.03 | 0.68 | 0.25 | 0.60 | -0.33 | 0 |
| K | -5.86 | 0 | 41.36 | -5.38 | -0.63 | 0.04 | -0.10 | -0.92 | -5.34 | 0.70 | -0.63 | 0 |
| M | -5.86 | 0 | 33.53 | -2.83 | -2.40 | 0.09 | 0.16 | 0.27 | -3.00 | 0.50 | -0.26 | 0 |
| N | -5.86 | 0 | 33.11 | -3.17 | 1.03 | 0.01 | 0.05 | 0.44 | -0.46 | 0.15 | 0.36 | 1 |
| T | -5.86 | 0 | 25.24 | 3.39 | -0.34 | -0.02 | 0.03 | -1.16 | -0.26 | 0.77 | -0.33 | 0 |
| W | -5.86 | 0 | 81.06 | -7.75 | -2.40 | 0.03 | 0.11 | 0.35 | -2.83 | 0.70 | -0.61 | 0 |
| Pearson correlation coefficient to W_ji_ | | | -0.17 | 0.27 | 0.12 |  | | | | | 0.45 |  |

| Position | 31CDR1L | | | | | | | | | | | |
| --- | --- | --- | --- | --- | --- | --- | --- | --- | --- | --- | --- | --- |
| Amino Acid | W_ji_ | δW_ji_ | X_ji_ | Y_ji_ | Z_ji_ | x | y | z | a | t | pW_ji_-t | δpW_ji_ |
| G | 4.35 | 1 | 0.00 | 0.28 | -0.49 | -0.09 | -0.30 | 0.35 | 0.53 | 0.27 | 0.30 | 1 |
| P | 3.68 | 1 | 5.73 | 1.22 | -2.38 | 0.09 | 0.21 | 0.24 | -2.04 | 0.25 | -0.12 | 0 |
| N | 0.98 | 1 | 0.00 | 0.20 | 1.72 | 0.02 | 0.08 | 0.17 | -0.94 | 0.15 | 0.20 | 1 |
| T | 0.20 | 1 | 4.40 | 0.35 | 1.47 | -0.02 | -0.09 | -2.16 | -1.00 | 0.77 | -0.76 | 0 |
| R | -0.08 | 0 | 33.77 | 0.31 | -0.73 | -0.17 | 0.66 | 0.35 | 8.86 | 0.10 | 0.85 | 1 |
| E | -0.76 | 0 | 6.21 | 0.21 | -2.38 | 0.06 | 0.09 | -0.73 | -4.13 | 0.23 | -0.11 | 0 |
| H | -0.76 | 0 | 6.70 | 0.22 | -2.38 | -0.03 | -0.02 | 0.18 | 1.36 | 0.18 | 0.49 | 1 |
| S | -0.79 | 0 | 0.00 | 0.30 | 1.01 | -0.29 | 0.81 | 14.08 | -2.34 | 0.32 | 0.68 | 1 |
| A | -0.80 | 0 | 0.00 | 0.22 | -2.38 | -0.03 | 0.29 | -0.82 | -1.51 | 0.06 | 0.57 | 1 |
| Q | -0.80 | 0 | 20.32 | 0.17 | -2.38 | -0.08 | 0.50 | 2.81 | 2.46 | 0.70 | -0.70 | 0 |
| L | -3.10 | 0 | 4.40 | 0.43 | -2.38 | -0.01 | -0.07 | 0.71 | -0.24 | 0.21 | -0.09 | 0 |
| C | -5.86 | 0 | 0.00 | 0.19 | -2.38 | 0.00 | 0.00 | 0.00 | -102.6 | 0.50 | -0.50 | 0 |
| D | -5.86 | 0 | 0.00 | 0.18 | -0.51 | 0.04 | -0.05 | -0.83 | -2.53 | 0.25 | -0.14 | 0 |
| F | -5.86 | 0 | 32.20 | 0.35 | -2.38 | 0.09 | 0.19 | 1.27 | -3.69 | 0.47 | -0.44 | 0 |
| I | -5.86 | 0 | 6.20 | 0.65 | -0.81 | -0.03 | -0.07 | 0.73 | 0.73 | 0.60 | -0.11 | 0 |
| K | -5.86 | 0 | 17.67 | 0.18 | 0.40 | 0.04 | -0.10 | -0.95 | -5.47 | 0.70 | -0.69 | 0 |
| M | -5.86 | 0 | 5.40 | 0.19 | -2.38 | 0.09 | 0.16 | 0.32 | -2.95 | 0.50 | -0.46 | 0 |
| V | -5.86 | 0 | 6.02 | 0.79 | -1.47 | 0.08 | 0.03 | -0.90 | -6.35 | 0.70 | -0.69 | 0 |
| W | -5.86 | 0 | 35.36 | 0.31 | -2.38 | 0.03 | 0.11 | 0.35 | -2.67 | 0.70 | -0.63 | 0 |
| Y | -5.86 | 0 | 30.77 | 0.38 | 0.33 | 0.02 | 0.05 | 0.75 | -1.78 | 0.19 | 0.07 | 1 |
| Pearson correlation coefficient to W_ji_ | | | -0.31 | 0.17 | 0.16 |  | | | | | 0.48 |  |

| Position | 32CDR1L | | | | | | | | | | | |
| --- | --- | --- | --- | --- | --- | --- | --- | --- | --- | --- | --- | --- |
| Amino Acid | W_ji_ | δW_ji_ | X_ji_ | Y_ji_ | Z_ji_ | x | y | z | a | t | pW_ji_-t | δpW_ji_ |
| R | 4.23 | 1 | 44.36 | -2.66 | -0.39 | -0.07 | 0.64 | 0.66 | 3.56 | 0.10 | 0.06 | 1 |
| A | 2.44 | 1 | 0.00 | 0.58 | 0.07 | 0.01 | 0.35 | -1.17 | -2.83 | 0.06 | 0.00 | 1 |
| Y | 1.07 | 1 | 43.79 | -0.16 | 2.51 | 0.02 | 0.03 | 0.72 | -2.29 | 0.19 | 0.42 | 1 |
| W | 0.98 | 1 | 57.14 | -2.63 | 1.00 | 0.04 | 0.11 | 0.28 | -4.13 | 0.70 | -0.56 | 0 |
| G | 0.94 | 1 | 0.00 | 0.04 | -1.04 | -0.09 | -0.29 | 0.41 | 0.53 | 0.27 | 0.25 | 1 |
| T | 0.20 | 1 | 5.55 | 0.79 | -2.38 | -0.03 | -0.02 | -1.14 | -0.28 | 0.77 | 0.14 | 1 |
| F | -0.76 | 0 | 35.52 | 1.28 | 0.82 | 0.11 | 0.26 | 1.82 | -3.37 | 0.47 | 0.43 | 1 |
| H | -0.76 | 0 | 35.41 | -1.46 | -0.05 | -0.02 | 0.01 | 0.35 | 0.84 | 0.18 | 0.37 | 1 |
| M | -0.76 | 0 | 20.72 | -0.86 | -2.38 | 0.09 | 0.16 | 0.29 | -2.92 | 0.50 | -0.38 | 0 |
| N | -0.76 | 0 | 11.94 | 1.36 | 0.61 | 0.00 | 0.06 | 0.40 | -0.30 | 0.15 | 0.36 | 1 |
| P | -2.34 | 0 | 0.00 | 1.27 | -2.38 | 0.07 | 0.18 | 0.09 | -1.57 | 0.25 | -0.07 | 0 |
| Q | -2.34 | 0 | 20.42 | 0.15 | -2.38 | -0.08 | 0.50 | 2.81 | 2.46 | 0.70 | -0.70 | 0 |
| C | -5.86 | 0 | 6.53 | 0.18 | -2.38 | 0.00 | 0.00 | 0.00 | -102.6 | 0.50 | -0.50 | 0 |
| D | -5.86 | 0 | 0.00 | 1.28 | 0.11 | 0.05 | -0.05 | -0.83 | -2.59 | 0.25 | -0.19 | 0 |
| E | -5.86 | 0 | 10.94 | 0.08 | -2.38 | 0.07 | 0.09 | -0.78 | -4.19 | 0.23 | -0.07 | 0 |
| I | -5.86 | 0 | 15.93 | 0.21 | -2.38 | -0.02 | -0.04 | 0.63 | 0.33 | 0.60 | -0.41 | 0 |
| K | -5.86 | 0 | 28.95 | -0.89 | -0.58 | 0.04 | -0.10 | -0.93 | -5.37 | 0.70 | -0.67 | 0 |
| L | -5.86 | 0 | 19.12 | 1.69 | -1.60 | -0.01 | -0.05 | 0.76 | -0.33 | 0.21 | -0.07 | 0 |
| S | -5.86 | 0 | 0.00 | 0.25 | -0.95 | -0.07 | 0.19 | 3.15 | -0.60 | 0.32 | -0.29 | 0 |
| V | -5.86 | 0 | 5.18 | 1.29 | -2.38 | 0.08 | 0.03 | -0.91 | -6.30 | 0.70 | -0.68 | 0 |
| Pearson correlation coefficient to W_ji_ | | | 0.42 | -0.45 | 0.47 |  | | | | | 0.52 |  |

| Position | 50CDR2L | | | | | | | | | | | |
| --- | --- | --- | --- | --- | --- | --- | --- | --- | --- | --- | --- | --- |
| Amino Acid | W_ji_ | δW_ji_ | X_ji_ | Y_ji_ | Z_ji_ | x | y | z | a | t | pW_ji_-t | δpW_ji_ |
| E | 2.51 | 1 | 34.39 | -0.51 | -0.67 | 0.09 | 0.08 | -1.39 | -6.29 | 0.23 | -0.13 | 0 |
| G | 1.98 | 1 | 0.00 | 0.05 | 0.19 | -0.09 | -0.30 | 0.31 | 0.54 | 0.27 | 0.37 | 1 |
| R | 1.81 | 1 | 48.95 | -0.09 | 0.45 | -0.09 | 0.31 | 0.28 | 4.03 | 0.10 | 0.33 | 1 |
| S | 0.92 | 1 | 10.43 | 1.67 | 0.65 | -0.07 | 0.18 | 3.07 | -0.72 | 0.32 | 0.37 | 1 |
| Q | 0.64 | 1 | 33.48 | 0.41 | -0.58 | -0.07 | 0.53 | 2.87 | 2.23 | 0.70 | -0.51 | 0 |
| T | 0.64 | 1 | 16.69 | 1.51 | -0.91 | -0.03 | -0.04 | -1.13 | -0.34 | 0.77 | -0.23 | 0 |
| L | 0.35 | 1 | 25.59 | 0.01 | -0.49 | -0.01 | -0.10 | 0.70 | -0.61 | 0.21 | 0.01 | 1 |
| F | -0.34 | 0 | 44.96 | 0.44 | -1.14 | 0.09 | 0.20 | 1.25 | -3.49 | 0.47 | -0.15 | 0 |
| H | -0.34 | 0 | 43.97 | 0.29 | -0.69 | -0.01 | 0.03 | 0.30 | 0.54 | 0.18 | 0.30 | 1 |
| K | -0.34 | 0 | 26.30 | -0.65 | 0.45 | 0.04 | -0.10 | -0.94 | -5.46 | 0.70 | -0.69 | 0 |
| M | -0.34 | 0 | 26.77 | -0.40 | -0.65 | 0.09 | 0.16 | 0.72 | -2.00 | 0.50 | -0.05 | 0 |
| A | -1.95 | 0 | 5.11 | 0.99 | 0.23 | -0.02 | 0.35 | -0.34 | -1.16 | 0.06 | 0.21 | 1 |
| P | -1.95 | 0 | 0.00 | 0.04 | -2.40 | 0.07 | 0.18 | 0.09 | -1.57 | 0.25 | -0.11 | 0 |
| C | -5.65 | 0 | 13.15 | 0.44 | -2.40 | 0.00 | 0.00 | 0.00 | -102.6 | 0.50 | -0.50 | 0 |
| D | -5.65 | 0 | 18.58 | 1.97 | 0.33 | 0.05 | -0.04 | -0.82 | -2.59 | 0.25 | -0.14 | 0 |
| I | -5.65 | 0 | 26.51 | -0.30 | -2.40 | -0.01 | -0.04 | 0.64 | 0.27 | 0.60 | -0.43 | 0 |
| N | -5.65 | 0 | 25.42 | -0.05 | -0.33 | 0.01 | 0.07 | 0.31 | -0.42 | 0.15 | 0.26 | 1 |
| V | -5.65 | 0 | 19.45 | 0.17 | -2.40 | 0.08 | 0.03 | -0.93 | -6.25 | 0.70 | -0.63 | 0 |
| W | -5.65 | 0 | 73.94 | -2.56 | 1.50 | 0.03 | 0.13 | 0.47 | -2.95 | 0.70 | -0.24 | 0 |
| Y | -5.65 | 0 | 57.68 | 0.24 | 1.46 | 0.03 | 0.08 | 0.90 | -2.48 | 0.19 | 0.43 | 1 |
| Pearson correlation coefficient to W_ji_ | | | -0.15 | 0.12 | 0.14 |  | | | | | 0.25 |  |

| Position | 51CDR2L | | | | | | | | | | | |
| --- | --- | --- | --- | --- | --- | --- | --- | --- | --- | --- | --- | --- |
| Amino Acid | W_ji_ | δW_ji_ | X_ji_ | Y_ji_ | Z_ji_ | x | y | z | a | t | pW_ji_-t | δpW_ji_ |
| A | 3.58 | 1 | 0 | 0 | 1.85 | N/A | N/A | N/A | N/A | N/A | N/A | N/A |
| S | 2.77 | 1 | 0 | 0 | -2.40 | N/A | N/A | N/A | N/A | N/A | N/A | N/A |
| K | 1.43 | 1 | 0 | 0 | -2.40 | N/A | N/A | N/A | N/A | N/A | N/A | N/A |
| M | 1.43 | 1 | 0 | 0 | 0.81 | N/A | N/A | N/A | N/A | N/A | N/A | N/A |
| G | 1.38 | 1 | 0 | 0 | -0.18 | N/A | N/A | N/A | N/A | N/A | N/A | N/A |
| T | 1.38 | 1 | 0 | 0 | 1.34 | N/A | N/A | N/A | N/A | N/A | N/A | N/A |
| D | -0.34 | 0 | 0 | 0 | -1.38 | N/A | N/A | N/A | N/A | N/A | N/A | N/A |
| F | -0.34 | 0 | 0 | 0 | -2.40 | N/A | N/A | N/A | N/A | N/A | N/A | N/A |
| H | -0.34 | 0 | 0 | 0 | -2.40 | N/A | N/A | N/A | N/A | N/A | N/A | N/A |
| R | -1.31 | 0 | 0 | 0 | -2.40 | N/A | N/A | N/A | N/A | N/A | N/A | N/A |
| L | -2.73 | 0 | 0 | 0 | -2.40 | N/A | N/A | N/A | N/A | N/A | N/A | N/A |
| C | -5.65 | 0 | 0 | 0 | -2.40 | N/A | N/A | N/A | N/A | N/A | N/A | N/A |
| E | -5.65 | 0 | 0 | 0 | -2.40 | N/A | N/A | N/A | N/A | N/A | N/A | N/A |
| I | -5.65 | 0 | 0 | 0 | -1.36 | N/A | N/A | N/A | N/A | N/A | N/A | N/A |
| N | -5.65 | 0 | 0 | 0 | -1.21 | N/A | N/A | N/A | N/A | N/A | N/A | N/A |
| P | -5.65 | 0 | 0 | 0 | -2.40 | N/A | N/A | N/A | N/A | N/A | N/A | N/A |
| Q | -5.65 | 0 | 0 | 0 | -2.40 | N/A | N/A | N/A | N/A | N/A | N/A | N/A |
| V | -5.65 | 0 | 0 | 0 | 0.12 | N/A | N/A | N/A | N/A | N/A | N/A | N/A |
| W | -5.65 | 0 | 0 | 0 | -2.40 | N/A | N/A | N/A | N/A | N/A | N/A | N/A |
| Y | -5.65 | 0 | 0 | 0 | -2.40 | N/A | N/A | N/A | N/A | N/A | N/A | N/A |
| Pearson correlation coefficient to W_ji_ | | | N/A | N/A | 0.46 |  | | | | | N/A |  |

| Position | 52CDR2L | | | | | | | | | | | |
| --- | --- | --- | --- | --- | --- | --- | --- | --- | --- | --- | --- | --- |
| Amino Acid | W_ji_ | δW_ji_ | X_ji_ | Y_ji_ | Z_ji_ | x | y | z | a | t | pW_ji_-t | δpW_ji_ |
| N | 2.51 | 1 | 0 | 0 | 0.13 | N/A | N/A | N/A | N/A | N/A | N/A | N/A |
| W | 2.51 | 1 | 0 | 0 | -2.40 | N/A | N/A | N/A | N/A | N/A | N/A | N/A |
| L | 1.81 | 1 | 0 | 0 | -2.40 | N/A | N/A | N/A | N/A | N/A | N/A | N/A |
| S | 1.81 | 1 | 0 | 0 | 2.44 | N/A | N/A | N/A | N/A | N/A | N/A | N/A |
| H | 1.43 | 1 | 0 | 0 | -0.69 | N/A | N/A | N/A | N/A | N/A | N/A | N/A |
| K | 1.43 | 1 | 0 | 0 | -1.43 | N/A | N/A | N/A | N/A | N/A | N/A | N/A |
| R | 0.35 | 1 | 0 | 0 | -2.40 | N/A | N/A | N/A | N/A | N/A | N/A | N/A |
| Y | -0.26 | 0 | 0 | 0 | -1.09 | N/A | N/A | N/A | N/A | N/A | N/A | N/A |
| E | -0.34 | 0 | 0 | 0 | -2.40 | N/A | N/A | N/A | N/A | N/A | N/A | N/A |
| I | -0.34 | 0 | 0 | 0 | -2.40 | N/A | N/A | N/A | N/A | N/A | N/A | N/A |
| A | -0.38 | 0 | 0 | 0 | -2.40 | N/A | N/A | N/A | N/A | N/A | N/A | N/A |
| G | -1.95 | 0 | 0 | 0 | -2.40 | N/A | N/A | N/A | N/A | N/A | N/A | N/A |
| Q | -1.95 | 0 | 0 | 0 | -2.40 | N/A | N/A | N/A | N/A | N/A | N/A | N/A |
| T | -1.95 | 0 | 0 | 0 | 0.29 | N/A | N/A | N/A | N/A | N/A | N/A | N/A |
| V | -1.95 | 0 | 0 | 0 | -2.40 | N/A | N/A | N/A | N/A | N/A | N/A | N/A |
| C | -5.65 | 0 | 0 | 0 | -2.40 | N/A | N/A | N/A | N/A | N/A | N/A | N/A |
| D | -5.65 | 0 | 0 | 0 | -0.88 | N/A | N/A | N/A | N/A | N/A | N/A | N/A |
| F | -5.65 | 0 | 0 | 0 | -1.14 | N/A | N/A | N/A | N/A | N/A | N/A | N/A |
| M | -5.65 | 0 | 0 | 0 | -2.40 | N/A | N/A | N/A | N/A | N/A | N/A | N/A |
| P | -5.65 | 0 | 0 | 0 | -2.40 | N/A | N/A | N/A | N/A | N/A | N/A | N/A |
| Pearson correlation coefficient to W_ji_ | | | N/A | N/A | 0.26 |  | | | | | N/A |  |

| Position | 53CDR2L | | | | | | | | | | | |
| --- | --- | --- | --- | --- | --- | --- | --- | --- | --- | --- | --- | --- |
| Amino Acid | W_ji_ | δW_ji_ | X_ji_ | Y_ji_ | Z_ji_ | x | y | z | a | t | pW_ji_-t | δpW_ji_ |
| H | 4.42 | 1 | 45.16 | -0.37 | -0.69 | -0.02 | -0.02 | 0.30 | 0.59 | 0.18 | 0.21 | 1 |
| Y | 4.01 | 1 | 55.22 | 2.22 | 0.28 | 0.02 | 0.00 | 0.89 | -2.52 | 0.19 | 0.03 | 1 |
| F | 3.92 | 1 | 50.33 | 0.63 | -0.59 | 0.09 | 0.17 | 1.22 | -3.79 | 0.47 | 0.06 | 1 |
| M | 2.51 | 1 | 29.24 | -1.73 | -2.40 | 0.11 | 0.17 | 0.68 | -3.44 | 0.50 | -0.38 | 0 |
| I | 1.43 | 1 | 28.24 | -0.28 | -0.52 | -0.02 | -0.07 | 0.61 | 0.13 | 0.60 | -0.27 | 0 |
| W | 1.43 | 1 | 74.29 | -0.24 | -2.40 | 0.03 | 0.04 | 0.71 | -3.56 | 0.70 | -0.66 | 0 |
| E | -0.34 | 0 | 34.48 | -1.97 | -1.46 | 0.07 | 0.10 | -0.76 | -4.27 | 0.23 | 0.04 | 1 |
| N | -0.34 | 0 | 21.23 | -0.16 | 2.00 | 0.00 | 0.06 | 0.62 | -0.37 | 0.15 | 0.56 | 1 |
| L | -0.36 | 0 | 28.07 | -0.04 | -2.40 | -0.01 | -0.06 | 0.76 | -0.37 | 0.21 | -0.13 | 0 |
| R | -0.36 | 0 | 51.01 | 0.10 | 0.45 | -0.08 | 0.49 | 0.97 | 4.41 | 0.10 | 0.59 | 1 |
| S | -0.36 | 0 | 6.17 | 0.90 | 0.40 | -0.08 | 0.21 | 3.33 | -0.32 | 0.32 | 0.34 | 1 |
| A | -1.95 | 0 | 6.28 | 0.51 | -1.57 | -0.02 | 0.32 | -0.56 | -1.37 | 0.06 | 0.33 | 1 |
| G | -1.95 | 0 | 0.00 | 0.09 | -2.40 | -0.12 | -0.39 | 0.09 | 0.89 | 0.27 | 0.38 | 1 |
| T | -1.95 | 0 | 13.05 | 0.67 | 1.34 | -0.03 | -0.03 | -1.15 | -0.17 | 0.77 | -0.66 | 0 |
| C | -5.65 | 0 | 13.49 | 0.32 | -2.40 | 0.00 | 0.00 | 0.00 | -102.6 | 0.50 | -0.50 | 0 |
| D | -5.65 | 0 | 19.87 | 0.93 | -1.38 | 0.05 | -0.04 | -0.94 | -2.60 | 0.25 | 0.17 | 1 |
| K | -5.65 | 0 | 28.62 | -0.35 | 0.12 | 0.04 | -0.10 | -0.93 | -5.44 | 0.70 | -0.69 | 0 |
| P | -5.65 | 0 | 13.86 | 0.69 | -2.40 | 0.07 | 0.19 | 0.06 | -1.60 | 0.25 | 0.08 | 1 |
| Q | -5.65 | 0 | 36.09 | -0.41 | -1.12 | -0.07 | 0.49 | 2.73 | 2.36 | 0.70 | -0.67 | 0 |
| V | -5.65 | 0 | 20.53 | 0.31 | -1.51 | 0.08 | 0.03 | -0.87 | -6.25 | 0.70 | -0.66 | 0 |
| Pearson correlation coefficient to W_ji_ | | | 0.54 | -0.06 | 0.20 |  | | | | | 0.31 |  |

| Position | 54CDR2L | | | | | | | | | | | |
| --- | --- | --- | --- | --- | --- | --- | --- | --- | --- | --- | --- | --- |
| Amino Acid | W_ji_ | δW_ji_ | X_ji_ | Y_ji_ | Z_ji_ | x | y | z | a | t | pW_ji_-t | δpW_ji_ |
| H | 3.30 | 1 | 0 | 0 | -2.40 | N/A | N/A | N/A | N/A | N/A | N/A | N/A |
| N | 2.51 | 1 | 0 | 0 | -2.40 | N/A | N/A | N/A | N/A | N/A | N/A | N/A |
| L | 1.81 | 1 | 0 | 0 | 1.89 | N/A | N/A | N/A | N/A | N/A | N/A | N/A |
| I | 1.43 | 1 | 0 | 0 | -2.40 | N/A | N/A | N/A | N/A | N/A | N/A | N/A |
| S | 1.40 | 1 | 0 | 0 | -0.67 | N/A | N/A | N/A | N/A | N/A | N/A | N/A |
| P | 1.38 | 1 | 0 | 0 | -2.40 | N/A | N/A | N/A | N/A | N/A | N/A | N/A |
| R | 0.92 | 1 | 0 | 0 | 1.77 | N/A | N/A | N/A | N/A | N/A | N/A | N/A |
| A | 0.64 | 1 | 0 | 0 | -1.57 | N/A | N/A | N/A | N/A | N/A | N/A | N/A |
| Q | -1.95 | 0 | 0 | 0 | -1.12 | N/A | N/A | N/A | N/A | N/A | N/A | N/A |
| T | -1.95 | 0 | 0 | 0 | -2.40 | N/A | N/A | N/A | N/A | N/A | N/A | N/A |
| V | -1.95 | 0 | 0 | 0 | -1.51 | N/A | N/A | N/A | N/A | N/A | N/A | N/A |
| C | -5.65 | 0 | 0 | 0 | -2.40 | N/A | N/A | N/A | N/A | N/A | N/A | N/A |
| D | -5.65 | 0 | 0 | 0 | -2.40 | N/A | N/A | N/A | N/A | N/A | N/A | N/A |
| E | -5.65 | 0 | 0 | 0 | -2.40 | N/A | N/A | N/A | N/A | N/A | N/A | N/A |
| F | -5.65 | 0 | 0 | 0 | -2.40 | N/A | N/A | N/A | N/A | N/A | N/A | N/A |
| G | -5.65 | 0 | 0 | 0 | -2.40 | N/A | N/A | N/A | N/A | N/A | N/A | N/A |
| K | -5.65 | 0 | 0 | 0 | -2.40 | N/A | N/A | N/A | N/A | N/A | N/A | N/A |
| M | -5.65 | 0 | 0 | 0 | -2.40 | N/A | N/A | N/A | N/A | N/A | N/A | N/A |
| W | -5.65 | 0 | 0 | 0 | -2.40 | N/A | N/A | N/A | N/A | N/A | N/A | N/A |
| Y | -5.65 | 0 | 0 | 0 | -2.40 | N/A | N/A | N/A | N/A | N/A | N/A | N/A |
| Pearson correlation coefficient to W_ji_ | | | N/A | N/A | 0.44 |  | | | | | N/A |  |

| Position | 92CDR3L | | | | | | | | | | | |
| --- | --- | --- | --- | --- | --- | --- | --- | --- | --- | --- | --- | --- |
| Amino Acid | W_ji_ | δW_ji_ | X_ji_ | Y_ji_ | Z_ji_ | x | y | z | a | t | pW_ji_-t | δpW_ji_ |
| F | 4.28 | 1 | 53.79 | -0.87 | -0.24 | 0.09 | 0.18 | 1.19 | -3.69 | 0.47 | 0.19 | 1 |
| Y | 3.56 | 1 | 59.79 | -5.51 | 0.85 | 0.02 | 0.06 | 0.82 | -2.42 | 0.19 | 0.13 | 1 |
| H | 2.97 | 1 | 48.11 | 1.05 | 0.95 | -0.02 | -0.02 | 0.19 | 0.58 | 0.18 | 0.25 | 1 |
| N | 2.97 | 1 | 25.65 | 1.88 | 1.36 | 0.00 | 0.04 | 0.27 | -0.55 | 0.15 | 0.35 | 1 |
| I | 2.35 | 1 | 32.01 | -0.68 | -0.85 | -0.02 | -0.08 | 0.66 | 0.25 | 0.60 | -0.33 | 0 |
| S | 2.09 | 1 | 16.93 | 4.34 | 1.16 | -0.07 | 0.18 | 3.10 | -0.62 | 0.32 | 0.60 | 1 |
| L | 1.23 | 1 | 31.93 | 1.11 | -0.67 | -0.04 | -0.20 | 0.68 | -0.16 | 0.21 | -0.09 | 0 |
| R | -1.24 | 0 | 56.04 | -3.59 | -0.76 | -0.09 | 0.35 | 0.53 | 4.15 | 0.10 | -0.01 | 0 |
| V | -1.25 | 0 | 23.61 | 0.47 | -1.50 | 0.08 | 0.04 | -0.87 | -6.23 | 0.70 | -0.65 | 0 |
| T | -2.76 | 0 | 24.34 | 0.77 | 0.17 | -0.03 | -0.01 | -1.15 | -0.19 | 0.77 | -0.50 | 0 |
| A | -6.10 | 0 | 10.31 | 5.17 | -1.12 | -0.02 | 1.11 | -0.43 | -1.70 | 0.06 | 0.93 | 1 |
| C | -6.10 | 0 | 17.07 | 2.82 | -2.39 | 0.00 | 0.00 | 0.00 | -102.6 | 0.50 | -0.50 | 0 |
| D | -6.10 | 0 | 10.95 | 3.57 | 0.07 | 0.05 | -0.04 | -0.83 | -2.59 | 0.25 | -0.16 | 0 |
| E | -6.10 | 0 | 24.04 | 2.15 | -1.46 | 0.07 | 0.11 | -0.69 | -4.09 | 0.23 | -0.01 | 0 |
| G | -6.10 | 0 | 5.55 | 6.09 | 0.09 | -0.10 | -0.24 | 0.39 | 0.71 | 0.27 | -0.05 | 0 |
| K | -6.10 | 0 | 40.96 | -1.57 | -0.94 | 0.04 | -0.09 | -0.92 | -5.38 | 0.70 | -0.64 | 0 |
| M | -6.10 | 0 | 33.52 | -0.05 | -2.39 | 0.09 | 0.19 | 0.21 | -3.15 | 0.50 | -0.14 | 0 |
| P | -6.10 | 0 | 16.80 | 4.10 | -2.39 | 0.08 | 0.26 | 0.03 | -1.76 | 0.25 | 0.38 | 1 |
| Q | -6.10 | 0 | 33.27 | -1.76 | -0.22 | -0.09 | 0.21 | 2.78 | 3.01 | 0.70 | -0.41 | 0 |
| W | -6.10 | 0 | 76.09 | -0.56 | 0.69 | 0.03 | 0.15 | 0.43 | -3.01 | 0.70 | -0.24 | 0 |
| Pearson correlation coefficient to W_ji_ | | | 0.34 | -0.35 | 0.53 |  | | | | | 0.26 |  |

| Position | 93CDR3L | | | | | | | | | | | |
| --- | --- | --- | --- | --- | --- | --- | --- | --- | --- | --- | --- | --- |
| Amino Acid | W_ji_ | δW_ji_ | X_ji_ | Y_ji_ | Z_ji_ | x | y | z | a | t | pW_ji_-t | δpW_ji_ |
| K | 4.90 | 1 | 27.95 | -1.33 | -2.40 | 0.04 | -0.10 | -0.98 | -5.54 | 0.70 | -0.57 | 0 |
| N | 3.90 | 1 | 12.71 | 0.50 | 0.78 | 0.01 | 0.06 | 0.31 | -0.75 | 0.15 | 0.27 | 1 |
| M | 3.47 | 1 | 22.03 | -1.31 | -0.65 | 0.16 | 0.29 | -1.06 | -8.39 | 0.50 | -0.49 | 0 |
| I | 2.97 | 1 | 12.93 | 1.08 | -1.36 | 0.00 | -0.07 | 0.81 | -0.09 | 0.60 | -0.39 | 0 |
| R | 1.55 | 1 | 42.37 | 1.82 | -1.29 | -0.09 | 0.20 | 1.01 | 4.00 | 0.10 | 0.24 | 1 |
| T | 1.05 | 1 | 6.75 | 2.89 | 0.67 | -0.03 | -0.13 | -1.71 | -0.70 | 0.77 | -0.69 | 0 |
| S | 0.88 | 1 | 0.00 | 0.33 | 1.28 | -0.07 | 0.19 | 3.15 | -0.65 | 0.32 | 0.65 | 1 |
| P | -1.25 | 0 | 4.59 | 2.65 | -1.26 | 0.07 | 0.19 | 0.12 | -1.49 | 0.25 | 0.05 | 1 |
| A | -2.76 | 0 | 0.00 | 0.25 | -1.13 | -0.02 | 0.32 | -0.49 | -1.26 | 0.06 | 0.29 | 1 |
| G | -2.76 | 0 | 0.00 | 0.63 | 0.29 | -0.12 | -0.31 | 0.46 | 1.03 | 0.27 | 0.45 | 1 |
| C | -6.10 | 0 | 6.16 | 0.33 | -2.40 | 0.00 | 0.00 | 0.00 | -102.6 | 0.50 | -0.50 | 0 |
| D | -6.10 | 0 | 5.97 | 0.58 | -0.11 | 0.05 | -0.05 | -0.83 | -2.56 | 0.25 | -0.15 | 0 |
| E | -6.10 | 0 | 15.29 | 1.41 | 0.91 | 0.06 | 0.10 | -0.59 | -4.01 | 0.23 | -0.20 | 0 |
| F | -6.10 | 0 | 44.10 | 0.68 | -1.14 | 0.09 | 0.20 | 1.25 | -3.48 | 0.47 | -0.16 | 0 |
| H | -6.10 | 0 | 35.07 | -0.89 | 1.43 | -0.02 | 0.00 | 0.52 | 1.10 | 0.18 | 0.58 | 1 |
| L | -6.10 | 0 | 19.48 | 1.30 | -1.21 | -0.01 | -0.05 | 0.77 | -0.31 | 0.21 | -0.02 | 0 |
| Q | -6.10 | 0 | 22.65 | 1.60 | -0.58 | -0.07 | 0.53 | 2.87 | 2.23 | 0.70 | -0.22 | 0 |
| V | -6.10 | 0 | 7.05 | 2.22 | -2.40 | 0.08 | 0.03 | -0.91 | -6.28 | 0.70 | -0.67 | 0 |
| W | -6.10 | 0 | 59.21 | -4.76 | -2.40 | 0.03 | 0.11 | 0.36 | -2.75 | 0.70 | -0.63 | 0 |
| Y | -6.10 | 0 | 50.98 | -7.95 | -2.40 | 0.02 | 0.04 | 0.78 | -2.16 | 0.19 | -0.16 | 0 |
| Pearson correlation coefficient to W_ji_ | | | -0.20 | 0.16 | 0.13 |  | | | | | 0.02 |  |

| Position | 94CDR3L | | | | | | | | | | | |
| --- | --- | --- | --- | --- | --- | --- | --- | --- | --- | --- | --- | --- |
| Amino Acid | W_ji_ | δW_ji_ | X_ji_ | Y_ji_ | Z_ji_ | x | y | z | a | t | pW_ji_-t | δpW_ji_ |
| P | 2.92 | 1 | 0.00 | 0.21 | 0.22 | 0.10 | 0.26 | -0.20 | -3.15 | 0.25 | -0.21 | 0 |
| R | 1.55 | 1 | 28.49 | 0.00 | -0.18 | -0.07 | 0.36 | 0.52 | 3.46 | 0.10 | 0.68 | 1 |
| A | 1.53 | 1 | 0.00 | 0.00 | -1.57 | 0.00 | 0.41 | -0.37 | -1.71 | 0.06 | 0.18 | 1 |
| G | 1.05 | 1 | 0.00 | 0.00 | -1.08 | -0.09 | -0.28 | 0.41 | 0.53 | 0.27 | 0.25 | 1 |
| H | 0.50 | 1 | 14.47 | 0.00 | -0.09 | 0.00 | 0.03 | 0.25 | -0.07 | 0.18 | 0.29 | 1 |
| I | 0.50 | 1 | 0.00 | 0.04 | -1.36 | 0.02 | 0.02 | 0.82 | -0.72 | 0.60 | -0.46 | 0 |
| T | 0.46 | 1 | 0.00 | 0.01 | -0.59 | -0.01 | 0.00 | -1.11 | -0.61 | 0.77 | -0.26 | 0 |
| S | 0.01 | 1 | 0.00 | 0.00 | 0.30 | -0.06 | 0.21 | 3.24 | -0.99 | 0.32 | 0.17 | 1 |
| D | -1.22 | 0 | 0.00 | 0.00 | 0.06 | 0.05 | -0.05 | -0.83 | -2.57 | 0.25 | -0.18 | 0 |
| E | -1.22 | 0 | 5.22 | 0.00 | -1.46 | 0.06 | 0.09 | -0.64 | -4.05 | 0.23 | -0.17 | 0 |
| F | -1.22 | 0 | 11.45 | 0.00 | 0.66 | 0.08 | 0.17 | 1.27 | -3.08 | 0.47 | -0.26 | 0 |
| K | -1.22 | 0 | 18.89 | 0.00 | -0.95 | 0.04 | -0.10 | -0.93 | -5.36 | 0.70 | -0.68 | 0 |
| M | -1.22 | 0 | 6.35 | 0.00 | -2.40 | 0.09 | 0.16 | 0.32 | -2.95 | 0.50 | -0.46 | 0 |
| N | -1.22 | 0 | 0.00 | 0.00 | -0.08 | 0.00 | 0.05 | 0.34 | -0.19 | 0.15 | 0.30 | 1 |
| Q | -1.25 | 0 | 6.91 | 0.00 | -2.40 | -0.08 | 0.48 | 2.78 | 2.50 | 0.70 | -0.69 | 0 |
| V | -1.25 | 0 | 0.00 | 0.03 | 0.23 | 0.08 | 0.03 | -0.91 | -6.44 | 0.70 | -0.70 | 0 |
| C | -6.10 | 0 | 0.00 | 0.01 | -2.40 | 0.00 | 0.00 | 0.00 | -102.6 | 0.50 | -0.50 | 0 |
| L | -6.10 | 0 | 0.00 | 0.00 | 0.56 | -0.05 | -0.20 | 1.11 | 1.30 | 0.21 | 0.66 | 1 |
| W | -6.10 | 0 | 19.55 | 0.00 | 1.35 | 0.02 | 0.10 | 0.39 | -2.23 | 0.70 | -0.49 | 0 |
| Y | -6.10 | 0 | 13.44 | 0.17 | 1.62 | 0.00 | 0.03 | 0.76 | -1.06 | 0.19 | 0.36 | 1 |
| Pearson correlation coefficient to W_ji_ | | | -0.11 | 0.04 | -0.25 |  | | | | | 0.06 |  |

| Position | 95CDR3L | | | | | | | | | | | |
| --- | --- | --- | --- | --- | --- | --- | --- | --- | --- | --- | --- | --- |
| Amino Acid | W_ji_ | δW_ji_ | X_ji_ | Y_ji_ | Z_ji_ | x | y | z | a | t | pW_ji_-t | δpW_ji_ |
| P | 3.19 | 1 | 0 | 0 | 2.73 | N/A | N/A | N/A | N/A | N/A | N/A | N/A |
| K | 1.58 | 1 | 0 | 0 | -0.61 | N/A | N/A | N/A | N/A | N/A | N/A | N/A |
| N | 1.58 | 1 | 0 | 0 | -1.19 | N/A | N/A | N/A | N/A | N/A | N/A | N/A |
| S | 1.55 | 1 | 0 | 0 | -1.45 | N/A | N/A | N/A | N/A | N/A | N/A | N/A |
| T | 1.53 | 1 | 0 | 0 | -1.39 | N/A | N/A | N/A | N/A | N/A | N/A | N/A |
| A | 1.05 | 1 | 0 | 0 | -1.11 | N/A | N/A | N/A | N/A | N/A | N/A | N/A |
| F | 0.50 | 1 | 0 | 0 | -2.39 | N/A | N/A | N/A | N/A | N/A | N/A | N/A |
| G | -0.27 | 0 | 0 | 0 | -1.52 | N/A | N/A | N/A | N/A | N/A | N/A | N/A |
| L | -0.55 | 0 | 0 | 0 | -1.19 | N/A | N/A | N/A | N/A | N/A | N/A | N/A |
| R | -0.55 | 0 | 0 | 0 | -1.27 | N/A | N/A | N/A | N/A | N/A | N/A | N/A |
| I | -1.22 | 0 | 0 | 0 | -2.39 | N/A | N/A | N/A | N/A | N/A | N/A | N/A |
| V | -1.25 | 0 | 0 | 0 | -1.50 | N/A | N/A | N/A | N/A | N/A | N/A | N/A |
| C | -6.10 | 0 | 0 | 0 | -2.39 | N/A | N/A | N/A | N/A | N/A | N/A | N/A |
| D | -6.10 | 0 | 0 | 0 | -0.86 | N/A | N/A | N/A | N/A | N/A | N/A | N/A |
| E | -6.10 | 0 | 0 | 0 | -0.21 | N/A | N/A | N/A | N/A | N/A | N/A | N/A |
| H | -6.10 | 0 | 0 | 0 | -2.39 | N/A | N/A | N/A | N/A | N/A | N/A | N/A |
| M | -6.10 | 0 | 0 | 0 | -2.39 | N/A | N/A | N/A | N/A | N/A | N/A | N/A |
| Q | -6.10 | 0 | 0 | 0 | -1.10 | N/A | N/A | N/A | N/A | N/A | N/A | N/A |
| W | -6.10 | 0 | 0 | 0 | -2.39 | N/A | N/A | N/A | N/A | N/A | N/A | N/A |
| Y | -6.10 | 0 | 0 | 0 | -2.39 | N/A | N/A | N/A | N/A | N/A | N/A | N/A |
| Pearson correlation coefficient to W_ji_ | | | N/A | N/A | 0.44 |  | | | | | N/A |  |

| Position | 96CDR3L | | | | | | | | | | | |
| --- | --- | --- | --- | --- | --- | --- | --- | --- | --- | --- | --- | --- |
| Amino Acid | W_ji_ | δW_ji_ | X_ji_ | Y_ji_ | Z_ji_ | x | y | z | a | t | pW_ji_-t | δpW_ji_ |
| H | 5.65 | 1 | 0 | 0 | -0.69 | N/A | N/A | N/A | N/A | N/A | N/A | N/A |
| Y | 2.44 | 1 | 0 | 0 | 1.40 | N/A | N/A | N/A | N/A | N/A | N/A | N/A |
| F | 2.35 | 1 | 0 | 0 | 0.87 | N/A | N/A | N/A | N/A | N/A | N/A | N/A |
| P | 1.95 | 1 | 0 | 0 | 1.16 | N/A | N/A | N/A | N/A | N/A | N/A | N/A |
| M | 1.58 | 1 | 0 | 0 | 0.33 | N/A | N/A | N/A | N/A | N/A | N/A | N/A |
| E | 0.50 | 1 | 0 | 0 | -2.40 | N/A | N/A | N/A | N/A | N/A | N/A | N/A |
| N | 0.50 | 1 | 0 | 0 | -2.40 | N/A | N/A | N/A | N/A | N/A | N/A | N/A |
| A | 0.46 | 1 | 0 | 0 | -1.57 | N/A | N/A | N/A | N/A | N/A | N/A | N/A |
| G | -0.27 | 0 | 0 | 0 | -1.54 | N/A | N/A | N/A | N/A | N/A | N/A | N/A |
| D | -1.22 | 0 | 0 | 0 | -2.40 | N/A | N/A | N/A | N/A | N/A | N/A | N/A |
| S | -1.24 | 0 | 0 | 0 | -1.47 | N/A | N/A | N/A | N/A | N/A | N/A | N/A |
| L | -2.16 | 0 | 0 | 0 | 0.91 | N/A | N/A | N/A | N/A | N/A | N/A | N/A |
| R | -2.16 | 0 | 0 | 0 | 0.76 | N/A | N/A | N/A | N/A | N/A | N/A | N/A |
| Q | -2.76 | 0 | 0 | 0 | -1.12 | N/A | N/A | N/A | N/A | N/A | N/A | N/A |
| T | -2.76 | 0 | 0 | 0 | -0.91 | N/A | N/A | N/A | N/A | N/A | N/A | N/A |
| V | -2.76 | 0 | 0 | 0 | -0.50 | N/A | N/A | N/A | N/A | N/A | N/A | N/A |
| C | -6.10 | 0 | 0 | 0 | -0.55 | N/A | N/A | N/A | N/A | N/A | N/A | N/A |
| I | -6.10 | 0 | 0 | 0 | -2.40 | N/A | N/A | N/A | N/A | N/A | N/A | N/A |
| K | -6.10 | 0 | 0 | 0 | -1.43 | N/A | N/A | N/A | N/A | N/A | N/A | N/A |
| W | -6.10 | 0 | 0 | 0 | 1.63 | N/A | N/A | N/A | N/A | N/A | N/A | N/A |
| Pearson correlation coefficient to W_ji_ | | | N/A | N/A | 0.13 |  | | | | | N/A |  |

| Position | 29CDR1H | | | | | | | | | | | |
| --- | --- | --- | --- | --- | --- | --- | --- | --- | --- | --- | --- | --- |
| Amino Acid | W_ji_ | δW_ji_ | X_ji_ | Y_ji_ | Z_ji_ | x | y | z | a | t | pW_ji_-t | δpW_ji_ |
| I | 4.90 | 1 | 0 | 0 | 1.72 | N/A | N/A | N/A | N/A | N/A | N/A | N/A |
| L | 4.21 | 1 | 0 | 0 | -0.49 | N/A | N/A | N/A | N/A | N/A | N/A | N/A |
| M | 2.97 | 1 | 0 | 0 | -2.40 | N/A | N/A | N/A | N/A | N/A | N/A | N/A |
| V | 2.92 | 1 | 0 | 0 | -0.73 | N/A | N/A | N/A | N/A | N/A | N/A | N/A |
| F | 2.18 | 1 | 0 | 0 | 2.58 | N/A | N/A | N/A | N/A | N/A | N/A | N/A |
| A | -5.81 | 0 | 0 | 0 | -2.40 | N/A | N/A | N/A | N/A | N/A | N/A | N/A |
| C | -5.81 | 0 | 0 | 0 | -2.40 | N/A | N/A | N/A | N/A | N/A | N/A | N/A |
| D | -5.81 | 0 | 0 | 0 | -2.40 | N/A | N/A | N/A | N/A | N/A | N/A | N/A |
| E | -5.81 | 0 | 0 | 0 | -2.40 | N/A | N/A | N/A | N/A | N/A | N/A | N/A |
| G | -5.81 | 0 | 0 | 0 | -2.40 | N/A | N/A | N/A | N/A | N/A | N/A | N/A |
| H | -5.81 | 0 | 0 | 0 | -2.40 | N/A | N/A | N/A | N/A | N/A | N/A | N/A |
| K | -5.81 | 0 | 0 | 0 | -2.40 | N/A | N/A | N/A | N/A | N/A | N/A | N/A |
| N | -5.81 | 0 | 0 | 0 | -2.40 | N/A | N/A | N/A | N/A | N/A | N/A | N/A |
| P | -5.81 | 0 | 0 | 0 | -2.40 | N/A | N/A | N/A | N/A | N/A | N/A | N/A |
| Q | -5.81 | 0 | 0 | 0 | -2.40 | N/A | N/A | N/A | N/A | N/A | N/A | N/A |
| R | -5.81 | 0 | 0 | 0 | -2.40 | N/A | N/A | N/A | N/A | N/A | N/A | N/A |
| S | -5.81 | 0 | 0 | 0 | -0.99 | N/A | N/A | N/A | N/A | N/A | N/A | N/A |
| T | -5.81 | 0 | 0 | 0 | -2.40 | N/A | N/A | N/A | N/A | N/A | N/A | N/A |
| W | -5.81 | 0 | 0 | 0 | -2.40 | N/A | N/A | N/A | N/A | N/A | N/A | N/A |
| Y | -5.81 | 0 | 0 | 0 | -2.40 | N/A | N/A | N/A | N/A | N/A | N/A | N/A |
| Pearson correlation coefficient to W_ji_ | | | N/A | N/A | 0.74 |  | | | | | N/A |  |

| Position | 30CDR1H | | | | | | | | | | | |
| --- | --- | --- | --- | --- | --- | --- | --- | --- | --- | --- | --- | --- |
| Amino Acid | W_ji_ | δW_ji_ | X_ji_ | Y_ji_ | Z_ji_ | x | y | z | a | t | pW_ji_-t | δpW_ji_ |
| D | 5.23 | 1 | 25.52 | 0.25 | -2.39 | 0.04 | -0.06 | -0.58 | -2.51 | 0.25 | 0.22 | 1 |
| E | 4.90 | 1 | 37.85 | 0.79 | -1.46 | 0.05 | 0.05 | -0.29 | -3.67 | 0.23 | 0.01 | 1 |
| N | 2.97 | 1 | 24.80 | 0.31 | -0.67 | 0.00 | 0.04 | 0.53 | -0.68 | 0.15 | 0.14 | 1 |
| S | 1.84 | 1 | 18.87 | 1.06 | 1.25 | -0.07 | 0.18 | 3.05 | -0.62 | 0.32 | 0.56 | 1 |
| Y | 1.19 | 1 | 71.02 | -0.10 | -1.08 | 0.01 | 0.00 | 1.23 | -2.66 | 0.19 | -0.15 | 0 |
| R | 0.03 | 1 | 58.14 | -0.13 | -0.17 | -0.14 | 0.24 | 0.58 | 6.38 | 0.10 | 0.02 | 1 |
| K | -0.65 | 0 | 45.52 | 0.71 | 1.12 | 0.04 | -0.10 | -0.94 | -5.48 | 0.70 | -0.69 | 0 |
| G | -0.69 | 0 | 13.37 | 1.60 | -1.07 | -0.10 | -0.29 | 0.32 | 0.69 | 0.27 | -0.07 | 0 |
| A | -2.23 | 0 | 19.18 | -0.06 | -1.12 | -0.01 | 0.33 | -0.49 | -1.40 | 0.06 | 0.19 | 1 |
| Q | -2.23 | 0 | 39.05 | 1.02 | -2.39 | -0.07 | 0.50 | 2.83 | 2.46 | 0.70 | -0.70 | 0 |
| T | -2.23 | 0 | 25.81 | 1.55 | 1.90 | -0.03 | -0.03 | -1.18 | -0.22 | 0.77 | -0.73 | 0 |
| L | -3.00 | 0 | 39.49 | -0.28 | -2.39 | 0.00 | -0.05 | 0.77 | -0.42 | 0.21 | -0.13 | 0 |
| C | -5.81 | 0 | 26.09 | -0.87 | -2.39 | 0.00 | 0.00 | 0.00 | -102.6 | 0.50 | -0.50 | 0 |
| F | -5.81 | 0 | 63.71 | -0.01 | -2.39 | 0.10 | 0.22 | 1.21 | -3.88 | 0.47 | -0.11 | 0 |
| H | -5.81 | 0 | 48.44 | -0.25 | -2.39 | -0.01 | 0.03 | 0.22 | 0.43 | 0.18 | 0.18 | 1 |
| I | -5.81 | 0 | 38.89 | -1.35 | -1.35 | -0.01 | -0.03 | 0.69 | 0.24 | 0.60 | -0.33 | 0 |
| M | -5.81 | 0 | 40.35 | -1.10 | -2.39 | 0.10 | 0.20 | 0.19 | -3.39 | 0.50 | -0.02 | 0 |
| P | -5.81 | 0 | 24.66 | 0.01 | -2.39 | 0.07 | 0.20 | 0.04 | -1.71 | 0.25 | 0.24 | 1 |
| V | -5.81 | 0 | 32.30 | 0.22 | -2.39 | 0.08 | 0.05 | -1.01 | -6.55 | 0.70 | -0.50 | 0 |
| W | -5.81 | 0 | 86.35 | -7.19 | -2.39 | 0.03 | 0.11 | 0.35 | -2.86 | 0.70 | -0.58 | 0 |
| Pearson correlation coefficient to W_ji_ | | | -0.23 | 0.42 | 0.40 |  | | | | | 0.35 |  |

| Position | 31CDR1H | | | | | | | | | | | |
| --- | --- | --- | --- | --- | --- | --- | --- | --- | --- | --- | --- | --- |
| Amino Acid | W_ji_ | δW_ji_ | X_ji_ | Y_ji_ | Z_ji_ | x | y | z | a | t | pW_ji_-t | δpW_ji_ |
| D | 8.02 | 1 | 44.55 | -7.29 | 1.86 | 0.06 | 0.00 | -1.42 | -3.46 | 0.25 | -0.22 | 0 |
| N | 5.53 | 1 | 45.10 | -10.50 | 1.03 | 0.01 | 0.17 | 0.24 | -0.58 | 0.15 | 0.01 | 1 |
| E | 2.97 | 1 | 51.19 | -12.93 | -0.99 | 0.09 | 0.51 | -0.39 | -4.71 | 0.23 | -0.23 | 0 |
| H | 2.18 | 1 | 58.55 | -15.39 | -0.69 | 0.00 | 0.18 | 0.23 | -0.01 | 0.18 | -0.12 | 0 |
| S | -3.00 | 0 | 29.47 | -11.66 | 1.35 | -0.08 | 0.05 | 3.36 | -0.54 | 0.32 | 0.41 | 1 |
| A | -5.81 | 0 | 22.60 | -5.94 | -0.82 | -0.02 | 0.32 | -0.50 | -1.42 | 0.06 | -0.02 | 0 |
| C | -5.81 | 0 | 30.32 | -11.32 | -2.40 | 0.00 | 0.00 | 0.00 | -102.6 | 0.50 | -0.50 | 0 |
| F | -5.81 | 0 | 66.65 | -11.64 | -1.14 | 0.09 | 0.15 | 1.26 | -3.52 | 0.47 | -0.14 | 0 |
| G | -5.81 | 0 | 15.08 | 0.36 | -1.08 | -0.10 | -0.30 | 0.31 | 0.69 | 0.27 | -0.04 | 0 |
| I | -5.81 | 0 | 44.49 | -14.78 | -2.40 | -0.03 | -0.17 | 0.69 | 0.59 | 0.60 | -0.08 | 0 |
| K | -5.81 | 0 | 44.19 | -10.54 | -1.43 | 0.03 | -0.24 | -1.27 | -5.71 | 0.70 | -0.24 | 0 |
| L | -5.81 | 0 | 43.55 | -9.75 | -2.40 | -0.01 | -0.10 | 0.71 | -0.28 | 0.21 | -0.04 | 0 |
| M | -5.81 | 0 | 44.77 | -13.94 | -2.40 | 0.09 | 0.13 | 0.33 | -2.87 | 0.50 | -0.35 | 0 |
| P | -5.81 | 0 | 35.73 | -9.09 | -2.40 | 0.06 | 0.12 | 0.04 | -1.59 | 0.25 | 0.13 | 1 |
| Q | -5.81 | 0 | 51.71 | -13.68 | -2.40 | -0.08 | 0.50 | 2.84 | 2.47 | 0.70 | -0.70 | 0 |
| R | -5.81 | 0 | 64.87 | -10.59 | -1.29 | -0.09 | 0.37 | 0.56 | 4.26 | 0.10 | -0.10 | 0 |
| T | -5.81 | 0 | 37.28 | -9.95 | 0.50 | -0.03 | -0.07 | -1.19 | -0.15 | 0.77 | -0.53 | 0 |
| V | -5.81 | 0 | 37.32 | -13.92 | -2.40 | 0.06 | -0.11 | -1.47 | -7.12 | 0.70 | -0.13 | 0 |
| W | -5.81 | 0 | 87.39 | -21.68 | -2.40 | 0.03 | 0.10 | 0.37 | -2.84 | 0.70 | -0.67 | 0 |
| Y | -5.81 | 0 | 74.47 | -10.60 | -2.40 | 0.02 | 0.04 | 0.78 | -2.19 | 0.19 | -0.14 | 0 |
| Pearson correlation coefficient to W_ji_ | | | 0.03 | 0.04 | 0.69 |  | | | | | 0.16 |  |

| Position | 32CDR1H | | | | | | | | | | | |
| --- | --- | --- | --- | --- | --- | --- | --- | --- | --- | --- | --- | --- |
| Amino Acid | W_ji_ | δW_ji_ | X_ji_ | Y_ji_ | Z_ji_ | x | y | z | a | t | pW_ji_-t | δpW_ji_ |
| Y | 8.34 | 1 | 65.46 | -1.25 | 2.67 | 0.01 | 0.03 | 0.70 | -1.90 | 0.19 | 0.52 | 1 |
| F | 6.86 | 1 | 55.99 | -1.48 | 0.97 | 0.09 | 0.19 | 1.22 | -3.67 | 0.47 | 0.44 | 1 |
| A | -5.81 | 0 | 18.88 | -0.17 | -1.57 | -0.01 | 0.32 | -0.55 | -1.45 | 0.06 | 0.23 | 1 |
| C | -5.81 | 0 | 24.78 | -0.08 | -2.40 | 0.00 | 0.00 | 0.00 | -102.6 | 0.50 | -0.50 | 0 |
| D | -5.81 | 0 | 24.11 | 0.29 | -0.30 | 0.05 | -0.04 | -0.84 | -2.59 | 0.25 | -0.01 | 0 |
| E | -5.81 | 0 | 24.97 | -0.19 | -0.99 | 0.06 | 0.10 | -0.62 | -3.99 | 0.23 | -0.09 | 0 |
| G | -5.81 | 0 | 10.42 | 1.11 | -1.54 | -0.10 | -0.30 | 0.29 | 0.70 | 0.27 | -0.02 | 0 |
| H | -5.81 | 0 | 43.80 | -0.77 | 0.55 | -0.01 | 0.02 | 0.39 | 0.68 | 0.18 | 0.41 | 1 |
| I | -5.81 | 0 | 38.23 | -0.88 | -1.36 | -0.01 | -0.03 | 0.69 | 0.23 | 0.60 | -0.33 | 0 |
| K | -5.81 | 0 | 43.59 | -1.88 | -2.40 | 0.05 | -0.10 | -1.17 | -5.97 | 0.70 | -0.42 | 0 |
| L | -5.81 | 0 | 38.13 | -0.50 | -1.64 | 0.00 | -0.04 | 0.79 | -0.43 | 0.21 | -0.07 | 0 |
| M | -5.81 | 0 | 39.45 | -1.43 | -2.40 | 0.10 | 0.19 | 0.20 | -3.30 | 0.50 | -0.07 | 0 |
| N | -5.81 | 0 | 32.16 | -0.38 | -0.33 | 0.01 | 0.07 | 0.31 | -0.47 | 0.15 | 0.27 | 1 |
| P | -5.81 | 0 | 27.46 | -0.32 | -2.40 | 0.07 | 0.20 | 0.03 | -1.74 | 0.25 | 0.28 | 1 |
| Q | -5.81 | 0 | 45.36 | -1.06 | -2.40 | -0.08 | 0.50 | 2.84 | 2.47 | 0.70 | -0.70 | 0 |
| R | -5.81 | 0 | 61.13 | -3.64 | -2.40 | -0.09 | 0.37 | 0.53 | 4.20 | 0.10 | -0.08 | 0 |
| S | -5.81 | 0 | 16.74 | 0.34 | -0.24 | -0.07 | 0.19 | 3.09 | -0.58 | 0.32 | -0.24 | 0 |
| T | -5.81 | 0 | 24.09 | 0.12 | 0.40 | -0.03 | -0.02 | -1.15 | -0.19 | 0.77 | -0.55 | 0 |
| V | -5.81 | 0 | 29.31 | -0.50 | -2.40 | 0.08 | 0.04 | -0.99 | -6.41 | 0.70 | -0.55 | 0 |
| W | -5.81 | 0 | 66.34 | -1.84 | -2.40 | 0.03 | 0.11 | 0.35 | -2.76 | 0.70 | -0.58 | 0 |
| Pearson correlation coefficient to W_ji_ | | | 0.51 | -0.21 | 0.71 |  | | | | | 0.53 |  |

| Position | 33CDR1H | | | | | | | | | | | |
| --- | --- | --- | --- | --- | --- | --- | --- | --- | --- | --- | --- | --- |
| Amino Acid | W_ji_ | δW_ji_ | X_ji_ | Y_ji_ | Z_ji_ | x | y | z | a | t | pW_ji_-t | δpW_ji_ |
| W | 9.1 | 1 | 65.42 | -5.50 | 2.83 | 0.03 | 0.16 | 0.00 | -3.60 | 0.70 | -0.61 | 0 |
| F | 4.09 | 1 | 36.50 | 0.15 | -1.14 | 0.15 | 0.25 | 1.70 | -6.40 | 0.47 | -0.42 | 0 |
| L | -3.00 | 0 | 18.81 | 0.31 | -2.40 | -0.01 | -0.06 | 0.75 | -0.33 | 0.21 | -0.12 | 0 |
| A | -5.81 | 0 | 0.00 | 0.03 | -0.24 | -0.02 | 0.33 | -0.40 | -1.20 | 0.06 | 0.19 | 1 |
| C | -5.81 | 0 | 0.00 | 0.06 | -0.55 | 0.00 | 0.00 | 0.00 | -102.6 | 0.50 | -0.50 | 0 |
| D | -5.81 | 0 | 6.39 | 0.26 | -0.88 | 0.04 | -0.05 | -0.85 | -2.50 | 0.25 | -0.06 | 0 |
| E | -5.81 | 0 | 13.43 | 0.13 | -1.46 | 0.06 | 0.10 | -0.65 | -4.04 | 0.23 | -0.13 | 0 |
| G | -5.81 | 0 | 0.00 | 0.20 | -0.18 | -0.12 | -0.34 | 0.38 | 1.01 | 0.27 | 0.44 | 1 |
| H | -5.81 | 0 | 34.43 | -1.64 | -0.69 | -0.02 | 0.01 | 0.30 | 0.79 | 0.18 | 0.32 | 1 |
| I | -5.81 | 0 | 17.04 | 0.12 | -2.40 | -0.02 | -0.04 | 0.63 | 0.32 | 0.60 | -0.41 | 0 |
| K | -5.81 | 0 | 21.40 | -1.65 | -2.40 | 0.03 | -0.11 | -1.01 | -5.05 | 0.70 | -0.56 | 0 |
| M | -5.81 | 0 | 22.05 | -1.06 | -2.40 | 0.09 | 0.16 | 0.29 | -2.92 | 0.50 | -0.37 | 0 |
| N | -5.81 | 0 | 12.87 | 0.25 | -0.33 | 0.00 | 0.06 | 0.32 | -0.32 | 0.15 | 0.26 | 1 |
| P | -5.81 | 0 | 0.00 | 1.13 | -0.74 | 0.06 | 0.18 | 0.13 | -1.48 | 0.25 | -0.05 | 0 |
| Q | -5.81 | 0 | 19.42 | 0.03 | -2.40 | -0.08 | 0.50 | 2.82 | 2.47 | 0.70 | -0.70 | 0 |
| R | -5.81 | 0 | 42.22 | -2.73 | -2.40 | -0.09 | 0.32 | 0.24 | 4.44 | 0.10 | 0.19 | 1 |
| S | -5.81 | 0 | 0.00 | 0.07 | 0.07 | -0.08 | 0.18 | 3.02 | -0.25 | 0.32 | 0.17 | 1 |
| T | -5.81 | 0 | 6.92 | 0.19 | -0.34 | -0.04 | -0.05 | -1.28 | 0.15 | 0.77 | -0.20 | 0 |
| V | -5.81 | 0 | 7.15 | 0.13 | -0.73 | 0.08 | 0.03 | -0.90 | -6.39 | 0.70 | -0.69 | 0 |
| Y | -5.81 | 0 | 40.66 | -5.76 | 2.19 | 0.01 | 0.02 | 0.88 | -1.77 | 0.19 | 0.45 | 1 |
| Pearson correlation coefficient to W_ji_ | | | 0.67 | -0.42 | 0.44 |  | | | | | -0.35 |  |

| Position | 53CDR2H | | | | | | | | | | | |
| --- | --- | --- | --- | --- | --- | --- | --- | --- | --- | --- | --- | --- |
| Amino Acid | W_ji_ | δW_ji_ | X_ji_ | Y_ji_ | Z_ji_ | x | y | z | a | t | pW_ji_-t | δpW_ji_ |
| K | 2.23 | 1 | 63.45 | -6.88 | -0.37 | 0.04 | -0.10 | -0.98 | -5.54 | 0.70 | -0.57 | 0 |
| L | 2.01 | 1 | 53.92 | -10.83 | -2.39 | 0.23 | 3.41 | 6.85 | -4.65 | 0.21 | -0.21 | 0 |
| N | 1.62 | 1 | 55.82 | -9.46 | 0.46 | 0.00 | 0.13 | 0.35 | -0.38 | 0.15 | 0.04 | 1 |
| G | 1.58 | 1 | 27.55 | -4.24 | 0.48 | -0.14 | -0.22 | 0.39 | 0.78 | 0.27 | -0.13 | 0 |
| P | 1.22 | 1 | 47.18 | -4.78 | 1.11 | 0.06 | 0.19 | -0.06 | -1.84 | 0.25 | 0.28 | 1 |
| T | 0.33 | 1 | 46.96 | -4.20 | -0.90 | -0.04 | -0.01 | -1.11 | -0.04 | 0.77 | -0.50 | 0 |
| D | -0.21 | 0 | 57.18 | -10.65 | 0.46 | 0.06 | -0.10 | -0.91 | -2.94 | 0.25 | 0.51 | 1 |
| E | -0.21 | 0 | 64.24 | -12.96 | -0.21 | 0.07 | 0.06 | -0.59 | -4.16 | 0.23 | 0.16 | 1 |
| H | -0.21 | 0 | 72.09 | -10.45 | -0.08 | -0.01 | -0.01 | 0.35 | 0.43 | 0.18 | 0.27 | 1 |
| S | -0.24 | 0 | 41.22 | -3.12 | 1.03 | -0.06 | 0.20 | 3.17 | -0.70 | 0.32 | 0.02 | 1 |
| Q | -0.25 | 0 | 62.81 | -13.09 | -1.10 | -0.08 | 0.50 | 2.84 | 2.47 | 0.70 | -0.70 | 0 |
| V | -0.25 | 0 | 45.63 | -11.71 | -1.50 | 0.09 | -0.01 | -0.86 | -6.47 | 0.70 | -0.47 | 0 |
| F | -1.90 | 0 | 69.47 | -13.67 | -1.12 | 0.09 | 0.13 | 1.26 | -3.50 | 0.47 | -0.11 | 0 |
| I | -1.90 | 0 | 53.41 | -11.12 | -2.39 | -0.01 | -0.07 | 0.68 | 0.28 | 0.60 | -0.39 | 0 |
| M | -1.90 | 0 | 55.28 | -14.50 | -2.39 | 0.09 | 0.11 | 0.35 | -2.94 | 0.50 | -0.14 | 0 |
| W | -1.90 | 0 | 92.21 | -22.94 | 0.70 | 0.03 | 0.08 | 0.39 | -2.83 | 0.70 | -0.57 | 0 |
| A | -1.93 | 0 | 33.08 | -4.58 | -0.80 | -0.01 | 0.32 | -0.49 | -1.43 | 0.06 | -0.01 | 0 |
| R | -4.09 | 0 | 79.28 | -10.86 | -0.42 | -0.09 | 0.37 | 0.56 | 4.27 | 0.10 | -0.10 | 0 |
| C | -6.46 | 0 | 40.97 | -8.97 | -2.39 | 0.00 | 0.00 | 0.00 | -102.6 | 0.50 | -0.50 | 0 |
| Y | -6.46 | 0 | 77.44 | -12.86 | 1.05 | 0.03 | 0.00 | 0.84 | -2.54 | 0.19 | 0.38 | 1 |
| Pearson correlation coefficient to W_ji_ | | | -0.28 | 0.31 | 0.16 |  | | | | | -0.04 |  |

| Position | 54CDR2H | | | | | | | | | | | |
| --- | --- | --- | --- | --- | --- | --- | --- | --- | --- | --- | --- | --- |
| Amino Acid | W_ji_ | δW_ji_ | X_ji_ | Y_ji_ | Z_ji_ | x | y | z | a | t | pW_ji_-t | δpW_ji_ |
| P | 5.53 | 1 | 49.64 | 1.93 | -1.23 | 0.06 | 0.16 | 0.10 | -1.56 | 0.25 | 0.60 | 1 |
| N | 2.23 | 1 | 53.92 | 2.76 | 0.82 | -0.02 | -0.02 | 0.34 | -0.16 | 0.15 | 0.17 | 1 |
| T | 1.90 | 1 | 47.09 | -3.56 | -0.88 | -0.05 | -0.03 | -1.13 | 0.00 | 0.77 | -0.51 | 0 |
| Y | -0.13 | 0 | 85.34 | -2.66 | -2.38 | 0.02 | 0.05 | 0.77 | -2.25 | 0.19 | -0.10 | 0 |
| M | -0.21 | 0 | 55.83 | -10.12 | -2.38 | 0.10 | 0.14 | 0.31 | -3.28 | 0.50 | 0.00 | 0 |
| S | -0.24 | 0 | 40.70 | 1.85 | 0.61 | -0.06 | 0.23 | 3.07 | -0.68 | 0.32 | -0.02 | 0 |
| D | -1.90 | 0 | 54.53 | 0.44 | 1.60 | 0.06 | -0.01 | -0.77 | -2.79 | 0.25 | 0.06 | 1 |
| E | -1.90 | 0 | 62.17 | -0.91 | -0.20 | 0.09 | 0.17 | -0.53 | -4.69 | 0.23 | 0.51 | 1 |
| H | -1.90 | 0 | 71.61 | -5.33 | -2.38 | -0.01 | 0.02 | 0.24 | 0.30 | 0.18 | 0.11 | 1 |
| I | -1.90 | 0 | 54.74 | -6.65 | -2.38 | -0.01 | -0.05 | 0.67 | 0.21 | 0.60 | -0.44 | 0 |
| W | -1.90 | 0 | 100.2 | -10.39 | -0.30 | 0.03 | 0.10 | 0.39 | -3.12 | 0.70 | -0.44 | 0 |
| R | -1.92 | 0 | 78.48 | -9.69 | -1.25 | -0.09 | 0.37 | 0.56 | 4.27 | 0.10 | -0.10 | 0 |
| A | -1.93 | 0 | 33.45 | 1.31 | -0.79 | 0.00 | 0.36 | -0.43 | -1.45 | 0.06 | 0.26 | 1 |
| G | -1.93 | 0 | 26.30 | 3.06 | 1.31 | -0.09 | -0.28 | 0.38 | 0.67 | 0.27 | -0.17 | 0 |
| L | -4.09 | 0 | 55.67 | -4.32 | -2.38 | 0.00 | -0.05 | 0.78 | -0.45 | 0.21 | -0.12 | 0 |
| C | -6.46 | 0 | 40.91 | -3.60 | -2.38 | 0.00 | 0.00 | 0.00 | -102.6 | 0.50 | -0.50 | 0 |
| F | -6.46 | 0 | 78.62 | -6.60 | -2.38 | 0.10 | 0.19 | 1.23 | -4.05 | 0.47 | -0.06 | 0 |
| K | -6.46 | 0 | 62.89 | -10.04 | -0.59 | 0.05 | -0.14 | -1.03 | -6.26 | 0.70 | -0.40 | 0 |
| Q | -6.46 | 0 | 62.62 | -8.77 | -2.38 | -0.08 | 0.50 | 2.84 | 2.47 | 0.70 | -0.70 | 0 |
| V | -6.46 | 0 | 47.17 | -4.07 | -1.48 | 0.10 | 0.05 | -0.83 | -6.76 | 0.70 | -0.43 | 0 |
| Pearson correlation coefficient to W_ji_ | | | -0.10 | 0.47 | 0.31 |  | | | | | 0.57 |  |

| Position | 55CDR2H | | | | | | | | | | | |
| --- | --- | --- | --- | --- | --- | --- | --- | --- | --- | --- | --- | --- |
| Amino Acid | W_ji_ | δW_ji_ | X_ji_ | Y_ji_ | Z_ji_ | x | y | z | a | t | pW_ji_-t | δpW_ji_ |
| P | 3.11 | 1 | 51.51 | -4.32 | -1.26 | 0.06 | 0.19 | 0.11 | -1.56 | 0.25 | 0.42 | 1 |
| D | 2.23 | 1 | 37.91 | -0.04 | -1.38 | 0.03 | -0.08 | -0.67 | -2.39 | 0.25 | 0.20 | 1 |
| N | 2.23 | 1 | 42.35 | 0.00 | -1.21 | -0.01 | 0.01 | 0.69 | -0.44 | 0.15 | 0.01 | 1 |
| G | 2.18 | 1 | 21.03 | -2.98 | 2.17 | -0.11 | -0.30 | 0.12 | 0.60 | 0.27 | 0.08 | 1 |
| H | 1.62 | 1 | 64.47 | -3.72 | -2.40 | -0.04 | -0.05 | 0.53 | 1.40 | 0.18 | -0.07 | 0 |
| I | 1.62 | 1 | 53.74 | -4.44 | -2.40 | -0.05 | -0.15 | 1.10 | 1.28 | 0.60 | -0.56 | 0 |
| F | 0.85 | 1 | 76.75 | -4.36 | -2.40 | 0.08 | 0.19 | 1.40 | -3.17 | 0.47 | -0.21 | 0 |
| M | 0.85 | 1 | 50.87 | -7.05 | -2.40 | 0.08 | 0.18 | 0.44 | -2.63 | 0.50 | -0.17 | 0 |
| Y | -0.13 | 0 | 83.62 | -3.85 | -2.40 | 0.02 | 0.05 | 0.77 | -2.23 | 0.19 | -0.11 | 0 |
| K | -0.21 | 0 | 57.76 | -3.72 | -0.38 | 0.04 | -0.08 | -0.90 | -5.54 | 0.70 | -0.61 | 0 |
| A | -0.25 | 0 | 28.37 | 0.45 | -1.57 | -0.01 | 0.33 | -0.55 | -1.53 | 0.06 | 0.28 | 1 |
| V | -0.97 | 0 | 43.19 | -0.58 | -2.40 | 0.13 | 0.09 | -1.44 | -8.89 | 0.70 | -0.13 | 0 |
| L | -1.24 | 0 | 53.36 | -4.62 | -1.64 | 0.00 | -0.05 | 0.80 | -0.47 | 0.21 | -0.05 | 0 |
| W | -1.90 | 0 | 99.01 | -7.90 | -2.40 | 0.03 | 0.11 | 0.35 | -2.98 | 0.70 | -0.55 | 0 |
| R | -1.92 | 0 | 75.42 | -3.70 | -0.18 | -0.09 | 0.37 | 0.56 | 4.16 | 0.10 | -0.08 | 0 |
| S | -2.81 | 0 | 37.19 | 0.11 | 0.57 | -0.07 | 0.21 | 3.07 | -0.64 | 0.32 | -0.10 | 0 |
| Q | -3.39 | 0 | 52.96 | -1.61 | -1.12 | -0.07 | 0.50 | 2.82 | 2.43 | 0.70 | -0.70 | 0 |
| C | -6.46 | 0 | 39.05 | -3.54 | -0.55 | 0.00 | 0.00 | 0.00 | -102.6 | 0.50 | -0.50 | 0 |
| E | -6.46 | 0 | 52.84 | -1.28 | -0.99 | 0.09 | 0.14 | -0.92 | -4.98 | 0.23 | 0.36 | 1 |
| T | -6.46 | 0 | 40.66 | 0.49 | -1.40 | -0.01 | 0.03 | -1.44 | -0.48 | 0.77 | -0.01 | 0 |
| Pearson correlation coefficient to W_ji_ | | | 0.00 | -0.22 | -0.12 |  | | | | | 0.19 |  |

| Position | 56CDR2H | | | | | | | | | | | |
| --- | --- | --- | --- | --- | --- | --- | --- | --- | --- | --- | --- | --- |
| Amino Acid | W_ji_ | δW_ji_ | X_ji_ | Y_ji_ | Z_ji_ | x | y | z | a | t | pW_ji_-t | δpW_ji_ |
| P | 5.53 | 1 | 43.47 | -6.29 | -1.27 | 0.07 | 0.24 | 0.12 | -1.69 | 0.25 | 0.14 | 1 |
| D | 2.73 | 1 | 35.29 | -11.62 | -0.31 | 0.07 | 0.12 | -0.81 | -3.24 | 0.25 | -0.12 | 0 |
| I | 1.62 | 1 | 45.91 | -7.81 | -1.36 | -0.01 | 0.06 | 0.63 | 0.13 | 0.60 | -0.45 | 0 |
| V | 0.33 | 1 | 37.90 | -7.51 | -1.52 | 0.08 | 0.02 | -0.89 | -6.44 | 0.70 | -0.59 | 0 |
| S | 0.16 | 1 | 27.26 | -8.67 | 1.27 | -0.07 | 0.51 | 2.83 | -0.88 | 0.32 | -0.29 | 0 |
| H | -0.21 | 0 | 60.95 | -12.02 | -2.40 | -0.01 | -0.01 | 0.25 | 0.54 | 0.18 | 0.14 | 1 |
| C | -1.90 | 0 | 29.93 | -8.12 | -2.40 | 0.00 | 0.00 | 0.00 | -102.6 | 0.50 | -0.50 | 0 |
| F | -1.90 | 0 | 69.65 | -5.28 | -2.40 | 0.09 | 0.19 | 1.22 | -3.75 | 0.47 | -0.23 | 0 |
| M | -1.90 | 0 | 49.13 | -7.02 | -2.40 | 0.09 | 0.16 | 0.27 | -3.23 | 0.50 | -0.08 | 0 |
| N | -1.90 | 0 | 36.94 | -11.23 | -1.21 | 0.00 | 0.03 | 0.29 | -0.41 | 0.15 | 0.13 | 1 |
| L | -1.92 | 0 | 45.36 | -3.56 | -1.22 | 0.00 | -0.05 | 0.81 | -0.44 | 0.21 | 0.00 | 0 |
| R | -1.92 | 0 | 72.02 | -11.98 | 0.75 | -0.09 | 0.37 | 0.56 | 4.26 | 0.10 | -0.10 | 0 |
| G | -1.93 | 0 | 14.68 | -4.15 | 0.94 | -0.11 | -0.49 | 0.44 | 0.98 | 0.27 | 0.59 | 1 |
| Q | -3.39 | 0 | 51.58 | -11.88 | -0.23 | -0.08 | 0.50 | 2.84 | 2.47 | 0.70 | -0.70 | 0 |
| A | -6.46 | 0 | 22.33 | -5.63 | -0.83 | -0.02 | 0.32 | -0.50 | -1.42 | 0.06 | -0.02 | 0 |
| E | -6.46 | 0 | 43.07 | -11.49 | -1.00 | 0.06 | 0.07 | -0.68 | -4.10 | 0.23 | -0.05 | 0 |
| K | -6.46 | 0 | 52.75 | -9.97 | 0.76 | 0.04 | -0.10 | -0.90 | -5.37 | 0.70 | -0.65 | 0 |
| T | -6.46 | 0 | 29.97 | -8.77 | 0.66 | -0.03 | -0.07 | -1.18 | -0.12 | 0.77 | -0.55 | 0 |
| W | -6.46 | 0 | 91.07 | -12.77 | -2.40 | 0.03 | 0.10 | 0.36 | -2.86 | 0.70 | -0.62 | 0 |
| Y | -6.46 | 0 | 69.03 | -2.13 | -1.09 | 0.02 | 0.05 | 0.76 | -2.19 | 0.19 | -0.02 | 0 |
| Pearson correlation coefficient to W_ji_ | | | -0.20 | 0.02 | -0.11 |  | | | | | 0.25 |  |

| Position | 57CDR2H | | | | | | | | | | | |
| --- | --- | --- | --- | --- | --- | --- | --- | --- | --- | --- | --- | --- |
| Amino Acid | W_ji_ | δW_ji_ | X_ji_ | Y_ji_ | Z_ji_ | x | y | z | a | t | pW_ji_-t | δpW_ji_ |
| P | 4.25 | 1 | 18.46 | 0.48 | -1.26 | 0.07 | 0.18 | 0.09 | -1.87 | 0.25 | 0.11 | 1 |
| A | 1.90 | 1 | 24.01 | 0.55 | -1.13 | -0.04 | 0.36 | -0.55 | -1.50 | 0.06 | 0.11 | 1 |
| S | 1.10 | 1 | 30.69 | -0.88 | 0.90 | -0.09 | 0.17 | 3.23 | -0.62 | 0.32 | 0.01 | 1 |
| D | 0.85 | 1 | 36.83 | 0.15 | -0.30 | 0.03 | -0.10 | -0.83 | -2.56 | 0.25 | 0.00 | 1 |
| E | 0.85 | 1 | 44.22 | -1.61 | -0.43 | 0.08 | 0.07 | -1.19 | -5.36 | 0.23 | -0.07 | 0 |
| F | 0.85 | 1 | 67.45 | 0.48 | 0.77 | 0.09 | 0.19 | 1.26 | -3.70 | 0.47 | 0.50 | 1 |
| H | 0.85 | 1 | 49.05 | -1.08 | -0.09 | -0.02 | -0.01 | 0.25 | 0.60 | 0.18 | 0.23 | 1 |
| K | -0.21 | 0 | 50.68 | -0.53 | -0.19 | 0.04 | -0.09 | -0.91 | -5.44 | 0.70 | -0.66 | 0 |
| N | -0.21 | 0 | 37.41 | -0.87 | 0.95 | 0.01 | 0.07 | 0.43 | -0.55 | 0.15 | 0.40 | 1 |
| L | -0.70 | 0 | 42.71 | 0.09 | -0.91 | 0.01 | -0.01 | 0.86 | -0.55 | 0.21 | 0.04 | 1 |
| Y | -1.82 | 0 | 71.80 | 0.19 | 1.51 | 0.05 | 0.12 | 1.06 | -3.51 | 0.19 | 0.63 | 1 |
| I | -1.90 | 0 | 36.34 | -0.15 | -0.52 | -0.01 | -0.02 | 0.77 | 0.29 | 0.60 | -0.18 | 0 |
| M | -1.90 | 0 | 44.53 | -0.23 | -0.65 | 0.15 | 0.30 | 1.49 | -2.42 | 0.50 | 0.47 | 1 |
| Q | -1.93 | 0 | 51.13 | -0.36 | 0.03 | 0.06 | 1.20 | 4.71 | -0.76 | 0.70 | 0.18 | 1 |
| T | -1.93 | 0 | 29.54 | 0.68 | 0.16 | -0.02 | -0.01 | -1.15 | -0.23 | 0.77 | -0.52 | 0 |
| V | -3.39 | 0 | 30.36 | 0.26 | -2.40 | 0.08 | 0.04 | -1.00 | -6.46 | 0.70 | -0.53 | 0 |
| R | -4.09 | 0 | 56.22 | -0.35 | 0.02 | -0.08 | 0.41 | 0.64 | 3.91 | 0.10 | 0.28 | 1 |
| C | -6.46 | 0 | 24.15 | -0.43 | -0.55 | 0.00 | 0.00 | 0.00 | -102.6 | 0.50 | -0.50 | 0 |
| G | -6.46 | 0 | 17.45 | 0.73 | -2.40 | -0.10 | -0.31 | 0.29 | 0.67 | 0.27 | -0.14 | 0 |
| W | -6.46 | 0 | 90.28 | -0.42 | 0.29 | 0.05 | 0.19 | 0.46 | -3.70 | 0.70 | -0.05 | 0 |
| Pearson correlation coefficient to W_ji_ | | | -0.20 | -0.08 | 0.18 |  | | | | | 0.29 |  |

| Position | 101CDR3H | | | | | | | | | | | |
| --- | --- | --- | --- | --- | --- | --- | --- | --- | --- | --- | --- | --- |
| Amino Acid | W_ji_ | δW_ji_ | X_ji_ | Y_ji_ | Z_ji_ | x | y | z | a | t | pW_ji_-t | δpW_ji_ |
| F | 7.27 | 1 | 77.96 | -6.48 | 0.55 | 0.09 | 0.19 | 1.25 | -3.65 | 0.47 | 0.48 | 1 |
| W | 5.42 | 1 | 101.1 | -8.64 | -1.82 | 0.01 | 0.16 | 0.53 | -2.00 | 0.70 | -0.65 | 0 |
| H | 3.90 | 1 | 67.25 | -8.73 | 0.45 | -0.02 | 0.03 | 0.18 | 0.66 | 0.18 | 0.12 | 1 |
| M | 1.58 | 1 | 55.71 | -6.04 | -1.82 | 0.08 | 0.15 | 0.22 | -2.82 | 0.50 | 0.02 | 1 |
| L | -0.55 | 0 | 54.96 | -7.42 | -0.66 | 0.00 | -0.08 | 0.94 | -0.48 | 0.21 | 0.20 | 1 |
| S | -2.16 | 0 | 40.20 | -1.48 | 0.11 | -0.07 | 0.19 | 3.16 | -0.63 | 0.32 | -0.29 | 0 |
| A | -2.76 | 0 | 33.26 | -0.34 | 0.30 | -0.01 | 0.34 | -0.42 | -1.37 | 0.06 | 0.06 | 1 |
| G | -2.76 | 0 | 26.31 | 0.03 | 0.01 | -0.09 | -0.30 | 0.34 | 0.67 | 0.27 | -0.13 | 0 |
| C | -6.10 | 0 | 40.36 | -2.62 | -1.82 | 0.00 | 0.00 | 0.00 | -102.6 | 0.50 | -0.50 | 0 |
| D | -6.10 | 0 | 55.02 | -4.20 | 0.60 | 0.06 | -0.02 | -0.85 | -2.95 | 0.25 | 0.30 | 1 |
| E | -6.10 | 0 | 63.41 | -6.38 | -0.45 | 0.08 | 0.10 | -0.71 | -4.65 | 0.23 | 0.31 | 1 |
| I | -6.10 | 0 | 54.92 | -6.10 | -0.31 | 0.00 | -0.06 | 0.93 | 0.29 | 0.60 | -0.02 | 0 |
| K | -6.10 | 0 | 62.12 | -4.71 | -0.41 | 0.05 | -0.08 | -0.90 | -5.69 | 0.70 | -0.57 | 0 |
| N | -6.10 | 0 | 53.66 | -5.93 | 0.46 | 0.01 | 0.05 | 0.38 | -0.62 | 0.15 | 0.32 | 1 |
| P | -6.10 | 0 | 41.94 | -1.71 | -0.20 | 0.09 | 0.23 | 0.28 | -1.66 | 0.25 | 0.59 | 1 |
| Q | -6.10 | 0 | 62.49 | -7.19 | -1.82 | -0.08 | 0.50 | 2.84 | 2.47 | 0.70 | -0.70 | 0 |
| R | -6.10 | 0 | 76.97 | -10.89 | -0.23 | -0.09 | 0.37 | 0.56 | 4.27 | 0.10 | -0.10 | 0 |
| T | -6.10 | 0 | 48.03 | -3.18 | 0.56 | -0.02 | -0.02 | -1.17 | -0.26 | 0.77 | -0.65 | 0 |
| V | -6.10 | 0 | 47.87 | -2.90 | -0.50 | 0.09 | 0.05 | -0.62 | -6.12 | 0.70 | -0.52 | 0 |
| Y | -6.10 | 0 | 83.54 | -6.26 | 0.61 | 0.03 | 0.05 | 0.86 | -2.88 | 0.19 | 0.35 | 1 |
| Pearson correlation coefficient to W_ji_ | | | 0.40 | -0.29 | -0.09 |  | | | | | 0.12 |  |

| Position | 102CDR3H | | | | | | | | | | | |
| --- | --- | --- | --- | --- | --- | --- | --- | --- | --- | --- | --- | --- |
| Amino Acid | W_ji_ | δW_ji_ | X_ji_ | Y_ji_ | Z_ji_ | x | y | z | a | t | pW_ji_-t | δpW_ji_ |
| F | 7.51 | 1 | 70.74 | -9.22 | -0.02 | 0.09 | 0.22 | 1.15 | -3.59 | 0.47 | 0.17 | 1 |
| Y | 6.82 | 1 | 70.41 | -16.54 | 1.51 | 0.03 | 0.23 | 0.76 | -2.27 | 0.19 | -0.14 | 0 |
| M | 0.50 | 1 | 48.62 | -7.35 | -1.81 | 0.09 | 0.20 | 0.15 | -3.32 | 0.50 | -0.19 | 0 |
| A | -0.27 | 0 | 27.50 | -6.50 | -0.01 | -0.02 | 0.33 | -0.49 | -1.42 | 0.06 | -0.04 | 0 |
| H | -1.22 | 0 | 60.18 | -13.82 | -1.81 | -0.02 | -0.03 | 0.27 | 0.63 | 0.18 | 0.20 | 1 |
| P | -2.76 | 0 | 41.93 | -8.28 | -1.81 | 0.07 | 0.11 | 0.07 | -1.61 | 0.25 | 0.29 | 1 |
| T | -2.76 | 0 | 42.09 | -11.56 | 0.59 | -0.03 | -0.07 | -1.19 | -0.16 | 0.77 | -0.55 | 0 |
| S | -3.50 | 0 | 34.34 | -11.46 | -0.42 | -0.08 | 0.20 | 3.22 | -0.64 | 0.32 | -0.32 | 0 |
| C | -6.10 | 0 | 35.04 | -8.55 | -1.81 | 0.00 | 0.00 | 0.00 | -102.6 | 0.50 | -0.50 | 0 |
| D | -6.10 | 0 | 47.79 | -10.81 | 0.63 | 0.05 | -0.11 | -0.82 | -2.58 | 0.25 | 0.32 | 1 |
| E | -6.10 | 0 | 55.44 | -12.02 | -0.42 | 0.07 | 0.07 | -0.61 | -4.07 | 0.23 | 0.03 | 1 |
| G | -6.10 | 0 | 20.35 | -4.24 | 0.39 | -0.10 | -0.44 | 0.33 | 0.79 | 0.27 | 0.43 | 1 |
| I | -6.10 | 0 | 48.56 | -6.50 | -1.81 | -0.01 | -0.05 | 0.69 | 0.25 | 0.60 | -0.37 | 0 |
| K | -6.10 | 0 | 47.36 | -10.35 | -0.38 | 0.04 | -0.13 | -0.92 | -5.31 | 0.70 | -0.58 | 0 |
| L | -6.10 | 0 | 48.47 | -8.57 | -0.11 | 0.00 | -0.16 | 1.15 | -0.19 | 0.21 | 0.50 | 1 |
| N | -6.10 | 0 | 48.56 | -11.08 | -0.10 | 0.01 | 0.02 | 0.34 | -0.49 | 0.15 | 0.22 | 1 |
| Q | -6.10 | 0 | 54.71 | -13.79 | -1.81 | -0.08 | 0.50 | 2.84 | 2.47 | 0.70 | -0.70 | 0 |
| R | -6.10 | 0 | 68.08 | -11.15 | -0.20 | -0.09 | 0.37 | 0.56 | 4.26 | 0.10 | -0.10 | 0 |
| V | -6.10 | 0 | 42.21 | -5.67 | -0.47 | 0.08 | 0.03 | -0.77 | -6.15 | 0.70 | -0.62 | 0 |
| W | -6.10 | 0 | 83.08 | -10.24 | 0.87 | 0.03 | 0.09 | 0.41 | -2.88 | 0.70 | -0.45 | 0 |
| Pearson correlation coefficient to W_ji_ | | | 0.30 | -0.26 | 0.21 |  | | | | | 0.15 |  |

| Position | 103CDR3H | | | | | | | | | | | |
| --- | --- | --- | --- | --- | --- | --- | --- | --- | --- | --- | --- | --- |
| Amino Acid | W_ji_ | δW_ji_ | X_ji_ | Y_ji_ | Z_ji_ | x | y | z | a | t | pW_ji_-t | δpW_ji_ |
| L | 5.83 | 1 | 17.19 | 0.91 | -0.14 | 0.00 | -0.08 | 0.66 | -0.87 | 0.21 | 0.04 | 1 |
| A | 0.46 | 1 | 0.00 | 1.26 | -0.58 | 0.00 | 0.31 | -0.76 | -2.13 | 0.06 | 0.16 | 1 |
| R | -0.55 | 0 | 46.41 | -1.37 | 0.36 | -0.09 | 0.35 | 0.84 | 4.85 | 0.10 | 0.47 | 1 |
| I | -1.22 | 0 | 13.08 | 0.41 | -0.31 | -0.02 | -0.06 | 0.82 | 0.76 | 0.60 | -0.05 | 0 |
| W | -1.22 | 0 | 58.94 | -3.15 | -1.82 | 0.03 | 0.11 | 0.35 | -2.72 | 0.70 | -0.60 | 0 |
| T | -2.76 | 0 | 6.64 | 0.73 | 0.20 | -0.03 | -0.03 | -1.17 | 0.01 | 0.77 | -0.39 | 0 |
| C | -6.10 | 0 | 5.70 | 0.44 | -1.82 | 0.00 | 0.00 | 0.00 | -102.6 | 0.50 | -0.50 | 0 |
| D | -6.10 | 0 | 16.78 | 1.00 | 0.24 | 0.05 | -0.04 | -0.83 | -2.58 | 0.25 | -0.13 | 0 |
| E | -6.10 | 0 | 24.19 | 0.82 | 0.47 | 0.06 | 0.10 | -0.54 | -3.90 | 0.23 | -0.16 | 0 |
| F | -6.10 | 0 | 45.95 | 1.10 | -1.82 | 0.09 | 0.20 | 1.23 | -3.57 | 0.47 | -0.28 | 0 |
| G | -6.10 | 0 | 0.00 | 1.76 | 0.01 | -0.11 | -0.28 | 0.42 | 0.93 | 0.27 | 0.34 | 1 |
| H | -6.10 | 0 | 32.92 | -0.66 | -1.82 | -0.02 | 0.01 | 0.24 | 0.69 | 0.18 | 0.25 | 1 |
| K | -6.10 | 0 | 24.17 | -0.35 | -0.41 | 0.04 | -0.10 | -0.93 | -5.40 | 0.70 | -0.68 | 0 |
| M | -6.10 | 0 | 25.31 | 0.71 | -1.82 | 0.09 | 0.17 | 0.34 | -2.79 | 0.50 | -0.25 | 0 |
| N | -6.10 | 0 | 18.48 | 1.17 | -0.13 | 0.00 | 0.07 | 0.33 | -0.37 | 0.15 | 0.29 | 1 |
| P | -6.10 | 0 | 12.40 | 1.19 | 0.76 | 0.07 | 0.20 | 0.28 | -1.22 | 0.25 | 0.26 | 1 |
| Q | -6.10 | 0 | 32.89 | -0.86 | -0.03 | -0.10 | 0.32 | 3.46 | 3.72 | 0.70 | -0.16 | 0 |
| S | -6.10 | 0 | 5.67 | 1.09 | -0.45 | -0.07 | 0.19 | 3.04 | -0.54 | 0.32 | -0.21 | 0 |
| V | -6.10 | 0 | 6.62 | 1.25 | -0.50 | 0.08 | 0.03 | -0.90 | -6.40 | 0.70 | -0.70 | 0 |
| Y | -6.10 | 0 | 45.91 | 0.99 | 1.26 | 0.02 | 0.07 | 0.82 | -2.03 | 0.19 | 0.31 | 1 |
| Pearson correlation coefficient to W_ji_ | | | 0.02 | -0.21 | 0.04 |  | | | | | 0.15 |  |

| Position | 104CDR3H | | | | | | | | | | | |
| --- | --- | --- | --- | --- | --- | --- | --- | --- | --- | --- | --- | --- |
| Amino Acid | W_ji_ | δW_ji_ | X_ji_ | Y_ji_ | Z_ji_ | x | y | z | a | t | pW_ji_-t | δpW_ji_ |
| N | 4.28 | 1 | 11.51 | 2.39 | -0.16 | 0.01 | 0.04 | 0.44 | -0.79 | 0.15 | 0.19 | 1 |
| G | 1.95 | 1 | 0.00 | 0.88 | -0.02 | -0.09 | -0.32 | 0.31 | 0.52 | 0.27 | 0.29 | 1 |
| Q | 1.95 | 1 | 28.16 | -0.47 | -0.06 | 0.00 | 0.92 | 1.75 | -1.08 | 0.70 | -0.55 | 0 |
| F | 1.58 | 1 | 42.59 | 0.15 | 0.52 | 0.09 | 0.18 | 1.16 | -3.89 | 0.47 | 0.19 | 1 |
| H | 1.58 | 1 | 28.07 | 2.27 | 0.41 | -0.01 | -0.01 | 0.23 | 0.28 | 0.18 | 0.33 | 1 |
| R | 1.55 | 1 | 42.42 | -2.32 | -0.26 | -0.07 | 0.55 | 0.54 | 3.30 | 0.10 | 0.16 | 1 |
| S | 0.88 | 1 | 0.00 | 2.59 | 0.08 | -0.07 | 0.18 | 3.39 | -1.08 | 0.32 | 0.10 | 1 |
| Y | 0.59 | 1 | 48.53 | -1.66 | 1.62 | 0.02 | 0.04 | 0.76 | -2.43 | 0.19 | 0.27 | 1 |
| D | 0.50 | 1 | 11.85 | 2.45 | -0.37 | 0.07 | -0.08 | -1.08 | -3.82 | 0.25 | -0.19 | 0 |
| C | -1.22 | 0 | 7.25 | 0.84 | -1.84 | 0.00 | 0.00 | 0.00 | -102.6 | 0.50 | -0.50 | 0 |
| K | -1.22 | 0 | 25.81 | 2.53 | -0.44 | 0.04 | -0.09 | -0.93 | -5.43 | 0.70 | -0.69 | 0 |
| P | -1.25 | 0 | 12.62 | 1.69 | 0.73 | 0.07 | 0.21 | 0.30 | -1.22 | 0.25 | 0.30 | 1 |
| L | -3.50 | 0 | 19.86 | 1.32 | -0.69 | -0.01 | -0.04 | 0.82 | -0.28 | 0.21 | 0.05 | 1 |
| A | -6.10 | 0 | 0.00 | 2.07 | -0.61 | -0.02 | 0.39 | -0.37 | -1.12 | 0.06 | 0.42 | 1 |
| E | -6.10 | 0 | 29.31 | 0.21 | 0.08 | 0.06 | 0.10 | -0.52 | -3.86 | 0.23 | -0.11 | 0 |
| I | -6.10 | 0 | 13.35 | 1.21 | -0.34 | -0.02 | -0.05 | 0.80 | 0.68 | 0.60 | -0.07 | 0 |
| M | -6.10 | 0 | 20.68 | -0.03 | -1.84 | 0.09 | 0.16 | 0.34 | -2.80 | 0.50 | -0.34 | 0 |
| T | -6.10 | 0 | 5.88 | 2.66 | -0.40 | -0.04 | -0.01 | -1.25 | 0.08 | 0.77 | -0.19 | 0 |
| V | -6.10 | 0 | 6.48 | 2.17 | -0.53 | 0.08 | 0.03 | -0.90 | -6.40 | 0.70 | -0.70 | 0 |
| W | -6.10 | 0 | 57.16 | -5.04 | -1.84 | 0.03 | 0.11 | 0.36 | -2.73 | 0.70 | -0.62 | 0 |
| Pearson correlation coefficient to W_ji_ | | | 0.09 | 0.08 | 0.48 |  | | | | | 0.37 |  |

| Position | 105CDR3H | | | | | | | | | | | |
| --- | --- | --- | --- | --- | --- | --- | --- | --- | --- | --- | --- | --- |
| Amino Acid | W_ji_ | δW_ji_ | X_ji_ | Y_ji_ | Z_ji_ | x | y | z | a | t | pW_ji_-t | δpW_ji_ |
| G | 5.81 | 1 | 0.00 | 0.04 | 1.06 | -0.09 | -0.31 | 0.26 | 0.54 | 0.27 | 0.42 | 1 |
| N | 1.58 | 1 | 6.54 | 0.02 | 0.53 | 0.02 | 0.07 | 0.33 | -0.88 | 0.15 | 0.20 | 1 |
| S | 0.88 | 1 | 0.00 | 0.08 | 0.18 | -0.06 | 0.21 | 3.42 | -1.14 | 0.32 | 0.05 | 1 |
| Y | 0.59 | 1 | 39.26 | -1.62 | 1.87 | 0.03 | 0.05 | 0.77 | -2.71 | 0.19 | 0.24 | 1 |
| H | 0.50 | 1 | 25.77 | 0.00 | -1.79 | -0.01 | 0.01 | 0.38 | 0.19 | 0.18 | 0.16 | 1 |
| A | -0.27 | 0 | 0.00 | 0.07 | -1.79 | -0.02 | 0.30 | -0.61 | -1.35 | 0.06 | 0.38 | 1 |
| Q | -0.27 | 0 | 17.09 | 1.28 | -1.79 | -0.08 | 0.50 | 2.63 | 2.49 | 0.70 | -0.65 | 0 |
| C | -1.22 | 0 | 0.00 | 0.01 | -1.79 | 0.00 | 0.00 | 0.00 | -102.6 | 0.50 | -0.50 | 0 |
| K | -1.22 | 0 | 28.21 | 0.04 | -1.79 | 0.04 | -0.09 | -0.94 | -5.27 | 0.70 | -0.63 | 0 |
| M | -1.22 | 0 | 13.56 | 0.02 | -1.79 | 0.09 | 0.16 | 0.34 | -2.83 | 0.50 | -0.41 | 0 |
| P | -2.76 | 0 | 0.00 | 0.13 | -1.79 | 0.07 | 0.18 | 0.10 | -1.55 | 0.25 | -0.10 | 0 |
| R | -3.50 | 0 | 43.02 | -2.34 | -0.17 | -0.10 | 0.30 | 0.62 | 4.93 | 0.10 | 0.36 | 1 |
| D | -6.10 | 0 | 0.00 | 0.02 | 0.30 | 0.05 | -0.05 | -0.83 | -2.59 | 0.25 | -0.19 | 0 |
| E | -6.10 | 0 | 5.33 | 0.02 | 0.18 | 0.07 | 0.10 | -0.62 | -4.08 | 0.23 | -0.21 | 0 |
| F | -6.10 | 0 | 29.32 | 0.18 | -1.79 | 0.09 | 0.19 | 1.26 | -3.65 | 0.47 | -0.43 | 0 |
| I | -6.10 | 0 | 10.83 | 0.01 | -1.79 | -0.02 | -0.05 | 0.64 | 0.44 | 0.60 | -0.31 | 0 |
| L | -6.10 | 0 | 12.41 | 0.92 | -0.60 | -0.01 | -0.06 | 0.80 | -0.13 | 0.21 | 0.10 | 1 |
| T | -6.10 | 0 | 4.08 | 0.10 | -0.31 | -0.04 | -0.06 | -1.30 | 0.23 | 0.77 | -0.16 | 0 |
| V | -6.10 | 0 | 4.07 | 0.53 | -1.79 | 0.08 | 0.03 | -0.90 | -6.35 | 0.70 | -0.69 | 0 |
| W | -6.10 | 0 | 41.81 | -0.01 | 0.90 | 0.02 | 0.12 | 0.39 | -2.43 | 0.70 | -0.45 | 0 |
| Pearson correlation coefficient to W_ji_ | | | -0.11 | -0.11 | 0.19 |  | | | | | 0.47 |  |
