# Supplementary material for: Rationalization and Design of the Complementarity Determining Region Sequences in an Antibody-Antigen Recognition Interface
Source: PLoS One. 2012 Mar 22;7(3):e33340. doi: 10.1371/journal.pone.0033340 (PMC3310866; doi:10.1371/journal.pone.0033340)
Supplement: Table S5 — Ranking antibody CDR single site amino acid binding to VEGF with public domain scoring functions. Table S5b, the top-ranked amino acid types and rotamers with various scoring systems. (DOC) [file pone.0033340.s006.doc]

**Table S5a**. Ranking antibody CDR single site amino acid binding to VEGF with public domain scoring functions. Column ‘Site’ shows the antibody residue location to be tested as numbered in G6-Fab structure (2FJG in PDB code); the parenthesis indicates the amino acid type and the penultimate rotamer conformation to be ranked among the 154 amino acid types and conformers by the scoring functions. The residue conformation shown in this column superimposes with the crystallographic structure of the amino acid with the lowest RMSD. These sidechain structures are the near-crystallographic rotameric model structures. The model ‘drugs’ to be ranked are composed of amino acid sidechain atoms plus backbone atoms from the indicated residue and one neighboring residue at the N- and C-terminus of the residue. The amino acid sidechains were modeled with 154 penultimate rotamer conformations for the 20 natural amino acid types ; the mainchain atoms remained fixed as in the template structure 2FJG. All other antibody atoms were not included in the computation of binding affinity. The modeled single site amino acid sidechain conformers that clashed with VEGF or with the backbone atoms after optimization were eliminated from further consideration. The ‘Available conformers’ column shows the total number of non-clashed amino acid model conformers including the near-crystallographic conformation as shown in the ‘Site’ column,. All the available conformers were ranked with scoring functions (A1~H1, for which the names are listed in Table 5b) for descending affinity to VEGF. The A1~H1 columns indicate the rank of the near-crystallographic structure of the amino acid shown in the ‘Site’ column among the available conformers. The parenthesis indicates the top-ranked amino acid type and the penultimate rotamer identity. In comparison, the available conformers were ranked with descending affinity to VEGF with the scores based on *X_ji_* shown in Equation (4), which were calculated with the 30 probability density maps on the VEGF surface in the absence of the antibody structure. The ‘*X_ji_*’ column shows the rank of the near-crystallographic structure of the amino acid among the available conformers. As shown in the Table, the ranks vary among scoring functions A1~H1. But nevertheless, the *X_ji_* ranks are comparable with the ranges of the ranks from the scoring functions, indicating that the *X_ji_* term has included the essence of atomistic contact energetics formulated in the scoring functions based on first principles.

| Site | Available conformers | *X_ji_* | A1 | A2 | A3 | A4 | A5 | A6 | A7 | A8 | A9 | A10 | B1 | B2 | B3 | B4 | C1 | D1 | D2 | D3 | D4 | D5 | E1 | F1 | G1 | H1 |
| --- | --- | --- | --- | --- | --- | --- | --- | --- | --- | --- | --- | --- | --- | --- | --- | --- | --- | --- | --- | --- | --- | --- | --- | --- | --- | --- |
| H-31(D5) | 150 | 77  (W4) | 54 (R34) | 43 (R07) | 32 (W6) | 43 (W2) | 108 (Q5) | 72 (W7) | 90 (W7) | 94 (K17) | 100 (K17) | 96 (W2) | 18 (W7) | 83 (W4) | 25 (W4) | 43 (W4) | 97 (F4) | 133 (W2) | 83 (R34) | 15 (E3) | 115 (W4) | 29 (P2) | 102 (W7) | 88 (R16) | 4 (W2) | 21 (R26) |
| H-32(Y4) | 151 | 4  (W6) | 23 (R32) | 13 (R24) | 12 (R30) | 11 (R32) | 11 (R18) | 113 (K16) | 138 (K16) | 33 (P2) | 15 (P1) | 14 (P1) | 3 (W6) | 27 (P1) | 1 (Y4) | 7 (R24) | 26 (M9) | 132 (K26) | 131 (K16) | 6 (W3) | 99 (P1) | 5 (W6) | 22 (L5) | 8 (K27) | 5 (W5) | 2 (R30) |
| H-33(W6) | 152 | 4  (W5) | 15 (R32) | 7 (R8) | 1 (W6) | 1 (W6) | 6 (W5) | 1 (W6) | 1 (W6) | 2 (W7) | 6 (W5) | 1 (W6) | 1 (W6) | 1 (W6) | 1 (W6) | 1 (W6) | 6 (W2) | 3 (W5) | 1 (W6) | 3 (W5) | 1 (W6) | 2 (P1) | 3 (W5) | 1 (W6) | 2 (H6) | 1 (W6) |
| H-54(A1) | 154 | 154  (W6) | 97 (R34) | 59 (R34) | 102 (R34) | 134 (R34) | 57 (W6) | 151 (W6) | 151 (W5) | 32 (R22) | 32 (R22) | 37 (W6) | 27 (H6) | 17 (P2) | 86 (Y2) | 50 (W3) | 83 (F2) | 123 (R33) | 150 (Q8) | 144 (K21) | 48 (W6) | 4 (G1) | 76 (W6) | 41 (N3) | 87 (W4) | 36 (K18) |
| H-56(G1) | 126 | 126  (W4) | 1 (G1) | 1 (G1) | 15 (P1) | 25 (P1) | 124 (R13) | 4 (P2) | 1 (G1) | 56 (K12) | 61 (K12) | 67 (W7) | 1 (G1) | 5 (P2) | 1 (G1) | 1 (G1) | 25 (P2) | 124 (K8) | 1 (G1) | 126 (W7) | 78 (W7) | 1 (G1) | 114 (W7) | 1 (G1) | 4 (P2) | 1 (G1) |
| H-101(F4) | 152 | 9  (W6) | 68 (R14) | 36 (K18) | 5 (W6) | 4 (W6) | 30 (R4) | 4 (W7) | 7 (W6) | 26 (R11) | 27 (R11) | 16 (W6) | 2 (D5) | 2 (W6) | 3 (W6) | 3 (W6) | 6 (M12) | 26 (W6) | 13 (W7) | 34 (M2) | 7 (W6) | 3 (P1) | 7 (W6) | 42 (P1) | 4 (P1) | 5 (P1) |
| H-102(F1) | 113 | 7  (W2) | 47 (N4) | 32 (C2) | 4 (W2) | 4 (W2) | 21 (E6) | 70 (G1) | 66 (G1) | 33 (W4) | 21 (W4) | 23 (W4) | 4 (D2) | 1 (F1) | 14 (R7) | 1 (F1) | 30 (T2) | 16 (W2) | 72 (G1) | 33 (E3) | 11 (Y3) | 3 (W2) | 9 (W2) | 3 (E2) | 3 (W1) | 29 (H1) |
| H-103(L5) | 147 | 68  (R31) | 54 (R30) | 55 (R30) | 43 (R30) | 47 (R31) | 79 (W7) | 82 (R30) | 82 (K25) | 25 (P1) | 34 (P2) | 32 (P2) | 19 (R31) | 8 (W2) | 53 (R30) | 22 (K25) | 29 (F3) | 29 (W7) | 118 (R30) | 40 (E8) | 88 (F1) | 22 (P2) | 5 (P2) | 44 (W5) | 23 (W7) | 62 (K25) |

**Table S5b**. The top-ranked amino acid types and rotamers with various scoring systems. Following Table 5a, the top three amino acid types ranked by *X_ji_* are shown in column 3 to 5. The number after the amino acid type is the penultimate rotamer identity . For comparison, the top ranked amino acid types based on the 24 scoring functions are shown in the following columns. Column 6 shows the most consensual amino acid type; the fraction number before the amino acid type is the number of scoring functions out of the total 24 reaching the most consensual amino acid type. Column 7 shows the second most consensual amino acid type, and so on. As shown in the Table, the consensus for the top-ranked amino acid types varies among the scoring functions, except for the site 33W, where 20 of the 24 scoring functions reach the majority consensus. But nevertheless, the most consensual amino acid type from the scoring functions at each of amino acid positions is in good agreement with the top ranked amino acid type by *X_ji_*, suggesting that the *X_ji_* ranks have included the essence of atomistic contact energetics that has been formulated in the scoring functions. Based on the diverse results shown in Supplementary Table 5a and 5b, it is difficult to envisage that the scoring functions (A1~H1) are more effective in ranking amino acid preferences than the ranking system based on *X_ji_*.

| CDR group | site | *X_ji_* | | | Statistics of the top-ranked amino acid type and rotamer conformer from the 24 scoring functions (A1~H1) | | | | | | | | | | |
| --- | --- | --- | --- | --- | --- | --- | --- | --- | --- | --- | --- | --- | --- | --- | --- |
|  |  | 1st | 2nd | 3rd |  |  |  |  |  |  |  |  |  |  |  |
| H1 | 31D | W4 | Y3 | F4 | 13/24W | 5/24R | 2/24K | 1/24E | 1/24F | 1/24P | 1/24Q |  |  |  |  |
| H1 | 32Y | W6 | Y4 | R10 | 7/24R | 5/24K | 5/24P | 4/24W | 1/24L | 1/24M | 1/24Y |  |  |  |  |
| H1 | 33W | W5 | R32 | Y4 | 20/24W | 2/24R | 1/24H | 1/24P |  |  |  |  |  |  |  |
| H2 | 54A | W6 | Y3 | R32 | 8/24W | 7/24R | 2/24K | 1/24F | 1/24G | 1/24H | 1/24P | 1/24Q | 1/24Y | 1/24N |  |
| H2 | 56G | W4 | F2 | Y2 | 10/24G | 6/24P | 4/24W | 3/24K | 1/24R |  |  |  |  |  |  |
| H3 | 101F | W6 | Y3 | F3 | 12/24W | 4/24P | 4/24R | 2/24M | 1/24D | 1/24K |  |  |  |  |  |
| H3 | 102F | W2 | Y3 | F4 | 9/24W | 3/24E | 3/24G | 2/24F | 1/24C | 1/24D | 1/24H | 1/24N | 1/24R | 1/24T | 1/24Y |
| H3 | 103L | R31 | W1 | F4 | 8/24R | 5/24P | 5/24W | 3/24K | 2/24F | 1/24E |  |  |  |  |  |

List of scoring functions used.

| **A1**  LigScore1_Dreiding |  | **A7**  -PMF04 |  | **B3**  ASP |  | **D4**  CHEMSCORE |
| --- | --- | --- | --- | --- | --- | --- |
| **A2**  LigScore2_Dreiding |  | **A8**  Ludi_1 |  | **B4**  ChemPLP |  | **D5**  Surflex_Score |
| **A3**  -PLP1 |  | **A9**  Ludi_2 |  | **C1**  SCORE |  | **E1**  Xscore |
| **A4**  -PLP2 |  | **A10**  Ludi_3 |  | **D1**  G_SCORE |  | **F1**  Glide_SCORE |
| **A5**  Jain |  | **B1**  Goldscore |  | **D2**  PMF_SCORE |  | **G1**  AutoDockScore |
| **A6**  -PMF |  | **B2**  Chemscore |  | **D3**  D_SCORE |  | **H1**  GridEnergy |

1. Fuh G, Wu P, Liang W-C, Ultsch M, Lee CV, et al. (2006) Structure-Function Studies of Two Synthetic Anti-vascular Endothelial Growth Factor Fabs and Comparison with the Avastin™ Fab. Journal of Biological Chemistry 281: 6625-6631.

2. Lovell SC, Word JM, Richardson JS, Richardson DC (2000) The penultimate rotamer library. Proteins: Structure, Function, and Bioinformatics 40: 389-408.

3. Krammer A, Kirchhoff PD, Jiang X, Venkatachalam CM, Waldman M (2005) LigScore: a novel scoring function for predicting binding affinities. J Mol Graph Model 23: 395-407.

4. Muegge I (2006) PMF scoring revisited. J Med Chem 49: 5895-5902.

5. Korb O, Stutzle T, Exner TE (2009) Empirical scoring functions for advanced protein-ligand docking with PLANTS. J Chem Inf Model 49: 84-96.

6. Eldridge MD, Murray CW, Auton TR, Paolini GV, Mee RP (1997) Empirical scoring functions: I. The development of a fast empirical scoring function to estimate the binding affinity of ligands in receptor complexes. J Comput Aided Mol Des 11: 425-445.

7. Bohm HJ (1994) The development of a simple empirical scoring function to estimate the binding constant for a protein-ligand complex of known three-dimensional structure. J Comput Aided Mol Des 8: 243-256.

8. Jain AN (2003) Surflex: fully automatic flexible molecular docking using a molecular similarity-based search engine. J Med Chem 46: 499-511.

9. Gehlhaar DK, Verkhivker GM, Rejto PA, Sherman CJ, Fogel DB, et al. (1995) Molecular recognition of the inhibitor AG-1343 by HIV-1 protease: conformationally flexible docking by evolutionary programming. Chem Biol 2: 317-324.

10. Renxiao Wang LL, Luhua Lai, and Youqi Tang (1998) SCORE: A New Empirical Method for Estimating the Binding Affinity of a Protein-Ligand Complex. J Mol Model 4: 379-394.

11. Wang R, Lai L, Wang S (2002) Further development and validation of empirical scoring functions for structure-based binding affinity prediction. Journal of Computer-Aided Molecular Design 16: 11-26.

12. Clark RD, Strizhev A, Leonard JM, Blake JF, Matthew JB (2002) Consensus scoring for ligand/protein interactions. J Mol Graph Model 20: 281-295.

13. Friesner RA, Banks JL, Murphy RB, Halgren TA, Klicic JJ, et al. (2004) Glide: a new approach for rapid, accurate docking and scoring. 1. Method and assessment of docking accuracy. J Med Chem 47: 1739-1749.

14. Jain AN (1996) Scoring noncovalent protein-ligand interactions: a continuous differentiable function tuned to compute binding affinities. J Comput Aided Mol Des 10: 427-440.

15. Jones G, Willett P, Glen RC (1995) Molecular recognition of receptor sites using a genetic algorithm with a description of desolvation. J Mol Biol 245: 43-53.

16. Morris GM, Goodsell DS, Halliday RS, Huey R, Hart WE, et al. (1998) Automated docking using a Lamarckian genetic algorithm and an empirical binding free energy function. Journal of Computational Chemistry 19: 1639-1662.

17. Muegge I, Martin YC (1999) A general and fast scoring function for protein-ligand interactions: a simplified potential approach. J Med Chem 42: 791-804.

18. Ewing TJA, Makino S, Skillman AG, Kuntz ID (2001) DOCK 4.0: Search strategies for automated molecular docking of flexible molecule databases. Journal of Computer-Aided Molecular Design 15: 411-428.
