# Supplementary material for: Rationalization and Design of the Complementarity Determining Region Sequences in an Antibody-Antigen Recognition Interface
Source: PLoS One. 2012 Mar 22;7(3):e33340. doi: 10.1371/journal.pone.0033340 (PMC3310866; doi:10.1371/journal.pone.0033340)
Supplement: Table S6 — Amino acid conformation classifications. (DOC) [file pone.0033340.s007.doc]

**Table S6.** Amino acid conformation classifications. Geometric parameter distributions and clustering criteria are shown for each amino acid type and conformational clusters with side-chain chi1, chi2, chi3 and chi4 and main-chain phi and psi dihedral angles for the centroid conformations.

| ID | Conf. cluster | PHI | PSI | CHI1 | CHI2 | CHI3 | CHI4 | Conformer Percentage(%) | RMSD  Mean(Å) | RMSD  Variance(Å) |
| --- | --- | --- | --- | --- | --- | --- | --- | --- | --- | --- |
| 1 | A1 | -82.142 | -9.6013 |  |  |  |  | 10.01 | 0.179 | 0.018 |
| 2 | A2 | -116.43 | -154.16 |  |  |  |  | 0.78 | 0.267 | 0.015 |
| 3 | A3 | -67.793 | 142.8 |  |  |  |  | 13.36 | 0.145 | 0.008 |
| 4 | A4 | -61.851 | -41.202 |  |  |  |  | 53.29 | 0.108 | 0.014 |
| 5 | A5 | -107.55 | 127.66 |  |  |  |  | 10.28 | 0.203 | 0.017 |
| 6 | A6 | -148.18 | 151.86 |  |  |  |  | 12.28 | 0.132 | 0.004 |
| Total number of conformer : 154325, Partition Index: 0.0014, Separation Index: 1.65E-08 | | | | | | | | | | |
| 7 | C1 | -78.619 | -25.132 | 76.137 |  |  |  | 9.68 | 0.482 | 0.088 |
| 8 | C2 | -109.11 | 117.19 | -168.43 |  |  |  | 11.66 | 0.259 | 0.016 |
| 9 | C3 | -102.49 | 138.96 | -62.872 |  |  |  | 24.44 | 0.236 | 0.015 |
| 10 | C4 | -68.589 | -34.663 | -72.594 |  |  |  | 36.22 | 0.304 | 0.072 |
| 11 | C5 | -141.95 | 159.21 | 62.667 |  |  |  | 9.62 | 0.233 | 0.016 |
| 12 | C6 | -103.93 | 120.53 | 170.01 |  |  |  | 8.38 | 0.234 | 0.010 |
| Total number of conformer : 29291, Partition Index: 0.0019, Separation Index: 8.94E-08 | | | | | | | | | | |
| 13 | D1 | -90.263 | -5.963 | 58.743 | -12.115 |  |  | 12.86 | 0.672 | 0.147 |
| 14 | D2 | -71.108 | -20.619 | -79.542 | 118.4 |  |  | 10.36 | 0.848 | 0.146 |
| 15 | D3 | -98.758 | 106.39 | -165.14 | -12.913 |  |  | 21.58 | 0.471 | 0.072 |
| 16 | D4 | -67.646 | -34.448 | -71.413 | -25.106 |  |  | 33.07 | 0.384 | 0.098 |
| 17 | D5 | -85.179 | 132.89 | -73.55 | -23.595 |  |  | 11.46 | 0.421 | 0.068 |
| 18 | D6 | -98.215 | 106.8 | 156.72 | 11.861 |  |  | 10.67 | 0.652 | 0.100 |
| Total number of conformer : 85476, Partition Index: 0.0032, Separation Index: 4.66E-08 | | | | | | | | | | |
| 19 | E1 | -69.204 | -18.681 | -109.19 | 81.349 | 27.178 |  | 10.47 | 0.900 | 0.077 |
| 20 | E2 | -103.85 | 125.63 | -74.788 | 149.08 | 3.8893 |  | 13.45 | 0.805 | 0.098 |
| 21 | E3 | -71.368 | -21.747 | -64.094 | -70.332 | -31.718 |  | 12.67 | 0.635 | 0.208 |
| 22 | E4 | -103.96 | 127.14 | -55.92 | -140.21 | -8.1659 |  | 17.93 | 0.807 | 0.083 |
| 23 | E5 | -70.698 | -21.246 | -67.657 | -156.37 | -2.6547 |  | 18.27 | 0.732 | 0.103 |
| 24 | E6 | -72.395 | -8.5072 | 153.26 | 103.48 | 15.235 |  | 10.23 | 0.885 | 0.096 |
| 25 | E7 | -68.109 | -29.53 | -75.578 | 158.95 | -8.4272 |  | 16.98 | 0.547 | 0.105 |
| Total number of conformer : 87635, Partition Index: 0.0031, Separation Index: 4.82E-08 | | | | | | | | | | |
| 26 | F1 | -65.06 | -39.612 | -147.63 | 76.556 |  |  | 19.55 | 0.808 | 0.168 |
| 27 | F2 | -109.68 | 131.88 | -63.333 | 86.014 |  |  | 16.76 | 0.331 | 0.037 |
| 28 | F3 | -62.803 | -43.236 | 167.33 | 69.859 |  |  | 12.48 | 0.460 | 0.230 |
| 29 | F4 | -136.26 | 147.98 | 87.349 | 76.702 |  |  | 11.36 | 0.753 | 0.148 |
| 30 | F5 | -77.92 | -22.377 | -69.948 | -59.487 |  |  | 16.26 | 0.552 | 0.188 |
| 31 | F6 | -108.88 | 134.04 | -60.765 | -77.5 |  |  | 17.50 | 0.596 | 0.288 |
| 32 | F7 | -100.3 | 111.99 | -153.61 | 69.477 |  |  | 6.09 | 0.386 | 0.021 |
| Total number of conformer : 84976, Partition Index: 0.0021, Separation Index: 3.37E-08 | | | | | | | | | | |
| 33 | G1 | 107.04 | -156.68 |  |  |  |  | 12.79 | 0.239 | 0.008 |
| 34 | G2 | -119.52 | -160.73 |  |  |  |  | 9.23 | 0.209 | 0.007 |
| 35 | G3 | -110.38 | 153.24 |  |  |  |  | 19.19 | 0.234 | 0.010 |
| 36 | G4 | -66.23 | -34.586 |  |  |  |  | 26.27 | 0.152 | 0.012 |
| 37 | G5 | 86.04 | 6.9536 |  |  |  |  | 23.96 | 0.161 | 0.009 |
| 38 | G6 | 116.61 | 158.61 |  |  |  |  | 8.57 | 0.206 | 0.008 |
| Total number of conformer : 125926, Partition Index: 9.69E-04, Separation Index: 1.26E-08 | | | | | | | | | | |
| 39 | H1 | -87.461 | 127.8 | -158.4 | -81.972 |  |  | 7.81 | 0.397 | 0.047 |
| 40 | H2 | -102.06 | 103.43 | -69.454 | 84.629 |  |  | 5.99 | 0.821 | 0.144 |
| 41 | H3 | -107.11 | 112.5 | 155.25 | 60.447 |  |  | 7.34 | 0.829 | 0.115 |
| 42 | H4 | -75.625 | -24.897 | -65.608 | 110.29 |  |  | 13.75 | 0.534 | 0.095 |
| 43 | H5 | -64.036 | -38.092 | -162.63 | 68.871 |  |  | 5.46 | 0.351 | 0.055 |
| 44 | H6 | -67.634 | -32.834 | -73.74 | -71.611 |  |  | 15.24 | 0.665 | 0.183 |
| 45 | H7 | -114.27 | 136.78 | -65.127 | 85.436 |  |  | 8.67 | 0.563 | 0.143 |
| 46 | H8 | -67.795 | -28.218 | 142.26 | -58.257 |  |  | 11.67 | 0.977 | 0.085 |
| 47 | H9 | -99.1 | 6.1504 | -60.747 | -74.739 |  |  | 7.20 | 0.385 | 0.042 |
| 48 | H10 | -113.58 | 136.07 | -56.917 | -82.811 |  |  | 16.86 | 0.549 | 0.158 |
| Total number of conformer : 42465, Partition Index: 0.0028, Separation Index: 9.08E-08 | | | | | | | | | | |
| 49 | I1 | -65.415 | -43.135 | -67.744 | 166.89 |  |  | 32.68 | 0.367 | 0.195 |
| 50 | I2 | -119.12 | 122.99 | -62.772 | 166.46 |  |  | 19.41 | 0.283 | 0.105 |
| 51 | I3 | -67.241 | -41.923 | -62.275 | -71.517 |  |  | 11.01 | 0.384 | 0.106 |
| 52 | I4 | -101.22 | 121.42 | -55.136 | -61.119 |  |  | 9.21 | 0.231 | 0.044 |
| 53 | I5 | -109.86 | 116.21 | -54.356 | -171.17 |  |  | 6.69 | 0.422 | 0.174 |
| 54 | I6 | -120.56 | 145.74 | 64.441 | 164.77 |  |  | 8.78 | 0.482 | 0.156 |
| 55 | I7 | -91.956 | 118.75 | -65.116 | 163.04 |  |  | 12.21 | 0.292 | 0.082 |
| Total number of conformer : 123327, Partition Index: 0.0014, Separation Index: 1.40E-08 | | | | | | | | | | |
| 56 | K1 | -73.566 | -7.1543 | -82.14 | 157.34 | -156.49 | 151.07 | 9.96 | 0.976 | 0.143 |
| 57 | K2 | -79.647 | 14.92 | -82.202 | 145.3 | -137.44 | -107 | 9.94 | 1.054 | 0.097 |
| 58 | K3 | -72.18 | -14.126 | -67.263 | -127.24 | -119.63 | -129.34 | 9.58 | 0.758 | 0.053 |
| 59 | K4 | -83.063 | 34.099 | 87 | 101.4 | 92.003 | 52.033 | 6.35 | 1.073 | 0.025 |
| 60 | K5 | -73.157 | -12.036 | -66.707 | -122.48 | -134.67 | 135.17 | 7.16 | 0.738 | 0.053 |
| 61 | K6 | -71.114 | -15.814 | -80.191 | 151.16 | 133.88 | 138.9 | 7.12 | 0.707 | 0.083 |
| 62 | K7 | -96.055 | 95.285 | -62.724 | 122.82 | 98.091 | 78.115 | 5.17 | 0.881 | 0.031 |
| 63 | K8 | -101.31 | 110.26 | -49.99 | -109.13 | 136.04 | -109.05 | 5.37 | 1.010 | 0.120 |
| 64 | K9 | -97.122 | 103.08 | -54.428 | -90.6 | -123.7 | 106.2 | 5.50 | 0.849 | 0.034 |
| 65 | K10 | -73.265 | -16.224 | -67.496 | -142.71 | 152.6 | -147.65 | 6.65 | 0.779 | 0.103 |
| 66 | K11 | -76.411 | -0.66278 | -66.449 | -138.29 | 141.03 | 128.09 | 11.28 | 0.930 | 0.096 |
| 67 | K12 | -99.686 | 111.97 | -55.974 | -102.33 | -130.79 | -114 | 7.59 | 0.791 | 0.056 |
| 68 | K13 | -74.477 | -4.71 | -80.937 | 144.52 | 130.59 | -132.08 | 8.58 | 0.872 | 0.083 |
| Total number of conformer : 67791, Partition Index: 0.0024, Separation Index: 4.28E-08 | | | | | | | | | | |
| 69 | L1 | -70.291 | -31.002 | -69.761 | 168.94 |  |  | 27.52 | 0.224 | 0.029 |
| 70 | L2 | -101.8 | 137.93 | -68.637 | 160.41 |  |  | 23.36 | 0.592 | 0.178 |
| 71 | L3 | -70.704 | -30.101 | -73.394 | -168.06 |  |  | 12.14 | 0.336 | 0.068 |
| 72 | L4 | -63.104 | -43.409 | 171.38 | 62.568 |  |  | 7.95 | 0.202 | 0.051 |
| 73 | L5 | -96.064 | 134.03 | -68.125 | -164.58 |  |  | 8.29 | 0.376 | 0.076 |
| 74 | L6 | -109.73 | 124.02 | 167.53 | 66.821 |  |  | 7.66 | 0.318 | 0.076 |
| 75 | L7 | -66.259 | -30.949 | -158.26 | 55.694 |  |  | 13.09 | 0.427 | 0.074 |
| Total number of conformer : 195756, Partition Index: 9.70E-04, Separation Index: 5.84E-09 | | | | | | | | | | |
| 76 | M1 | -71.922 | -22.202 | -66 | -160.29 | 73.812 |  | 7.29 | 0.618 | 0.126 |
| 77 | M2 | -106.17 | 110.29 | -76.654 | 157.82 | -88.483 |  | 9.65 | 0.869 | 0.127 |
| 78 | M3 | -108.7 | 132.87 | -52.977 | -153.94 | 70.182 |  | 8.50 | 0.753 | 0.105 |
| 79 | M4 | -68.158 | -32.486 | -75.158 | 164.1 | 71.084 |  | 14.45 | 0.614 | 0.144 |
| 80 | M5 | -70.699 | -24.971 | -66.77 | -56.255 | 100.53 |  | 5.67 | 0.438 | 0.116 |
| 81 | M6 | -69.391 | -28.276 | -68.679 | -56.41 | -70.667 |  | 15.81 | 0.447 | 0.125 |
| 82 | M7 | -103.92 | 124.05 | -77.263 | 156.88 | 70.712 |  | 7.38 | 0.678 | 0.086 |
| 83 | M8 | -98.782 | 131.27 | -58.696 | -78.65 | -72.985 |  | 13.48 | 0.730 | 0.110 |
| 84 | M9 | -69.839 | -27.824 | -68.4 | -162.13 | -79.179 |  | 9.37 | 0.461 | 0.080 |
| 85 | M10 | -103.69 | 94.787 | 144.15 | 125.14 | 46.469 |  | 8.39 | 0.894 | 0.058 |
| Total number of conformer : 42216, Partition Index: 0.0024, Separation Index: 7.66E-08 | | | | | | | | | | |
| 86 | N1 | -83.917 | -5.6463 | -73.371 | -57.038 |  |  | 13.54 | 0.525 | 0.144 |
| 87 | N2 | -95.342 | 6.3002 | 63.754 | -12.46 |  |  | 11.08 | 0.677 | 0.144 |
| 88 | N3 | -97.451 | 132.17 | -65.91 | -50.797 |  |  | 12.36 | 0.397 | 0.054 |
| 89 | N4 | -101.73 | 109.12 | 154.28 | -4.7755 |  |  | 9.69 | 0.713 | 0.062 |
| 90 | N5 | -96.178 | 104.32 | -164.09 | -25.792 |  |  | 16.03 | 0.468 | 0.063 |
| 91 | N6 | -97.238 | 122.17 | -70.893 | 97.232 |  |  | 6.60 | 0.650 | 0.096 |
| 92 | N7 | -63.285 | -34.657 | -73.964 | -22.311 |  |  | 20.50 | 0.412 | 0.114 |
| 93 | N8 | -69.688 | -24.427 | -73.737 | 128.67 |  |  | 10.20 | 0.629 | 0.149 |
| Total number of conformer : 68552, Partition Index: 0.0035, Separation Index: 6.59E-08 | | | | | | | | | | |
| 94 | P1 | -61.149 | 134.91 |  |  |  |  | 30.23 | 0.304 | 0.016 |
| 95 | P2 | -71.04 | 155.62 |  |  |  |  | 31.73 | 0.248 | 0.017 |
| 96 | P3 | -71.574 | -12.952 |  |  |  |  | 13.73 | 0.293 | 0.021 |
| 97 | P4 | -56.097 | -38.952 |  |  |  |  | 24.31 | 0.318 | 0.029 |
| Total number of conformer : 68722, Partition Index: 0.0013, Separation Index: 2.10E-08 | | | | | | | | | | |
| 98 | Q1 | -71.575 | -20.079 | -63.904 | -146.57 | 64.741 |  | 11.77 | 0.828 | 0.115 |
| 99 | Q2 | -71.922 | -21.656 | -71.791 | 159.13 | 46.315 |  | 8.76 | 0.601 | 0.081 |
| 100 | Q3 | -106.98 | 127.94 | -52.716 | -149.09 | 31.989 |  | 11.86 | 0.864 | 0.087 |
| 101 | Q4 | -69.086 | -28.723 | -70.292 | 163.88 | -45.085 |  | 12.29 | 0.601 | 0.127 |
| 102 | Q5 | -65.935 | -30.796 | -157.17 | 65.383 | 45.805 |  | 6.42 | 0.566 | 0.092 |
| 103 | Q6 | -72.618 | -24.029 | -64.127 | -60.339 | -46.113 |  | 9.39 | 0.554 | 0.144 |
| 104 | Q7 | -106.08 | 131.59 | -72.175 | 156.16 | -8.9235 |  | 13.91 | 0.805 | 0.086 |
| 105 | Q8 | -101.83 | 132.77 | -55.142 | -73.412 | -41.454 |  | 7.85 | 0.750 | 0.197 |
| 106 | Q9 | -71.113 | -22.69 | -64.129 | -159.53 | -41.663 |  | 8.85 | 0.624 | 0.102 |
| 107 | Q10 | -73.95 | -0.81599 | 155.61 | 99.656 | 22.42 |  | 8.90 | 0.947 | 0.071 |
| Total number of conformer : 54694, Partition Index: 0.0028, Separation Index: 7.49E-08 | | | | | | | | | | |
| 108 | R1 | -69.778 | -20.085 | -68.775 | -145.72 | -72.154 | -85.998 | 10.28 | 0.932 | 0.165 |
| 109 | R2 | -77.786 | 12.227 | -74.363 | 140.12 | -93.52 | 114.76 | 9.50 | 1.412 | 0.166 |
| 110 | R3 | -82.297 | 21.243 | -58.365 | -123.51 | 88.317 | -103.28 | 10.59 | 1.442 | 0.121 |
| 111 | R4 | -80.285 | 16.264 | -64.978 | 137.48 | 92.566 | 92.867 | 10.21 | 1.088 | 0.069 |
| 112 | R5 | -102.78 | 102.96 | -49.575 | -92.159 | -89.086 | -76.521 | 8.23 | 1.074 | 0.047 |
| 113 | R6 | -85.754 | 42.382 | -52.476 | -117.67 | 79.797 | 81.037 | 10.60 | 1.446 | 0.132 |
| 114 | R7 | -77.826 | 7.2829 | -73.277 | 140.23 | 68.506 | -116.46 | 10.79 | 1.216 | 0.125 |
| 115 | R8 | -89.179 | 57.151 | 34.91 | 94.565 | 40.8 | 39.151 | 7.57 | 1.519 | 0.033 |
| 116 | R9 | -77.218 | 1.4227 | -67.373 | -130.66 | -84.294 | 112.95 | 13.09 | 1.167 | 0.093 |
| 117 | R10 | -89.116 | 58.75 | -46.323 | 115.46 | -61.04 | -66.681 | 9.14 | 1.939 | 0.099 |
| Total number of conformer : 83239, Partition Index: 0.0039, Separation Index: 5.65E-08 | | | | | | | | | | |
| 118 | S1 | -66.297 | -38.237 | -71.084 |  |  |  | 21.85 | 0.324 | 0.087 |
| 119 | S2 | -145.89 | 157.4 | 66.36 |  |  |  | 11.41 | 0.174 | 0.008 |
| 120 | S3 | -72.687 | -26.372 | 69.151 |  |  |  | 25.74 | 0.318 | 0.071 |
| 121 | S4 | -103.47 | 129.9 | 168.87 |  |  |  | 11.23 | 0.240 | 0.009 |
| 122 | S5 | -98.997 | 138.07 | -64.032 |  |  |  | 11.63 | 0.242 | 0.014 |
| 123 | S6 | -77.462 | 156.11 | 67.174 |  |  |  | 11.32 | 0.176 | 0.009 |
| 124 | S7 | -116.05 | 125.46 | -168.07 |  |  |  | 6.81 | 0.286 | 0.017 |
| Total number of conformer : 98527, Partition Index: 0.0019, Separation Index: 2.63E-08 | | | | | | | | | | |
| 125 | T1 | -111.33 | 157.03 | 65.304 |  |  |  | 25.47 | 0.279 | 0.053 |
| 126 | T2 | -110.24 | 130.88 | -70.67 |  |  |  | 27.39 | 0.354 | 0.073 |
| 127 | T3 | -89.554 | -17.303 | 60.142 |  |  |  | 22.19 | 0.312 | 0.067 |
| 128 | T4 | -65.932 | -44.229 | -62.341 |  |  |  | 24.94 | 0.222 | 0.084 |
| Total number of conformer : 99530, Partition Index: 0.0021, Separation Index: 2.40E-08 | | | | | | | | | | |
| 129 | V1 | -87.803 | -12.843 | -52.794 |  |  |  | 7.26 | 0.312 | 0.049 |
| 130 | V2 | -65.416 | -43.004 | 168.77 |  |  |  | 28.50 | 0.190 | 0.047 |
| 131 | V3 | -111.44 | 124.14 | -173.66 |  |  |  | 17.30 | 0.170 | 0.008 |
| 132 | V4 | -69.349 | -41.751 | -170.83 |  |  |  | 4.58 | 0.194 | 0.027 |
| 133 | V5 | -123.51 | 153.34 | -53.799 |  |  |  | 13.09 | 0.320 | 0.085 |
| 134 | V6 | -108.45 | 126.71 | 170.37 |  |  |  | 29.28 | 0.263 | 0.057 |
| Total number of conformer : 151820, Partition Index: 0.001, Separation Index: 9.87E-09 | | | | | | | | | | |
| 135 | W1 | -72.025 | -14.996 | 63.363 | -87.685 |  |  | 6.06 | 0.325 | 0.065 |
| 136 | W2 | -116.25 | 136.68 | -68.587 | -76.754 |  |  | 14.26 | 1.131 | 0.299 |
| 137 | W3 | -64.952 | -37.487 | 168.14 | 75.165 |  |  | 9.80 | 0.614 | 0.212 |
| 138 | W4 | -71.368 | -27.338 | -69.821 | 103.97 |  |  | 15.22 | 0.370 | 0.055 |
| 139 | W5 | -62.471 | -41.706 | -168.81 | 80.36 |  |  | 6.58 | 0.314 | 0.089 |
| 140 | W6 | -72.518 | -23.709 | -66.421 | -16.91 |  |  | 8.90 | 0.429 | 0.079 |
| 141 | W7 | -90.491 | 136.08 | -65.997 | 89.713 |  |  | 13.36 | 0.508 | 0.233 |
| 142 | W8 | -64.175 | -37.517 | 168.61 | -102.35 |  |  | 6.74 | 0.434 | 0.082 |
| 143 | W9 | -64.164 | -35.801 | -165.14 | -101.32 |  |  | 5.46 | 0.427 | 0.074 |
| 144 | W10 | -118.18 | 140.7 | -56.733 | 72.332 |  |  | 13.61 | 0.836 | 0.448 |
| Total number of conformer : 29116, Partition Index: 0.0019, Separation Index: 1.06E-07 | | | | | | | | | | |
| 145 | Y1 | -80.429 | -20.716 | -67.526 | -64.872 |  |  | 16.25 | 0.568 | 0.206 |
| 146 | Y2 | -62.298 | -44.855 | 167.55 | 70.774 |  |  | 12.78 | 0.557 | 0.157 |
| 147 | Y3 | -111.32 | 137.51 | -60.034 | -78.785 |  |  | 16.70 | 0.625 | 0.319 |
| 148 | Y4 | -113.02 | 130.92 | 151.94 | 65.842 |  |  | 11.81 | 0.755 | 0.086 |
| 149 | Y5 | -114.67 | 140.18 | -59.911 | 84.664 |  |  | 16.00 | 0.329 | 0.052 |
| 150 | Y6 | -103.63 | 122.25 | -160.24 | 68.438 |  |  | 6.27 | 0.394 | 0.016 |
| 151 | Y7 | -82.448 | -18.398 | -65.626 | 95.415 |  |  | 11.84 | 0.457 | 0.075 |
| 152 | Y8 | -62.561 | -42.841 | -167.11 | 72.545 |  |  | 8.34 | 0.341 | 0.015 |
| Total number of conformer : 71876, Partition Index: 0.0018, Separation Index: 3.56E-08 | | | | | | | | | | |
