# Supplementary material for: Rationalization and Design of the Complementarity Determining Region Sequences in an Antibody-Antigen Recognition Interface
Source: PLoS One. 2012 Mar 22;7(3):e33340. doi: 10.1371/journal.pone.0033340 (PMC3310866; doi:10.1371/journal.pone.0033340)
Supplement: Table S7 — Atom types in protein structures. (DOC) [file pone.0033340.s008.doc]

**Table S7.** Atom types in protein structures.

| ID # | Atom Type | Radius | Description |
| --- | --- | --- | --- |
| 1 | NH1 | 1.65 | Backbone NH |
| 2 | C | 1.76 | Backbone C |
| 3 | CH1E | 1.87 | Backbone CA (exc. Gly) |
| 4 | O | 1.40 | Backbone O |
| 5 | CH0 | 1.76 | Arg CZ, Asn CG, Asp CG, Gln CD, Glu CD |
| 6 | CH1S | 1.87 | Sidechain CH1: Ile CB, Leu CG, Thr CB, Val CB |
| 7 | CH2E | 1.87 | Tetrahedral CH2 (except CH2P,CH2G) All CB |
| 8 | CH3E | 1.87 | Tetrahedral CH3 |
| 9 | CR1E | 1.76 | Aromatic CH (except CR1W, CRHH, CR1H) |
| 10 | OH1 | 1.40 | Alcohol OH (Ser OG, Thr OG1, Tyr OH) |
| 11 | OC | 1.40 | Carboxyl O (Asp OD1, OD2, Glu OE1, OE2) |
| 12 | OS | 1.40 | Sidechain O: Asn OD1, Gln OE1 |
| 13 | CH2G | 1.87 | Gly CA |
| 14 | CH2P | 1.87 | Pro CB, CG, CD |
| 15 | NH1S | 1.65 | Sidechain NH: Arg NE, His ND1, NE1, Trp NE1 |
| 16 | NC2 | 1.65 | Arg NH1, NH2 |
| 17 | NH2 | 1.65 | Asn ND2, Gln NE2 |
| 18 | CR1W | 1.76 | Trp CZ2, CH2 |
| 19 | CY2 | 1.76 | Tyr CZ |
| 20 | SC | 1.85 | Cys S |
| 21 | CF | 1.76 | Phe CG |
| 22 | SM | 1.85 | Met S |
| 23 | CY | 1.76 | Tyr CG |
| 24 | CW | 1.76 | Trp CD2, CE2 |
| 25 | CRHH | 1.76 | His CE1 |
| 26 | NH3 | 1.50 | Lys NZ |
| 27 | CR1H | 1.76 | His CD2 |
| 28 | C5 | 1.76 | His CG |
| 29 | N | 1.65 | Pro N |
| 30 | C5W | 1.76 | Trp CG |
| 31 | HOH | 1.40 | Water |
|  |  |  |  |
