# Supplementary material for: Rationalization and Design of the Complementarity Determining Region Sequences in an Antibody-Antigen Recognition Interface
Source: PLoS One. 2012 Mar 22;7(3):e33340. doi: 10.1371/journal.pone.0033340 (PMC3310866; doi:10.1371/journal.pone.0033340)
Supplement: Table S8 — Statistic pairwise atomistic interaction preferences. (DOC) [file pone.0033340.s009.doc]

**Table S8.** Statistic pairwise atomistic interaction preferences. This Table is derived from a more extensive statistics first published by McConkey et al. to adapt to the atom types in protein structures shown in Table S7.

|  | NH1 | C | CH1E | O | CH0 | CH1S | CH2E | CH3E | CR1E | OH1 | OC | OS | CH2G | CH2P | NH1S | NC2 | NH2 | CR1W | CY2 | SC | CF | SM | CY | CW | CRHH | NH3 | CR1H | C5 | N | C5W |
| --- | --- | --- | --- | --- | --- | --- | --- | --- | --- | --- | --- | --- | --- | --- | --- | --- | --- | --- | --- | --- | --- | --- | --- | --- | --- | --- | --- | --- | --- | --- |
| NH1 | 1.6 | 0.0 | 0.9 | -1.9 | 0.6 | 0.7 | 0.8 | 0.5 | 0.3 | -0.4 | -0.2 | -0.5 | 0.6 | 0.7 | 0.6 | 0.8 | 0.4 | 0.4 | 0.4 | -0.2 | 1.3 | 0.2 | 1.1 | 0.8 | 0.6 | 1.6 | 0.3 | 1.3 | 2.2 | 1.0 |
| C | 0.0 | 1.9 | 0.9 | 0.7 | 0.2 | 0.1 | 0.0 | -0.6 | -0.9 | 0.1 | 0.8 | 0.2 | -0.1 | -0.7 | 0.0 | 0.0 | 0.0 | -0.7 | 0.1 | -0.8 | 0.3 | -0.7 | 0.4 | 0.2 | -0.2 | 0.8 | -0.5 | 0.6 | 0.0 | 0.2 |
| CH1E | 0.9 | 0.9 | -0.4 | 0.0 | -0.5 | -0.9 | -0.6 | -0.6 | -0.8 | 0.0 | 0.0 | 0.0 | -0.3 | -0.2 | 0.0 | 0.0 | 0.0 | -0.7 | -0.9 | -0.7 | -0.5 | -1.0 | -0.6 | -0.7 | -0.4 | 0.5 | -0.6 | -0.5 | 0.7 | -0.8 |
| O | -1.9 | 0.7 | 0.0 | 0.6 | 0.1 | 0.0 | 0.0 | 0.0 | -0.2 | -0.2 | 1.0 | 0.4 | 0.0 | -0.4 | -0.4 | -0.3 | -0.4 | 0.0 | 1.0 | -0.1 | 0.3 | 0.4 | 0.5 | 1.0 | -0.2 | 0.0 | -0.5 | 0.1 | -1.4 | 0.3 |
| CH0 | 0.6 | 0.2 | -0.5 | 0.1 | -0.5 | -0.4 | -0.6 | -0.5 | -0.6 | 0.0 | 0.4 | 0.0 | -0.6 | -0.6 | 0.0 | 0.0 | 0.0 | -0.9 | -0.6 | -0.3 | -0.7 | -0.4 | -0.6 | -0.4 | -0.9 | 0.0 | -0.7 | -0.9 | 0.0 | -0.3 |
| CH1S | 0.7 | 0.1 | -0.9 | 0.0 | -0.4 | -0.7 | -0.4 | -0.7 | -0.7 | 0.0 | 0.2 | 0.0 | -0.3 | 0.0 | 0.0 | 0.1 | 0.0 | -0.6 | -0.7 | -0.8 | -0.6 | -0.8 | -0.5 | -0.6 | -0.3 | 1.4 | -0.4 | -0.8 | 0.5 | -0.7 |
| CH2E | 0.8 | 0.0 | -0.6 | 0.0 | -0.6 | -0.4 | -0.2 | -0.5 | -0.7 | 0.0 | 0.0 | 0.0 | -0.2 | -0.2 | 0.0 | 0.0 | 0.0 | -0.8 | -1.2 | -0.6 | -0.8 | -0.7 | -0.8 | -1.1 | -0.4 | 0.8 | -0.4 | -0.6 | 0.2 | -0.8 |
| CH3E | 0.5 | -0.6 | -0.6 | 0.0 | -0.5 | -0.7 | -0.5 | -1.1 | -1.0 | 0.0 | 0.4 | 0.0 | -0.4 | -0.4 | 0.0 | 0.0 | 0.0 | -0.9 | -1.0 | -0.8 | -1.3 | -1.1 | -1.2 | -1.1 | -0.4 | 0.9 | -0.6 | -0.9 | 0.0 | -1.1 |
| CR1E | 0.3 | -0.9 | -0.8 | -0.2 | -0.6 | -0.7 | -0.7 | -1.0 | -0.9 | -0.1 | 0.4 | -0.1 | -0.7 | -0.8 | -0.3 | -0.2 | -0.2 | -0.8 | -0.8 | -0.7 | -1.3 | -1.0 | -1.3 | -0.8 | -0.5 | 0.8 | -0.8 | -1.2 | -1.1 | -1.2 |
| OH1 | -0.4 | 0.1 | 0.0 | -0.2 | 0.0 | 0.0 | 0.0 | 0.0 | -0.1 | -0.5 | -0.8 | -0.5 | 0.0 | -0.5 | -0.9 | -0.3 | -0.6 | -0.1 | 0.5 | 0.1 | 0.7 | 0.2 | 0.4 | 0.7 | -0.5 | -0.2 | -0.6 | 0.0 | -0.5 | 0.5 |
| OC | -0.2 | 0.8 | 0.0 | 1.0 | 0.4 | 0.2 | 0.0 | 0.4 | 0.4 | -0.8 | 0.5 | 0.1 | 0.0 | 0.1 | -1.3 | -1.4 | -0.6 | 0.2 | 0.9 | 0.9 | 1.5 | 1.1 | 1.4 | 1.9 | -0.7 | -1.4 | -0.4 | 0.6 | 0.3 | 1.6 |
| OS | -0.5 | 0.2 | 0.0 | 0.4 | 0.0 | 0.0 | 0.0 | 0.0 | -0.1 | -0.5 | 0.1 | -0.1 | 0.0 | -0.3 | -0.8 | -0.8 | -0.8 | -0.3 | 0.4 | 0.2 | 0.3 | 0.3 | 0.5 | 0.2 | -0.5 | -0.6 | -0.5 | 0.0 | -0.2 | 0.0 |
| CH2G | 0.6 | -0.1 | -0.3 | 0.0 | -0.6 | -0.3 | -0.2 | -0.4 | -0.7 | 0.0 | 0.0 | 0.0 | -0.3 | -0.3 | 0.0 | 0.0 | 0.0 | -0.9 | -1.4 | -0.5 | -0.9 | -0.8 | -1.0 | -1.1 | -0.6 | 0.2 | -0.4 | -0.7 | 0.0 | -0.6 |
| CH2P | 0.7 | -0.7 | -0.2 | -0.4 | -0.6 | 0.0 | -0.2 | -0.4 | -0.8 | -0.5 | 0.1 | -0.3 | -0.3 | -0.1 | -0.4 | -0.2 | -0.2 | -1.0 | -1.5 | -0.5 | -1.2 | -0.8 | -1.4 | -1.6 | -0.6 | 0.9 | -0.5 | -1.1 | 0.1 | -1.4 |
| NH1S | 0.6 | 0.0 | 0.0 | -0.4 | 0.0 | 0.0 | 0.0 | 0.0 | -0.3 | -0.9 | -1.3 | -0.8 | 0.0 | -0.4 | 0.0 | 0.0 | 0.0 | -0.5 | 0.0 | -0.4 | 0.0 | -0.5 | 0.0 | 0.0 | -0.6 | 0.4 | -0.6 | 0.0 | 0.0 | -0.6 |
| NC2 | 0.8 | 0.0 | 0.0 | -0.3 | 0.0 | 0.1 | 0.0 | 0.0 | -0.2 | -0.3 | -1.4 | -0.8 | 0.0 | -0.2 | 0.0 | 0.0 | 0.0 | -0.4 | 0.0 | 0.3 | 0.0 | 0.2 | 0.0 | 0.0 | -0.4 | 0.5 | -0.2 | 0.0 | 0.0 | -0.9 |
| NH2 | 0.4 | 0.0 | 0.0 | -0.4 | 0.0 | 0.0 | 0.0 | 0.0 | -0.2 | -0.6 | -0.6 | -0.8 | 0.0 | -0.2 | 0.0 | 0.0 | 0.0 | -0.4 | 0.0 | 0.2 | 0.1 | -0.1 | 0.2 | 0.0 | -0.3 | 0.0 | -0.2 | 0.0 | 0.0 | 0.0 |
| CR1W | 0.4 | -0.7 | -0.7 | 0.0 | -0.9 | -0.6 | -0.8 | -0.9 | -0.8 | -0.1 | 0.2 | -0.3 | -0.9 | -1.0 | -0.5 | -0.4 | -0.4 | -0.5 | -0.8 | -0.3 | -1.2 | -0.9 | -1.1 | -0.2 | -0.8 | 0.4 | -1.1 | -1.7 | -0.8 | -1.0 |
| CY2 | 0.4 | 0.1 | -0.9 | 1.0 | -0.6 | -0.7 | -1.2 | -1.0 | -0.8 | 0.5 | 0.9 | 0.4 | -1.4 | -1.5 | 0.0 | 0.0 | 0.0 | -0.8 | 0.9 | -0.3 | -0.5 | -0.1 | -0.8 | 1.0 | -1.0 | 0.4 | -1.1 | -1.8 | 0.0 | -0.3 |
| SC | -0.2 | -0.8 | -0.7 | -0.1 | -0.3 | -0.8 | -0.6 | -0.8 | -0.7 | 0.1 | 0.9 | 0.2 | -0.5 | -0.5 | -0.4 | 0.3 | 0.2 | -0.3 | -0.3 | -3.2 | -0.5 | -0.7 | -0.3 | -0.3 | -0.3 | 1.0 | -0.4 | -0.4 | -0.9 | -0.5 |
| CF | 1.3 | 0.3 | -0.5 | 0.3 | -0.7 | -0.6 | -0.8 | -1.3 | -1.3 | 0.7 | 1.5 | 0.3 | -0.9 | -1.2 | 0.0 | 0.0 | 0.1 | -1.2 | -0.5 | -0.5 | -1.4 | -1.1 | -1.1 | -0.6 | -0.7 | 1.0 | -1.0 | -1.8 | 0.1 | -1.3 |
| SM | 0.2 | -0.7 | -1.0 | 0.4 | -0.4 | -0.8 | -0.7 | -1.1 | -1.0 | 0.2 | 1.1 | 0.3 | -0.8 | -0.8 | -0.5 | 0.2 | -0.1 | -0.9 | -0.1 | -0.7 | -1.1 | -0.7 | -0.9 | -0.3 | -0.9 | 0.8 | -1.0 | -0.9 | -1.0 | -0.8 |
| CY | 1.1 | 0.4 | -0.6 | 0.5 | -0.6 | -0.5 | -0.8 | -1.2 | -1.3 | 0.4 | 1.4 | 0.5 | -1.0 | -1.4 | 0.0 | 0.0 | 0.2 | -1.1 | -0.8 | -0.3 | -1.1 | -0.9 | -1.5 | -0.2 | -1.1 | 1.1 | -1.3 | -1.3 | 0.0 | -0.8 |
| CW | 0.8 | 0.2 | -0.7 | 1.0 | -0.4 | -0.6 | -1.1 | -1.1 | -0.8 | 0.7 | 1.9 | 0.2 | -1.1 | -1.6 | 0.0 | 0.0 | 0.0 | -0.2 | 1.0 | -0.3 | -0.6 | -0.3 | -0.2 | 0.9 | -0.7 | 0.5 | -1.5 | -1.9 | 0.0 | 0.0 |
| CRHH | 0.6 | -0.2 | -0.4 | -0.2 | -0.9 | -0.3 | -0.4 | -0.4 | -0.5 | -0.5 | -0.7 | -0.5 | -0.6 | -0.6 | -0.6 | -0.4 | -0.3 | -0.8 | -1.0 | -0.3 | -0.7 | -0.9 | -1.1 | -0.7 | -1.1 | 0.1 | -1.1 | -1.3 | -0.1 | -1.1 |
| NH3 | 1.6 | 0.8 | 0.5 | 0.0 | 0.0 | 1.4 | 0.8 | 0.9 | 0.8 | -0.2 | -1.4 | -0.6 | 0.2 | 0.9 | 0.4 | 0.5 | 0.0 | 0.4 | 0.4 | 1.0 | 1.0 | 0.8 | 1.1 | 0.5 | 0.1 | 0.6 | 0.4 | 1.0 | 1.0 | 0.8 |
| CR1H | 0.3 | -0.5 | -0.6 | -0.5 | -0.7 | -0.4 | -0.4 | -0.6 | -0.8 | -0.6 | -0.4 | -0.5 | -0.4 | -0.5 | -0.6 | -0.2 | -0.2 | -1.1 | -1.1 | -0.4 | -1.0 | -1.0 | -1.3 | -1.5 | -1.1 | 0.4 | -1.2 | -1.0 | -0.7 | -1.4 |
| C5 | 1.3 | 0.6 | -0.5 | 0.1 | -0.9 | -0.8 | -0.6 | -0.9 | -1.2 | 0.0 | 0.6 | 0.0 | -0.7 | -1.1 | 0.0 | 0.0 | 0.0 | -1.7 | -1.8 | -0.4 | -1.8 | -0.9 | -1.3 | -1.9 | -1.3 | 1.0 | -1.0 | -2.1 | 0.0 | -1.3 |
| N | 2.2 | 0.0 | 0.7 | -1.4 | 0.0 | 0.5 | 0.2 | 0.0 | -1.1 | -0.5 | 0.3 | -0.2 | 0.0 | 0.1 | 0.0 | 0.0 | 0.0 | -0.8 | 0.0 | -0.9 | 0.1 | -1.0 | 0.0 | 0.0 | -0.1 | 1.0 | -0.7 | 0.0 | 1.1 | -0.6 |
| C5W | 1.0 | 0.2 | -0.8 | 0.3 | -0.3 | -0.7 | -0.8 | -1.1 | -1.2 | 0.5 | 1.6 | 0.0 | -0.6 | -1.4 | -0.6 | -0.9 | 0.0 | -1.0 | -0.3 | -0.5 | -1.3 | -0.8 | -0.8 | 0.0 | -1.1 | 0.8 | -1.4 | -1.3 | -0.6 | 2.2 |

1. McConkey BJ, Sobolev V, Edelman M (2003) Discrimination of native protein structures using atom–atom contact scoring. Proceedings of the National Academy of Sciences of the United States of America 100: 3215-3220.
