# Supplementary material for: Rationalization and Design of the Complementarity Determining Region Sequences in an Antibody-Antigen Recognition Interface
Source: PLoS One. 2012 Mar 22;7(3):e33340. doi: 10.1371/journal.pone.0033340 (PMC3310866; doi:10.1371/journal.pone.0033340)
Supplement: Table S9 — The predicted ranking of the 20 natural amino acid types at each of the CDR amino acid positions in the 5 antibody-VEGF complex structures. (DOC) [file pone.0033340.s010.doc]

**Table S9.** The predicted ranking of the 20 natural amino acid types at each of the CDR amino acid positions in the 5 antibody-VEGF complex structures. Column 1 to column 7 from the left shows the PDB code name of the complex structure, the CDR amino acid position, the wild type amino acid type in the structure, the CDR, the core-rim assignment (see Figure 5 in the text) of the CDR amino acid, the number of predicted amino acid type with (*pW_ji_- t_i_*) >0, and the predicted ranking of the wild type amino acid type. The predicted ranking of the 20 natural amino acid types are shown following column 7. The wild type amino acid type is colored in red and the predicted amino acid types with (*pW_ji_- t_i_*) >0 are shown in blue background.

| PDBID | Site | Wild  type | CDR  classification | Interface  type | predicted  rank | wild type rank | 1 | 2 | 3 | 4 | 5 | 6 | 7 | 8 | 9 | 10 | 11 | 12 | 13 | 14 | 15 | 16 | 17 | 18 | 19 | 20 |
| --- | --- | --- | --- | --- | --- | --- | --- | --- | --- | --- | --- | --- | --- | --- | --- | --- | --- | --- | --- | --- | --- | --- | --- | --- | --- | --- |
| 1BJ1 | L32 | Y | L1 | rim | 8 | 3 | R | H | **Y** | N | G | T | A | L | P | E | D | S | I | F | M | C | W | V | Q | K |
| 1BJ1 | L91 | Y | L3 | core | 10 | 4 | S | A | H | **Y** | R | G | N | L | E | P | F | I | D | T | M | C | W | Q | K | V |
| 1BJ1 | L92 | S | L3 | rim | 10 | 2 | R | **S** | H | N | A | Q | P | L | Y | G | E | D | I | F | M | T | C | W | V | K |
| 1BJ1 | L93 | T | L3 | rim | 8 | 17 | R | S | H | A | N | P | G | L | D | Y | E | Q | I | M | F | C | **T** | K | W | V |
| 1BJ1 | L94 | V | L3 | rim | 10 | 19 | A | F | Y | P | L | H | E | N | S | R | D | G | M | W | I | T | C | K | **V** | Q |
| 1BJ1 | L95 | P | L3 | rim | 5 | 6 | R | G | H | A | N | **P** | L | T | D | Y | E | I | S | M | F | C | Q | W | K | V |
| 1BJ1 | L96 | W | L3 | core | 8 | 18 | R | H | L | A | G | N | Y | T | D | P | E | I | S | F | Q | M | C | **W** | K | V |
| 1BJ1 | H27 | Y | H1 | rim | 9 | 2 | R | **Y** | A | H | G | T | F | D | N | P | L | E | S | I | M | C | K | W | V | Q |
| 1BJ1 | H28 | T | H1 | rim | 6 | 20 | S | R | A | G | H | N | E | D | I | P | L | Y | M | F | C | K | W | V | Q | **T** |
| 1BJ1 | H29 | F | H1 | rim | 8 | 7 | R | A | H | G | T | D | **F** | N | L | P | E | Y | S | I | M | C | K | W | V | Q |
| 1BJ1 | H30 | T | H1 | rim | 6 | 20 | S | D | A | H | N | E | G | R | P | Y | L | I | M | F | C | K | V | W | Q | **T** |
| 1BJ1 | H31 | N | H1 | core | 9 | 3 | S | F | **N** | P | H | M | E | D | G | A | R | Y | L | V | I | C | K | W | T | Q |
| 1BJ1 | H32 | Y | H1 | core | 8 | 2 | D | **Y** | P | F | H | E | T | N | A | G | R | L | K | M | S | I | V | C | W | Q |
| 1BJ1 | H33 | G | H1 | core | 8 | 3 | D | Y | **G** | N | P | H | F | E | A | R | L | S | M | K | I | T | C | W | V | Q |
| 1BJ1 | H34 | M | H1 | rim | 10 | 14 | R | A | H | I | G | T | L | D | N | P | E | Y | S | **M** | F | C | K | W | V | Q |
| 1BJ1 | H35 | N | H1 | rim | 7 | 4 | R | H | S | **N** | A | G | Q | Y | T | P | D | L | E | I | M | F | C | W | K | V |
| 1BJ1 | H50 | W | H2 | core | 8 | 15 | R | G | N | H | Y | A | L | P | T | D | E | I | M | S | **W** | F | C | K | Q | V |
| 1BJ1 | H51 | I | H2 | rim | 9 | 3 | R | A | **I** | H | D | N | L | G | T | P | E | Y | S | F | M | C | W | K | Q | V |
| 1BJ1 | H52 | N | H2 | core | 7 | 1 | **N** | E | P | Y | H | A | T | L | F | G | R | D | M | I | S | K | C | V | W | Q |
| 1BJ1 | H53 | T | H2 | core | 7 | 18 | D | G | P | H | N | A | R | E | Y | L | I | S | M | K | F | C | V | **T** | W | Q |
| 1BJ1 | H54 | Y | H2 | rim | 8 | 2 | D | **Y** | H | N | P | G | T | A | L | R | M | E | S | F | I | K | C | V | W | Q |
| 1BJ1 | H55 | T | H2 | rim | 9 | 18 | S | R | N | A | E | H | G | P | Y | L | D | M | I | F | C | W | Q | **T** | V | K |
| 1BJ1 | H57 | E | H2 | rim | 6 | 10 | R | G | N | H | A | Y | T | P | L | **E** | I | D | S | F | M | C | Q | K | W | V |
| 1BJ1 | H58 | P | H2 | rim | 8 | 8 | R | S | A | H | D | G | N | **P** | I | E | L | Y | M | F | C | W | Q | K | V | T |
| 1BJ1 | H59 | T | H2 | core | 8 | 18 | N | H | Y | A | P | F | R | S | D | E | G | M | I | L | W | C | V | **T** | Q | K |
| 1BJ1 | H60 | Y | H2 | rim | 8 | 3 | R | H | **Y** | A | G | T | D | N | P | L | E | S | I | F | M | C | K | W | V | Q |
| 1BJ1 | H99 | Y | H3 | core | 11 | 4 | G | N | A | **Y** | P | H | D | R | L | E | T | I | S | M | F | C | K | W | V | Q |
| 1BJ1 | H100 | P | H3 | core | 8 | 10 | G | E | N | D | H | L | R | Y | T | **P** | A | S | I | M | F | C | W | K | V | Q |
| 1BJ1 | H101 | H | H3 | rim | 7 | 1 | **H** | P | E | Y | A | N | G | D | F | R | M | L | S | I | K | C | T | V | W | Q |
| 1BJ1 | H102 | Y | H3 | core | 8 | 2 | G | **Y** | P | D | E | H | F | N | K | A | T | R | L | I | S | M | C | V | W | Q |
| 1BJ1 | H103 | Y | H3 | rim | 10 | 2 | F | **Y** | N | G | H | A | P | D | E | L | R | M | I | S | C | W | T | Q | K | V |
| 1BJ1 | H104 | G | H3 | rim | 9 | 13 | P | A | F | N | R | L | H | Q | Y | M | E | D | **G** | I | S | T | C | W | K | V |
| 1BJ1 | H105 | S | H3 | rim | 10 | 2 | P | **S** | A | N | F | H | E | R | L | Y | D | M | G | I | T | Q | C | W | K | V |
| 1BJ1 | H106 | S | H3 | core | 6 | 15 | P | D | E | Y | N | H | A | F | K | R | M | T | L | G | **S** | I | V | C | W | Q |
| 1BJ1 | H107 | H | H3 | core | 11 | 2 | R | **H** | P | N | A | E | S | L | G | T | Y | D | M | I | F | C | K | W | V | Q |
| 1BJ1 | H108 | W | H3 | core | 10 | 15 | H | F | Y | G | P | E | N | A | L | R | D | M | S | I | **W** | T | C | K | V | Q |
| 1BJ1 | H109 | Y | H3 | rim | 6 | 4 | R | G | H | **Y** | N | A | D | P | L | E | T | I | S | F | M | C | W | Q | K | V |
| 1BJ1 | H110 | F | H3 | rim | 6 | 8 | R | H | G | A | T | N | D | **F** | P | L | Y | E | I | S | M | C | Q | W | K | V |
| 2QR0 | A91 | Y | L3 | core | 8 | 6 | A | S | R | H | G | **Y** | N | Q | I | D | P | E | L | T | F | M | C | W | K | V |
| 2QR0 | A92 | S | L3 | core | 9 | 1 | **S** | A | N | P | E | H | L | Y | D | F | R | G | I | M | C | T | Q | K | W | V |
| 2QR0 | A93 | Y | L3 | core | 9 | 8 | P | F | D | H | M | A | N | **Y** | S | R | E | L | G | I | K | V | C | T | W | Q |
| 2QR0 | A94 | Y | L3 | core | 9 | 6 | P | F | H | M | L | **Y** | N | A | D | G | R | E | I | S | W | C | T | Q | K | V |
| 2QR0 | A94A | Y | L3 | rim | 9 | 4 | R | P | F | **Y** | H | N | L | G | A | M | D | E | S | I | T | Q | W | C | K | V |
| 2QR0 | A94B | Y | L3 | rim | 5 | 10 | R | G | H | A | N | L | P | I | D | **Y** | E | S | T | Q | F | M | C | W | K | V |
| 2QR0 | B30 | S | H1 | rim | 7 | 2 | R | **S** | A | H | G | N | D | I | P | L | E | Y | M | F | C | K | W | V | Q | T |
| 2QR0 | B31 | S | H1 | rim | 8 | 1 | **S** | N | H | A | R | E | G | P | D | Y | L | F | M | I | C | V | W | T | K | Q |
| 2QR0 | B32 | S | H1 | core | 10 | 14 | Y | F | H | D | N | E | P | T | A | G | R | L | M | **S** | I | K | C | W | V | Q |
| 2QR0 | B33 | S | H1 | core | 8 | 14 | D | Y | F | N | P | E | A | H | G | R | L | W | M | **S** | T | K | I | C | V | Q |
| 2QR0 | B50 | Y | H2 | core | 8 | 4 | G | H | N | **Y** | A | R | L | F | I | S | E | D | P | M | T | C | W | K | Q | V |
| 2QR0 | B52 | Y | H2 | core | 9 | 5 | A | P | H | N | **Y** | F | G | L | S | I | E | R | D | M | T | K | C | W | Q | V |
| 2QR0 | B53 | S | H2 | rim | 7 | 1 | **S** | G | N | H | R | A | Y | L | P | I | D | T | E | Q | M | F | C | W | K | V |
| 2QR0 | B54 | Y | H2 | rim | 11 | 9 | P | F | N | A | S | M | L | E | **Y** | H | D | R | G | I | C | T | W | K | V | Q |
| 2QR0 | B55 | S | H2 | rim | 7 | 7 | R | G | H | N | Q | A | **S** | P | T | L | E | Y | D | I | M | F | C | W | K | V |
| 2QR0 | B56 | Y | H2 | core | 7 | 6 | P | A | N | H | F | **Y** | G | E | I | R | L | D | S | M | C | W | K | Q | T | V |
| 2QR0 | B58 | S | H2 | rim | 8 | 13 | H | Y | N | G | A | R | T | L | P | E | F | D | **S** | I | M | C | W | V | K | Q |
| 2QR0 | B95 | Y | H3 | core | 9 | 9 | A | H | P | N | R | G | L | F | **Y** | I | E | S | D | M | T | C | Q | W | K | V |
| 2QR0 | B96 | Y | H3 | core | 9 | 6 | P | F | N | A | H | **Y** | E | L | R | M | G | D | S | I | K | T | C | W | V | Q |
| 2QR0 | B97 | G | H3 | core | 9 | 6 | P | Y | F | H | N | **G** | A | L | D | R | E | M | S | I | T | C | W | K | V | Q |
| 2QR0 | B98 | T | H3 | rim | 11 | 15 | P | Y | F | N | A | H | M | R | D | L | E | S | G | I | **T** | C | W | V | K | Q |
| 2QR0 | B99 | G | H3 | core | 9 | 9 | F | P | H | N | A | Y | E | D | **G** | L | R | M | S | W | T | I | C | K | V | Q |
| 2QR0 | B100 | A | H3 | rim | 7 | 7 | R | G | H | Q | N | Y | **A** | P | L | S | D | E | I | T | F | M | C | W | K | V |
| 2QR0 | B100A | M | H3 | rim | 7 | 15 | R | H | G | A | N | D | L | P | I | Y | T | E | F | S | **M** | C | W | K | Q | V |
| 2FJH | L28 | V | L1 | rim | 6 | 20 | R | S | A | N | G | H | T | P | L | Y | E | D | I | Q | F | M | C | K | W | **V** |
| 2FJH | L29 | I | L1 | rim | 9 | 4 | R | A | H | **I** | G | L | T | D | N | P | E | Y | S | F | M | C | K | W | Q | V |
| 2FJH | L30 | R | L1 | rim | 10 | 10 | S | A | N | G | H | L | P | Y | E | **R** | D | M | I | F | T | C | Q | W | K | V |
| 2FJH | L31 | R | L1 | rim | 9 | 4 | S | A | N | **R** | G | H | P | E | Y | D | L | I | M | F | C | W | V | T | K | Q |
| 2FJH | L32 | S | L1 | core | 10 | 14 | Y | F | N | H | E | G | P | A | T | D | R | L | M | **S** | I | W | C | V | K | Q |
| 2FJH | L50 | A | L2 | rim | 9 | 6 | S | G | N | Y | H | **A** | L | R | P | E | F | T | D | M | Q | I | W | C | V | K |
| 2FJH | L51 | A | L2 | rim | 5 | 5 | R | G | H | N | **A** | D | P | E | L | Y | I | S | M | F | C | T | K | W | Q | V |
| 2FJH | L52 | S | L2 | rim | 8 | 1 | **S** | A | E | N | H | R | P | D | G | Y | F | L | M | I | T | C | W | K | V | Q |
| 2FJH | L53 | N | L2 | core | 8 | 1 | **N** | A | H | D | P | E | F | Y | G | R | S | L | I | M | C | W | V | K | Q | T |
| 2FJH | L54 | L | L2 | rim | 10 | 2 | R | **L** | A | D | H | E | T | G | N | S | P | Y | I | M | F | C | Q | K | W | V |
| 2FJH | L92 | N | L3 | core | 8 | 1 | **N** | D | E | F | Y | H | P | G | A | L | R | I | M | S | T | W | C | K | V | Q |
| 2FJH | L93 | T | L3 | rim | 10 | 16 | N | P | G | S | F | H | D | M | R | Y | A | E | L | I | C | **T** | V | W | Q | K |
| 2FJH | L94 | S | L3 | core | 8 | 14 | F | Y | P | D | H | E | N | A | L | G | R | I | M | **S** | C | K | T | W | V | Q |
| 2FJH | L95 | P | L3 | rim | 6 | 7 | R | G | H | A | N | L | **P** | Y | T | S | I | D | E | Q | F | M | C | W | K | V |
| 2FJH | L96 | L | L3 | core | 10 | 7 | G | Y | S | H | F | A | **L** | P | N | R | Q | D | M | E | T | I | W | C | K | V |
| 2FJH | H30 | N | H1 | rim | 7 | 6 | R | S | A | G | H | **N** | D | P | Y | L | E | I | F | M | C | W | V | Q | K | T |
| 2FJH | H31 | A | H1 | rim | 6 | 5 | S | G | N | H | **A** | R | E | P | L | Y | D | F | M | I | T | C | W | K | V | Q |
| 2FJH | H32 | S | H1 | rim | 7 | 5 | H | Y | A | G | **S** | N | R | L | P | D | E | I | T | F | M | C | K | W | V | Q |
| 2FJH | H33 | W | H1 | core | 7 | 15 | G | Y | H | N | A | F | P | R | D | E | L | S | M | T | **W** | I | C | K | V | Q |
| 2FJH | H52 | Y | H2 | core | 8 | 1 | **Y** | N | H | A | F | P | G | E | L | R | T | I | S | D | M | C | K | W | V | Q |
| 2FJH | H54 | Y | H2 | rim | 9 | 6 | S | R | P | N | D | **Y** | A | H | G | E | L | M | T | I | F | C | V | W | K | Q |
| 2FJH | H55 | S | H2 | rim | 9 | 3 | P | A | **S** | N | E | F | H | M | Y | L | D | R | G | I | V | C | K | T | W | Q |
| 2FJH | H56 | G | H2 | rim | 7 | 3 | R | S | **G** | H | A | N | T | P | E | L | Y | D | I | F | M | C | K | W | Q | V |
| 2FJH | H57 | Y | H2 | core | 7 | 1 | **Y** | N | P | H | A | G | F | R | E | M | L | D | I | T | S | K | C | W | V | Q |
| 2FJH | H99 | W | H3 | core | 7 | 15 | R | H | G | A | N | L | S | Y | I | P | E | D | F | M | **W** | T | C | K | Q | V |
| 2FJH | H100 | G | H3 | rim | 7 | 3 | R | H | **G** | N | A | L | S | Y | P | E | I | D | Q | T | F | M | C | W | K | V |
| 2FJH | H101 | H | H3 | rim | 10 | 7 | A | P | F | Y | E | N | **H** | M | D | L | R | S | G | Q | I | T | C | V | W | K |
| 2FJH | H102 | S | H3 | rim | 8 | 4 | A | R | H | **S** | P | N | Q | L | Y | E | G | D | M | F | I | C | W | T | K | V |
| 2FJH | H103 | T | H3 | core | 9 | 15 | R | S | H | N | A | P | L | Y | G | I | D | E | M | F | **T** | C | Q | W | K | V |
| 2FJH | H104 | S | H3 | core | 7 | 13 | P | N | H | A | Y | L | E | G | F | M | R | D | **S** | I | C | K | Q | W | T | V |
| 2FJH | H105 | P | H3 | core | 9 | 1 | **P** | Y | D | N | H | F | A | L | G | M | E | R | S | I | T | C | W | K | V | Q |
| 2FJH | H106 | W | H3 | core | 8 | 15 | F | Y | P | H | G | N | A | L | R | M | E | D | I | S | **W** | T | C | Q | K | V |
| 1TZH | L28 | A | L1 | rim | 7 | 3 | S | N | **A** | E | H | G | P | R | D | Y | F | L | I | M | C | K | W | V | Q | T |
| 1TZH | L29 | S | L1 | rim | 10 | 2 | R | **S** | A | D | F | N | H | E | P | L | I | G | Y | M | C | W | V | K | Q | T |
| 1TZH | L30 | Y | L1 | core | 6 | 12 | D | E | P | K | T | A | G | R | N | H | L | **Y** | F | V | M | S | C | I | W | Q |
| 1TZH | L31 | S | L1 | core | 8 | 11 | Y | A | F | N | P | H | E | T | D | R | **S** | L | G | M | K | I | C | W | V | Q |
| 1TZH | L32 | S | L1 | core | 8 | 12 | D | Y | P | H | N | A | F | E | G | R | L | **S** | M | K | W | I | T | C | V | Q |
| 1TZH | L50 | A | L2 | core | 9 | 6 | Y | P | N | F | H | **A** | D | E | L | R | M | G | S | T | W | I | C | V | K | Q |
| 1TZH | L53 | Y | L2 | rim | 8 | 7 | R | N | A | H | S | G | **Y** | Q | D | P | I | L | E | M | F | C | W | T | V | K |
| 1TZH | L91 | S | L3 | rim | 6 | 14 | R | H | G | Q | N | A | L | Y | P | I | E | T | D | **S** | F | M | C | W | K | V |
| 1TZH | L92 | S | L3 | core | 9 | 9 | P | A | H | N | D | F | L | Y | **S** | E | M | I | R | G | C | W | T | K | V | Q |
| 1TZH | L93 | A | L3 | core | 7 | 6 | P | N | H | F | M | **A** | D | E | Y | S | L | R | G | I | W | C | T | V | K | Q |
| 1TZH | L94 | S | L3 | rim | 9 | 14 | F | A | P | H | Y | N | L | G | M | R | D | E | I | **S** | C | W | T | K | V | Q |
| 1TZH | L96 | A | L3 | rim | 9 | 4 | R | G | H | **A** | S | N | Y | L | Q | P | D | E | T | F | M | I | C | W | K | V |
| 1TZH | H31 | D | H1 | rim | 6 | 11 | R | S | A | H | N | G | P | L | Y | E | **D** | I | T | F | M | C | W | K | V | Q |
| 1TZH | H32 | D | H1 | rim | 5 | 9 | R | A | H | Y | N | G | P | L | **D** | E | S | F | T | I | M | C | K | W | Q | V |
| 1TZH | H33 | D | H1 | core | 8 | 6 | Y | A | H | G | N | **D** | P | E | R | L | F | S | M | T | I | W | C | K | V | Q |
| 1TZH | H50 | Y | H2 | core | 8 | 6 | R | G | H | S | N | **Y** | A | L | I | P | D | E | F | M | C | T | W | K | Q | V |
| 1TZH | H52 | A | H2 | core | 9 | 7 | P | F | H | Y | N | E | **A** | L | I | G | R | D | M | S | K | T | C | W | V | Q |
| 1TZH | H53 | S | H2 | rim | 7 | 2 | G | **S** | N | H | R | A | Y | P | I | L | T | D | E | M | F | C | W | Q | K | V |
| 1TZH | H54 | Y | H2 | rim | 9 | 8 | F | P | N | D | H | E | L | **Y** | A | S | G | M | R | I | C | K | W | V | T | Q |
| 1TZH | H56 | Y | H2 | core | 7 | 6 | P | F | N | H | A | **Y** | G | D | I | E | L | R | M | S | C | K | W | T | V | Q |
| 1TZH | H58 | D | H2 | rim | 7 | 13 | H | Y | R | G | N | A | T | F | L | P | E | I | **D** | S | M | C | W | K | V | Q |
| 1TZH | H95 | S | H3 | rim | 7 | 2 | A | **S** | H | G | R | N | L | Y | P | F | E | D | T | M | I | C | W | K | Q | V |
| 1TZH | H96 | S | H3 | rim | 7 | 2 | R | **S** | H | G | N | A | L | P | Y | E | D | I | T | F | M | C | Q | W | K | V |
| 1TZH | H97 | D | H3 | rim | 8 | 11 | R | G | N | H | Y | A | T | L | P | E | **D** | S | M | I | F | C | W | K | Q | V |
| 1TZH | H98 | A | H3 | rim | 9 | 7 | R | H | G | Q | N | L | **A** | S | I | Y | P | D | T | E | F | M | C | W | K | V |
| 1TZH | H99 | S | H3 | rim | 8 | 4 | H | R | A | **S** | G | N | Y | L | P | I | T | E | D | M | F | C | W | Q | K | V |
| 1TZH | H100 | Y | H3 | rim | 10 | 3 | P | A | **Y** | M | F | N | E | L | H | D | R | I | G | S | T | C | W | V | K | Q |
| 1TZH | H100A | S | H3 | core | 10 | 4 | E | P | N | **S** | Y | D | F | M | A | H | R | G | L | I | W | K | C | T | V | Q |
| 1TZH | H100B | Y | H3 | core | 8 | 3 | P | F | **Y** | N | D | H | E | L | A | R | G | M | I | S | C | K | W | T | V | Q |
| 1TZH | H100C | S | H3 | rim | 11 | 3 | G | L | **S** | H | N | A | P | Y | F | R | Q | D | I | E | M | T | W | C | K | V |
| 1TZI | L28 | S | L1 | rim | 7 | 2 | R | **S** | G | H | Q | A | N | L | P | D | Y | E | I | M | F | T | C | W | K | V |
| 1TZI | L29 | Y | L1 | rim | 9 | 8 | S | N | G | A | P | H | R | **Y** | F | L | E | D | T | I | M | Q | C | W | K | V |
| 1TZI | L30 | A | L1 | rim | 8 | 3 | H | R | **A** | G | I | L | N | D | P | T | Y | E | S | M | F | Q | C | K | W | V |
| 1TZI | L31 | Y | L1 | rim | 6 | 8 | R | S | H | A | N | G | Q | **Y** | L | P | E | D | I | F | M | C | T | W | V | K |
| 1TZI | L32 | A | L1 | rim | 7 | 6 | R | H | N | Y | G | **A** | T | L | P | D | E | S | I | F | M | C | W | V | K | Q |
| 1TZI | L50 | D | L2 | rim | 8 | 11 | R | G | S | H | Y | N | A | L | P | E | **D** | T | F | M | W | I | C | Q | V | K |
| 1TZI | L53 | Y | L2 | rim | 9 | 7 | N | E | D | A | H | P | **Y** | R | F | G | S | L | M | I | C | W | V | K | Q | T |
| 1TZI | L92 | Y | L3 | rim | 8 | 5 | R | G | H | N | **Y** | A | S | L | P | I | E | D | T | F | M | W | Q | C | K | V |
| 1TZI | L93 | S | L3 | rim | 7 | 2 | R | **S** | G | H | N | A | L | Y | I | P | D | E | Q | T | M | F | C | W | K | V |
| 1TZI | H28 | A | H1 | rim | 7 | 6 | R | S | N | H | G | **A** | P | E | I | D | L | Y | M | F | C | K | W | V | Q | T |
| 1TZI | H30 | Y | H1 | rim | 6 | 8 | R | S | A | G | H | N | D | **Y** | E | P | L | I | M | F | C | W | V | Q | K | T |
| 1TZI | H31 | D | H1 | core | 6 | 8 | N | H | P | S | G | E | A | **D** | R | F | Y | L | M | K | I | V | C | T | W | Q |
| 1TZI | H32 | Y | H1 | core | 9 | 1 | **Y** | F | H | P | N | A | G | E | D | R | L | S | M | I | K | C | T | W | V | Q |
| 1TZI | H33 | D | H1 | core | 7 | 9 | R | H | G | Y | A | N | P | E | **D** | L | S | T | F | I | M | W | C | K | V | Q |
| 1TZI | H52 | A | H2 | rim | 8 | 4 | H | N | R | **A** | G | L | T | F | I | P | S | Y | E | D | M | C | Q | K | W | V |
| 1TZI | H53 | Y | H2 | core | 6 | 5 | P | A | M | N | **Y** | H | D | R | L | F | E | G | I | S | T | C | W | V | K | Q |
| 1TZI | H54 | A | H2 | core | 8 | 9 | F | D | N | P | H | G | L | Y | **A** | M | S | R | E | I | C | T | W | V | K | Q |
| 1TZI | H56 | A | H2 | rim | 7 | 6 | R | H | G | L | N | **A** | I | Q | Y | S | P | D | E | F | M | C | T | W | K | V |
| 1TZI | H95 | S | H3 | rim | 7 | 5 | R | H | G | A | **S** | L | N | I | Y | P | T | D | E | F | M | C | W | Q | K | V |
| 1TZI | H96 | S | H3 | core | 7 | 13 | G | H | N | P | F | Y | A | E | R | L | M | D | **S** | I | C | T | K | V | W | Q |
| 1TZI | H97 | Y | H3 | rim | 10 | 2 | P | **Y** | D | N | H | M | G | E | L | A | F | R | S | I | C | W | T | V | K | Q |
| 1TZI | H98 | A | H3 | core | 7 | 11 | D | G | P | F | H | N | Y | M | E | L | **A** | I | R | S | K | C | W | V | T | Q |
| 1TZI | H99 | Y | H3 | core | 9 | 5 | R | H | F | N | **Y** | A | L | G | P | S | D | E | I | M | Q | T | C | W | K | V |
| 1TZI | H100 | Y | H3 | rim | 9 | 4 | G | H | N | **Y** | A | R | P | Q | L | F | E | M | D | T | S | I | C | W | K | V |
| 1TZI | H100A | A | H3 | rim | 7 | 6 | R | G | H | Q | N | **A** | Y | L | P | D | E | I | S | T | F | M | C | W | K | V |
| 1TZI | H100B | A | H3 | rim | 7 | 6 | R | H | G | N | Q | **A** | Y | L | P | D | E | I | S | T | F | M | C | W | K | V |
