# Supplementary material for: Rationalization and Design of the Complementarity Determining Region Sequences in an Antibody-Antigen Recognition Interface
Source: PLoS One. 2012 Mar 22;7(3):e33340. doi: 10.1371/journal.pone.0033340 (PMC3310866; doi:10.1371/journal.pone.0033340)
Supplement: Text S1 — Supplemental Methods. (DOC) [file pone.0033340.s011.doc]

**Supplemental Methods**

***1. Amino acid conformation clustering***

Amino acids in proteins are limited in structural diversity. Protein structures are determined by mainchain and sidechain torsion angles of the constituent amino acids. The distributions of the torsion angles are clustered around prevalent conformational centers, instead of spreading continuously over the torsion angle space. The mainchain torsion angles are clustered at the α- and the β-regions in the Ramachandran plot; the distributions of the sidechain torsion angles are also concentrated on only a few allowable regions, depending on the chemical constituents of the sidechain . Moreover, the distribution of each of the sidechain torsion angles is dependent on the torsion angles of the backbone of the amino acids . Thus, amino acid conformations in proteins can be organized into limited sets of clusters based on the mainchain and sidechain torsion angle set of each of the amino acid types, allowing interacting atom pair database retaining conformational information of the parent amino acids.

Database for non-covalent interacting atom pairs in proteins was organized according to parent amino acid conformational types. To cluster amino acid conformations into a limited set of clusters for each type of amino acid, we assigned torsion angles to each of the amino acids in known protein structures with the computer program DSSP and MOLEMAN 2 . For each type of amino acid from the protein structure entries in PDB, a set of vectors with torsion angle elements in degree ({*φ*, *ψ*, χ*_1_*, …, χ*_i_*}, where *φ*, *ψ* are backbone torsion angles and χ*_i_* are sidechain torsion angles as defined conventionally) was established; amino acid residues with incomplete structure were excluded from the data sets. The vectors were used as input to the fuzzy c-means algorithm for clustering. The number of the clusters was determined as the minimal integer satisfying the condition that increasing the number of clusters beyond this minimal integer made little change to the partition index and separation index – two fuzzy c-means algorithm indexes describing the relative mean distance within and between clusters . To augment the optimal decision on cluster numbers, we calculated the distribution of the intra-cluster RMSD (root mean squared deviation) in Å for superimposed amino acid structures between cluster members and the centroid conformation within a cluster for each cluster sets. The convergence of this intra-cluster RMSD to a minimal RMSD provided a more structure-related reference in contrast to the torsion angle-based structural descriptors in determining the optimal cluster number. After the determination of the cluster numbers, the centroid conformation of each of the clusters was determined as the center of mass of the vectors in the cluster. The number of clusters, the torsion angles of the centroid conformations, and the distribution information of the members in the clusters are listed in Table S6.

***2. Protein atomistic non-covalent interacting database***

Atomistic contact interactions in proteins of known structures were organized into a database containing non-covalent atomistic interaction information for atom pairs in protein structures. The general methodology is outline in Figure S1. For each of the atoms in residue X of a protein, the non-covalent interacting atoms were recorded as the following: Following the work of Laskowski et al. , for each atom (P) in residue X, the relative location of the atom P was defined with two consecutive atoms R and Q, where R is covalently linked to P, and Q is covalently linked to R. Atom R was set at the origin of the reference coordinate system; atom P was located on the z-axis; atom Q was on the z-x plane of the reference coordination system. In principle, all non-covalent interacting atoms to atom P were recorded in the database with the reference coordination system. In this work, only non-covalent atomistic interactions in protein interiors were organized into the atomistic interaction database: First, a protein structure was randomly separated into two parts by cleaving at a random peptide bond. Interface residues with solvent accessible surface area (SASA) change more than 40% of the total SASA due to the separation of the two protein halves were considered for non-covalent atomistic interactions. The solvent accessible surface area (SASA) for each of the amino acid residues was calculated with DSSP. Only the atoms from the other half of the proteins were recorded for interacting with atom P when the pairwise distance between the two atoms was less than 5 Å. Atoms within 9 consecutive residues from the N and C directions of the atom P were excluded as interacting atoms to the atom P. This was to record the atomistic contact interactions mimicking the interactions in protein-protein interfaces. After all the interface residues were surveyed, the protein structure was again randomly separated at a different cleavage site and the survey for the atomistic contact interactions of each of the interface residues was repeated. This process repeated 40 times for each of the protein structures in the 9468 non-redundant protein structures with less than 60% sequence identity . After the survey on all the non-covalent interacting atom pairs, the database was organized into a large number of files; each file is specific to an amino acid type, a conformational type based on the torsion angle vector of the amino acid, an atom type in the parent amino acid, and the interacting atom type. The structure of the data files facilitates the speedy random access of the database in predicting distribution of probability density maps (PDM) of non-covalent interacting atoms as described in the following section. Atoms in the 20 natural amino acids are assigned to one of the 30 interacting atom types found in proteins plus the crystal water oxygen as the 31^st^ atom type (Table S7).

Water oxygen distributions around the surface amino acids in 915 non-redundant protein structures solved to high resolution (resolution<1.5Å, sequence identity less than 30%, different graph topology and subunit structure) were recorded with the same P-R-Q reference coordination system and were stored in the same file system as described above. Water oxygens within 3.2 Å radius (within hydrogen bonding distance) to the interacting amino acid atoms were recorded in the database. This database was used for evaluating the desolvation penalties and water-mediated interactions in protein-protein interaction interfaces.

***3. Predicting probability density maps (PDM) of non-covalent interacting atoms for protein surfaces***

A probability density map (PDM) of a non-covalent interacting atom type is a three-dimensional distribution of likelihood for the type of atom to appear around protein surface amino acids. In this work, the PDMs were reconstructed from the interacting atom pair databases described in the previous section for the 31 interacting atom types shown in Table S7. The general methodology is outline in Figure S1.

To construct a PDM for an interacting atom type on a target protein surface, the computer algorithm first enclosed the target protein structure in a rectangular box clearing the structure by a margin of at least 7 Å from all sides of the protein’s edge. The three-dimensional rectangular box was then gridded with 0.5 Å per unit in three-dimensional space. This grid size was a balance between the resolution of the PDM and the computational resources needed for the PDM construction. The grid points enclosed within the Connolly surface of the target protein were masked from assigning PDM.

The torsion angles of sidechain and mainchain of all the amino acids in the protein structure were calculated with MOLMAN2 and DSSP respectively. For each of the amino acid residues in the protein, the conformational type of the amino acid X was determined by the torsion angle vector, which had the least Euclidean distance to the centroid conformation of the assigned conformational cluster. With the assignment of the conformational type for each of the amino acids in the protein structure, the non-covalent interacting atoms around each atom P in the protein structure were allocated from the database according to the atom type of P, the assigned three-atom reference system P-R-Q as described in the previous section, the amino acid type of the parent residue containing atom P, and the conformational type of the parent amino acid. Interacting atoms outside the sphere with the radius equal to the sum of the van der Waals radii of the interacting atom and atom P plus a tolerance of 0.5 Å were not included as the interacting atoms with atom P. The coordinates of the allocated interacting atoms were transformed to the coordination system of the protein structure and mapped around the protein surface. An atom of non-covalent interaction was to be mapped only once for which the distance of the atom to P was the shortest. 31 PDMs were constructed from all the interacting atoms allocated for all the protein atoms (30 atom types) in the protein structure.

In order to keep PDMs high in information content and low in noise from irrelevant interactions, two strategies have been implemented. First, allocation of interacting atoms according to the amino acid conformational type (as described above) is crucial for retaining information content in PDMs. Alternative approach for PDM construction with interacting atoms allocated from mixed amino acid conformational types would lead to loss of fidelity in relative orientations of the interacting atoms, resulting in spreading PDMs around dihedral bonds. We found that mapping interacting atoms obtained from an atom in an amino acid conformational type onto the surroundings of the atom in another amino acid conformational type led to serious spatial distortion of the distribution of the interacting atoms. Second, only interacting atomic pairs in the database are used for PDM constructions. Atom pairs in the database were recorded by a threshold of distance in proximity. But frequently, many of such distributions of proximal atom pairs are results of covalent structures of non-interaction pairs in a nevertheless stable structure. In this work, non-interacting atomic pairs were eliminated with a filter Table as shown in Table S8 . Only the atomic pairs with the value in the matrix of the Table less than -0.1 were considered as interacting pairs and only these interacting atoms were included in the PDM constructions.

PDMs were constructed by mapping the interacting atoms allocated from the database as described in the previous paragraphs to the 3D grid system. To construct the PDM, each of the interacting atoms was distributed to 8 nearest grid points; the portion of the distribution was normalized by the database redundancy and was inversely proportional to the square of the distance from the atom to the grid:

$v_{ji}=\frac{1}{p_{i}n}\frac{\frac{1}{d_{ji}^{2}}}{\sum_{k=1}^{8} \frac{1}{d_{ki}^{2}}}$ (1)

, where *v_ji_* is the value to be accumulated at a nearest grid point *j* for interacting atom *i*; *d_ji_* is the distance of grid point *j* to the center of the interacting atom *i*; grid points indexed *k*=1~8 are the nearest grids to the atom *i*; *n* is the number of residues collected in the database for the amino acid in the target protein with the conformational type defined by the torsion angle vector; *p_i_* is the background probability for atom type *i* to appear in all protein structures (when calculating water oxygen PDM, *p_i_* equals to 1). The factor 1/*n* in the Equation is to normalize the interacting atom density according to one conformation for each of the residues in the target protein and the background probability *p_i_* is to normalize the PDM based on the appearance frequency of the atom type *i* in proteins (except for water oxygen). The PDM for each of the interacting atom types was additively accumulated to completion as each of the atoms in the target protein surface finished contributing to the PDMs.

PDMs constructed for 31 interacting atomic types on the surface of 20 natural amino acids and their various conformations are displayed online: http://ismblab.genomics.sinica.edu.tw/introduction/diaa/.

***4. Computation of the LOGO plots***

The size *d_ji_* (in half-bit unit) for amino acid type *i* at position *j* in the consensus sequence LOGOs shown in Figure 1 were calculated with the equation:

$$d_{ji}=q_{ji}I_{j}$$

$$I_{j}=\sum_{i=1}^{20} 2q_{ji}\log_{2}\frac{q_{ji}}{p_{i}}$$

$q_{ji}=\frac{C_{ji}+\sqrt{M_{j}}p_{i}}{(M_{j}+\sqrt{M_{j}})}$ (2)

*C_ji_* is the count for amino acid *i* at position *j* in *M_j_* count of VEGF-binding CDR sequences containing position *j*; *p_i_* is the background probability for amino acid *i* encoded in the NNK degenerate codon ; the square root of *M_j_* in the equation is the pseudo count to prevent singularity when *C_ji_* equals to zero. Equation (2) is modified after the original formulation .

***5. Mapping interacting strength of the antibodies against VEGF***

Model structures of the selected VEGF-binding scFv variants, for which the sequence are shown in Table S1, were constructed with SCWRL 4.0 with the backbone fixed as in 2FJG for 5 consecutive CDR residues in each of the 6 CDRs. These are the positive scFv variant groups for VEGF-binding. For each of the CDRs, 1000 models with random sequences were also constructed with the same modeling procedure. Experiments showed that all libraries of scFv with random CDR sequences did not bind to VEGF collectively. These scFv variants are the negative groups for VEGF-binding. For each of the model structures, the probability density maps for all 30 interacting atom types were constructed as described in section 3. For each of the VEGF interface atoms, an averaged p-value was calculated from the distributions of the scores derived from the probability density maps constructed based on the VEGF-binding scFv models (positive scFv variant group) against the distributions of the scores derived from the probability density maps constructed based on the scFv models with random CDR sequences (negative scFv variant group). The averaged p-value on the VEGF interface atom is the arithmetically averaged odd for a random CDR sequence to confer equal or better score on the atom than a VEGF-binding sequence does. A decreasing averaged p-value (colored in increasing redness) for the interface VEGF atom suggests that the atom is more consistently used to bind to antibody variants with increasingly stronger interactions governing the interactions in protein interior cores (calculated with the *X_ji_* term); on the other hand, a interface atom with p-value close to 1 (colored in blue) would be the locations where protein interior-type interactions are preferred to be avoided in the antibody-VEGF interface. Interface atoms colored in white are indifferent in terms of the interactions. Grey atoms are non-interface atoms.

***6. Mapping hydration pattern on a protein surface***

For each surface atom *a_i_* on the protein, the water probability densities on grids within 5 Å to the atom were summed as *h_i_*, which was assigned to the center of the atom *a_i_*. The patch-averaged hydration *H_i_* for atom *a_i_* was calculated with the equation:

$H_{i}=h_{i}+\sum_{j=1,j\neq i}^{n} h_{j}\frac{d_{ji}^{-2}}{\sum_{k=1,k\neq i}^{n} d_{ki}^{-2}}$ (3)

, where *a_j_* is a surface atom within 10 Å of radius centered at atom *a_i_*; *n* is the total number of surface atoms within the 10 Å radius of the central atom *a_i_*; *d_ji_* is the distance between atom *j* and atom *i (ji)*. Surface atoms were the atoms with solvent accessible surface area greater than 0. The surface atoms were then color-coded in terms of the surface hydration measurement *H*; white color codes for the medium value between the maximum and minimum of *H*.

**Supplemental References**

1. Lovell SC, Word JM, Richardson JS, Richardson DC (2000) The penultimate rotamer library. Proteins: Structure, Function, and Bioinformatics 40: 389-408.

2. Dunbrack RL (2002) Rotamer Libraries in the 21st Century. Current Opinion in Structural Biology 12: 431-440.

3. Dunbrack RL, Jr., Karplus M (1994) Conformational analysis of the backbone-dependent rotamer preferences of protein sidechains. Nat Struct Biol 1: 334-340.

4. Kabsch W, Sander C (1983) Dictionary of protein secondary structure: pattern recognition of hydrogen-bonded and geometrical features. Biopolymers 22: 2577-2637.

5. Kleywegt GJ (2007) Quality control and validation. Methods Mol Biol 364: 255-272.

6. Bezdek J (1981) Pattern Recognition with Fuzzy Objective Function Algorithms (Advanced Applications in Pattern Recognition): Springer.

7. Bensaid AMH, L.O.; Bezdek, J.C.; Clarke, L.P.; Silbiger, M.L.; Arrington, J.A.; Murtagh, R.F. (1996) Validity-guided (re)clustering with applications to image segmentation. IEEE Transactions on Fuzzy Systems 4: 22.

8. Laskowski RA, Thornton JM, Humblet C, Singh J (1996) X-SITE: Use of Empirically Derived Atomic Packing Preferences to Identify Favourable Interaction Regions in the Binding Sites of Proteins. Journal of Molecular Biology 259: 175-201.

9. Li MH, Lin L, Wang XL, Liu T (2007) Protein-protein interaction site prediction based on conditional random fields. Bioinformatics 23: 597-604.

10. Levy ED, Pereira-Leal JB, Chothia C, Teichmann SA (2006) 3D Complex: A Structural Classification of Protein Complexes. PLoS Comput Biol 2: e155.

11. Connolly M (1983) Analytical molecular surface calculation. Journal of Applied Crystallography 16: 548-558.

12. McConkey BJ, Sobolev V, Edelman M (2003) Discrimination of native protein structures using atom–atom contact scoring. Proceedings of the National Academy of Sciences of the United States of America 100: 3215-3220.

13. Hsu H-J, Chang H-J, Peng H-P, Huang S-S, Lin M-Y, et al. (2006) Assessing Computational Amino Acid ²-Turn Propensities with a Phage-Displayed Combinatorial Library and Directed Evolution. Structure (London, England : 1993) 14: 1499-1510.

14. Gorodkin J, Heyer LJ, Brunak S, Stormo GD (1997) Displaying the information contents of structural RNA alignments: the structure logos. Comput Appl Biosci 13: 583-586.
